# Core outcome set for behavioural weight management interventions for adults with overweight and obesity: Standardised reporting of lifestyle weight management interventions to aid evaluation (STAR‐LITE)

**DOI:** 10.1111/obr.12961

**Published:** 2019-11-22

**Authors:** Ruth M. Mackenzie, Louisa J. Ells, Sharon Anne Simpson, Jennifer Logue

**Affiliations:** ^1^ Institute of Cardiovascular and Medical Sciences University of Glasgow Glasgow UK; ^2^ School of Health and Social Care Teesside University Middlesbrough UK; ^3^ Institute of Health and Wellbeing University of Glasgow Glasgow UK

**Keywords:** adult behavioural weight management interventions, core outcome set, standardised reporting

## Abstract

Behavioural weight management interventions in research studies and clinical practice differ in length, advice, frequency of meetings, staff, and cost. Few real‐world programmes have published patient outcomes and those that have used different ways of reporting information, making it impossible to compare interventions and develop the evidence base. To address this issue, we have developed a core outcome set for behavioural weight management intervention programmes for adults with overweight and obesity. Outcomes were identified via systematic review of the literature. A representative expert group was formed comprising people with experience of adult weight management services. An online Delphi process was employed to reach consensus as to which outcomes should be measured and reported and which definitions/instruments should be utilised. The expert group identified eight core outcomes and 12 core processes for reporting by weight management services. Eleven outcomes and five processes were identified as optional. The most appropriate definitions/instruments for measuring each outcome/process were also agreed. Our core outcome set will ensure consistency of reporting. This will allow behavioural weight management interventions to be compared, revealing which interventions work best for which members of the population and helping inform development of adult behavioural weight management interventions.

**Abbreviations**
BMI: body mass index
BWMI: behavioural weight management intervention
COMET: Core Outcome Measures in Effectiveness Trials
COS: core outcome set
COS‐STAR: Core Outcome Set‐STAndards for Reporting
CSO: Chief Scientist Office
EOSS: Edmonton Obesity Scale Score
HbA1c: haemoglobin A1c
IPR: inter‐percentile range
IPRAS: inter‐percentile range adjusted for symmetry
KPI: key performance indicators
MRC: Medical Research Council
NHS: National Health Service
NICE: National Institute for Health and Care Excellence
NPRI: National Prevention Research Initiative
PHE: United Kingdom, UK; Public Health England
QoL: quality of life
RAND: Research and Development
SEF: standard evaluation framework
SIGN: Scottish Intercollegiate Guidelines Network
STAR‐LITE: STAndardised Reporting of Lifestyle Weight Management InTerventions to Aid Evaluation
UCLA: University of California Los Angeles
USA: United States of America

## 1 INTRODUCTION

Behavioural weight management interventions (BWMIs), known in the United Kingdom (UK) as tier 2 services, are the first line treatment for overweight and obesity^1^, ^2^, ^3^, ^4^. International guidelines, including those of The National Institute for Health and Care Excellence (NICE)^1^, Scottish Intercollegiate Guidelines Network (SIGN)^2^, and the American College of Cardiology/American Heart Association Task Force on Practice Guidelines and The Obesity Society^3^, outline the intervention components to be included in a behavioural weight management programme for adults. These components, which include calorie restriction, increased physical activity and behavioural change support, have proven efficacy in randomised controlled trials^3^. However, their implementation in practice is inconsistent. Indeed, mapping exercises in Scotland^4^ and England^5^ revealed wide variation in adult weight management services with regard to inclusion criteria, referral routes, delivery format, programme length and cost, despite the single‐payer health care system. Furthermore, few adult BWMIs have published outcome data and where these data are published, results are often poor with low levels of programme completion and “success,” with a lack of longer term outcomes^6^, ^7^.

When developing the guidance, “Weight management: lifestyle services for overweight or obese adults”^1^ in 2014, NICE identified a number of evidence gaps. These included reliance on studies with short follow‐up, collection of data at limited time points, small sample sizes, demographic samples that limit the ability to generalise, nonreporting of reasons for people dropping out and lack of evidence regarding the effect of population characteristics, such as age, gender, and socio‐economic status, on the effectiveness of a service. NICE specifically mentioned “variable outcome definitions” used in the clinical trials, which formed the supporting systematic review and meta‐analysis, as a major barrier to developing evidence‐based guidance. As a result, they were left with many evidence gaps including “a lack of trials directly comparing lifestyle weight management programmes in the UK” and “a general lack of evidence on which specific components of a lifestyle weight management programme ensure effectiveness.” This lack of an evidence base from both clinical trials and real‐world services means that it is not possible to issue clear guidance as to which services are cost effective for which population groups.

Public health bodies in the United Kingdom have made efforts to try and address this issue; Public Health England (PHE)^8^ created a standard evaluation framework (SEF) for weight management programmes^9^. However, PHE was unable to analyse data from real world interventions due to the heterogeneity of reporting, suggesting further guidance is required. This heterogeneity can be exemplified by reporting of weight loss, which included number of kilograms lost, percentage weight loss, average number of completers achieving 5% weight loss, and body mass index (BMI)^5^. With regard to clinical trials, evidence suggests similarly heterogeneous reporting of outcomes^7^.

It is acknowledged that the provision of treatments for obesity is severely limited across the world,^10^, ^11^, ^12^, ^13^, ^14^ and large gaps in the evidence of effectiveness may be contributing to this. An improved evidence base would allow intervention programmes to be commissioned and funded by health systems with the confidence of effectiveness. There is an urgent need to gain consensus on standardised outcome reporting to allow better comparison and meta‐analysis of interventions to be performed across both real world and trial interventions. Therefore, the specific aim of this study was to use Delphi methodology to gain expert consensus opinion on the core outcomes that should be reported from BWMIs in real‐world clinical practice as well as within research studies and on the outcome definitions/outcome measurement instruments that should be used in their evaluation. Core outcome set (COS) development has an established methodology,^15^ and COS represent the minimum that should be reported in all clinical trials of a specific condition, while also being suitable for observation research and audit; their use in clinical trials is supported by the UK National Institute of Health Research (NIHR)^16^ as it allows trial results to be easily compared and combined. However, the development of a COS does not imply that research outcomes should be restricted to only those included in the COS. The development of these core outcome and definition/instrument sets for BWMIs will ensure more consistency in the measurement of the effectiveness of weight management services, leading to a better evidence base from which to identify which services are effective across a range of settings.

## 2 METHODS

### 2.1 Ethics

Ethical approval for this study was received from the University of Glasgow College of Medical, Veterinary and Life Sciences Ethics Committee.

The project has been registered with the COMET (Core Outcome Measures in Effectiveness Trials) Initiative (http://www.comet‐initiative.org/studies/details/1056), and a detailed methodology has been reported previously^17^. In reporting the development of our COS, we have adhered to the COS‐STAR (Core Outcome Set‐STAndards for Reporting) Statement (Table S1)^18^.

### 2.2 Identification of outcomes

In order to develop a COS, a comprehensive list of outcomes for reporting from BWMIs was generated. These outcomes were identified following review of studies included in the systematic review, “The clinical effectiveness of long‐term weight management schemes for adults” by Hartmann‐Boyce et al^7^, conducted during the development of NICE guidance^1^. This review was updated to cover the time period 1 November 2012, until 30 September 2017, using the same inclusion criteria (inclusion criteria and additional studies are outlined in Section S1). Both primary and secondary outcomes from studies were identified by two independent researchers and entered into a spreadsheet. Additionally, the PHE SEF^9^, minimum dataset,^19^ and key performance indicators (KPI) document^20^ were reviewed, again by two independent researchers, and any supplementary outcomes added to the aforementioned spreadsheet. Of note, the PHE SEF^9^ was developed following focus group work with a wide range of stakeholders, including weight management staff, primary care staff, academics, commissioners, and policy makers, and has been refined over two versions from 2009 to 2018.

### 2.3 Identification of outcome measurement instruments/outcome definitions

Analyses of studies identified during the systematic review by Hartmann‐Boyce^7^ and our updated search (Section S1) allowed instruments and definitions for selected outcomes to be added to the data extraction spreadsheet by two independent researchers. This list was then examined by all study investigators and further suitable instruments/definitions added.

### 2.4 Participants

The core outcome and instrument set was developed by means of consensus from an expert group, recruited as outlined previously^17^ and selected based on our sampling framework (Section S2) to ensure a representative sample and a pragmatic and patient‐centred COS. All experts recruited were from the United Kingdom.

For the stage 1 (outcome selection) Delphi process, agreement to participate was obtained from 10 members of the public with experience of NHS, local authority, or commercial weight management programmes in the United Kingdom, 10 academics/policy makers/commissioners working in weight management, 10 weight management staff involved in delivering a lifestyle weight management programme for adults (without significant policy involvement), and 10 primary care staff with experience of referring patients to weight management programmes (Table S2).

With regard to members of the public, in line with the sampling framework, six of 10 had experience of commercial BWMIs (60%), six of 10 were of working age (60%), and four of 10 were male (40%) (Table S2). The 10 members of the public represented nine different UK counties (six Scottish counties and three English counties).

As per the sampling framework, nine of the 10 academics/policy makers/commissioners were from England (90%), four of the 10 were academics (40%), three of the 10 were policy makers (30%), and three of the 10 were commissioners (30%) (Table S2).

Seven of the 10 primary care staff (70%) and eight of the 10 weight management staff (80%) selected were from England (Table S2).

For the second Delphi process (stage 2, instrument/definition selection), 20 academics/policy makers/commissioners and 20 weight management staff were invited to participate and included those who had successfully completed all three rounds of the stage 1 Delphi. The stage 2 Delphi involved reading papers, looking at metrics and assessing validity of instruments/questionnaires. With such a level of knowledge and expertise required, members of the public and primary care staff were not involved in this stage of the Delphi process.

Broadly in keeping with our sampling framework, 16 of the 20 stage 2 academics/policy makers/commissioners group members were from England (80%), 11 of the 20 were academics (55%), four of the 20 were policy makers (20%), and five of the 20 were commissioners (25%) (Table S3).

With regard to weight management staff, as per our sampling framework, 14 of the 20 group members were from England (70%) (Table S3).

The research team conducting the study consisted of a clinical trialist/obesity physician, a health psychologist/trialist in weight management and behaviour change, a public health researcher/specialist advisor to PHE Obesity Team, and a researcher in cardiometabolic medicine.

### 2.5 Delphi survey

**Figure 1:**
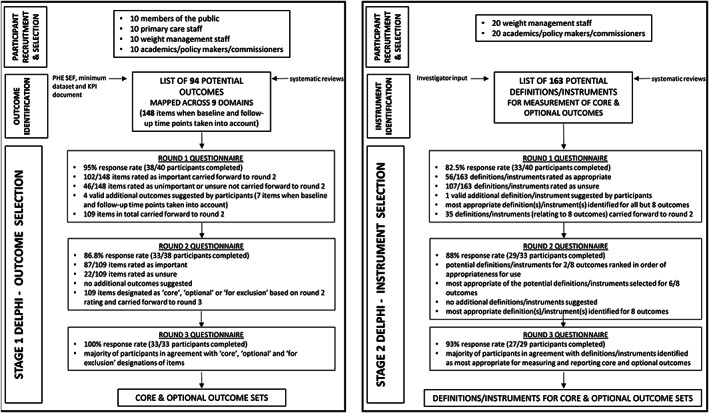
Schematic outlining the two stage Delphi study. In order to develop a core outcome set and definition/instrument set, Delphi methodology was used to gain consensus from expert groups. Two Delphis (stage 1 and stage 2) were carried out online over three rounds of questionnaires. The stage 1 Delphi focused on development of a core outcome set. The stage 2 Delphi focused on corresponding definition/instrument selection. PHE, Public Health England; SEF, standard evaluation framework; KPI, key performance indicator

Delphi methodology was used to gain consensus from the expert group. Two separate Delphi processes (stage 1 and stage 2) were conducted using an online questionnaire system (www.clinvivo.com). Each Delphi process ran over three sequential rounds with the same group of participants (Figure 1). For both the outcome selection and outcome measurement/outcome definition selection (stage 1 and stage 2) Delphi processes, those who completed a questionnaire in round 1 were eligible to participate in round 2, and those who completed round 2 were eligible to participate in round 3. In short, in order for the expert group to reach consensus, only those completing a given questionnaire were eligible to complete the subsequent questionnaire.

The stage 1, outcome selection Delphi, asked each expert to score the importance of an outcome measure for use in BWMI outcome reporting. The scale ran from 1 to 9 with 1 to 3 indicating that the outcome was unimportant, 4 to 6 indicating that it was neither unimportant nor important (“unsure”), and 7 to 9 indicating that it was important. During rounds 1 and 2, participants were also given the opportunity to suggest additional outcomes. All outcomes, excluding any rated unimportant by consensus (see Section 2.6) and including any appropriate new outcomes, were carried forward to the subsequent round (Figure 1).

During the stage 2, definition/instrument selection Delphi, experts were asked to score the appropriateness of outcome definitions and instruments for measurement of outcomes. Again, this was done using a 1 to 9 scale with 1 to 3 indicating that the definition/instrument was inappropriate, 4 to 6 indicating that it was neither appropriate nor inappropriate (“unsure”), and 7 to 9 indicating that it was appropriate. During rounds 1 and 2, participants were once more given the chance to suggest additional instruments/definitions. As for stage 1, all instruments/definitions, excluding any rated unimportant by consensus (see Section 2.6) and including any new instruments/definitions, were carried forward to the subsequent round (Figure 1).

For both stage 1 and stage 2 of the Delphi process, participant responses were summarised and fed back in subsequent rounds with participants receiving their own score and the expert group mean score for each outcome or instrument/definition.

Following round 3 of the stage 1 Delphi, consensus on the outcome set size and importance of outcomes was used to develop an outcome set. Similarly, following round 3 of the stage 2 Delphi process, a final instrument set matched to the COS was formed based on the consensus. In areas where there was no consensus, the study team adjudicated, taking account of free text comments.

### 2.6 Statistical analysis

As outlined in our published protocol^17^, the Research and Development (RAND)/University of California Los Angeles (UCLA) appropriateness method^21^ was used to assess disagreement and importance/appropriateness (and thus define consensus). This involved calculating the mean score, the median score, the inter‐percentile range (IPR, 30th and 70th), and the inter‐percentile range adjusted for symmetry (IPRAS), for each item being rated. For a given item, disagreement was indicated when the ratio of IPR to IPRAS (the disagreement index) was greater than 1.

Importance/appropriateness was assessed simply as whether the mean and/or median rating fell between 1 and 3 (unimportant/inappropriate), 4 and 6 (unsure), or 7 and 9 (important/appropriate).

At the end of each Delphi round, the mean and median ratings were determined for individual outcomes/instruments and the distribution of ratings summarised (Figure 1). Free text comments were analysed qualitatively, creating a narrative summary of responses based on the nine domains used in the questionnaire.

## 3 RESULTS

### 3.1 Outcome selection

A list of 94 outcomes for reporting from BWMIs was generated from our review of the literature and systematic review process.

The 94 outcomes were mapped across appropriate domains by consensus of three members of the research team at a face to face meeting. The domains followed section headings used in the PHE SEF^9^ and followed the weight management intervention chronological pathway (the order in which a BWMI would record outcome data as individuals progressed through the programme). There were nine domains in total (Demographics, Physical Measurements, Physical Activity, Diet, Comorbidities, Lifestyle Behaviours, Psychological Factors, Programme Specific Outcomes, and Length of Follow‐up).

### 3.2 Delphi survey—Stage 1/outcome selection

#### 3.2.1 Round 1

The final list of domains and outcomes was used to develop an online outcome selection (stage 1) questionnaire. Within the questionnaire, an explanation/definition of each outcome was provided using lay terminology as identified by the research team and approved by Clinvivo staff. With the exception of the outcomes in the Demographics, Programme Specific Outcomes, and Length of Follow‐up domains, all outcomes required measurement and reporting at both the first visit to a BWMI (baseline) and at the end of the programme/at follow‐up. This resulted in a 148‐item questionnaire with 75 outcomes for reporting at baseline and 73 outcomes at the end of the intervention. The stage 1, round 1, Delphi questionnaire can be seen, as it appeared to study participants, in Section S3. Of the 40 invited participants, 38 completed responses were received for the stage 1, round 1 Delphi questionnaire, representing a 95% response rate (100% of members of the public, academics, policy makers, commissioners, and weight management staff and 80% of primary care staff).

One hundred two of 148 outcomes were rated as important by the expert group (median rating greater than or equal to 7) with no evidence of disagreement between group members. The 102 outcomes rated as important were carried forward to the round 2 Delphi questionnaire (Table S4).

The remaining 46 outcomes were rated as being either unimportant or unsure (neither important nor unimportant) by the expert group (median rating less than or equal to 6.5, Table S4. For all but one outcome (1 month follow‐up time point, disagreement index greater than 1), expert group members were again in agreement (Table S4). Outcomes rated as unimportant or unsure were not carried forward to round 2 (Table S4).

During the round 1 questionnaire, 19 additional outcomes were suggested by expert group members (Table S5 and Section S4). The study team decided that four of the 19 suggested outcomes were unique and valid and would therefore be carried forward to the round 2 Delphi (Table S5), giving a total of 109 outcomes to be rated in this round (three of the four additional outcomes were to be rated for reporting at both first visit and end of programme).

#### 3.2.2 Round 2

The stage 1 round 2 Delphi questionnaire can be seen, as it appeared to study participants, in Section S5.

Thirty‐three of 38 completed questionnaires were received, representing an 86.8% response rate (100% of academics, policy makers, and commissioners; 90% of members of the public; and 62.5% of primary care staff).

Following analyses of round 2 questionnaires, 87 of 109 outcomes were found to have been rated as important by the expert group (median rating greater than or equal to 7). The remaining 22 outcomes were rated as unsure (median rating less than or equal to 6.5). No outcomes were rated as being unimportant, and no disagreement was evident between group members for any of the ratings (Table S4). Participants' free text comments from round 2 can be seen in Section S6. No additional outcomes were suggested during this round.

In order to enable development of an outcome set of a manageable/practical size, the study team decided that outcomes would be split into three categories (core, optional, and for exclusion) based on both their mean and median rating.

The 14 outcomes rated as most important with a mean rating greater than 7 and a median rating greater than or equal to 8 were designated as core for measurement and reporting by BWMIs (Table 1). Of these 14 outcomes, four were to be measured and reported at both first visit and at the end of the programme. An additional five outcomes (gender, ethnicity, deprivation category, learning disability, and physical disability) were then added to the core category. While these additional outcomes were rated as being important by the expert group, mean scores were not greater than 7 and/or median scores were not greater than or equal to 8. However, these outcomes are considered protected characteristics^22^ and therefore should be reported in government‐funded projects. Finally, an entirely new outcome, “formally diagnosed with a mental health condition,” was added to the core category as it was felt that its inclusion was necessary to ensure both a comprehensive COS and alignment with PHE KPI^20^. Therefore, the core set included 20 outcomes for measurement and reporting by BWMIs (Table 1).

**Table 1:** Outcomes to be considered core for measuring and reporting by behavioural weight management interventions (BWMIs)

Time Point	Outcome	Mean Panel Rating	Median Panel Rating
At baseline	Weight	8.7	9
At follow‐up	Weight	8.6	9
At follow‐up	Completion	8.5	9
At follow‐up	Attendance	8.3	9
At baseline	BMI	8.3	9
At follow‐up	BMI	8.2	9
Follow‐up time point	12 mo	8	9
At baseline	Diabetes status	7.5	8
At follow‐up	Participant satisfaction	7.5	8
Follow‐up time point	24 mo	7.5	8
At follow‐up	Cost effectiveness	7.3	8
At baseline	Age	7.2	8
At follow‐up	Diabetes Status	7.2	8
At baseline	QoL score	7.2	8
At follow‐up	QoL score	7.2	8
At follow‐up	Reason for dropout	7.2	8
At follow‐up	Adverse events/unintended consequences	7.1	8
At follow‐up	Referral to specialist services	7.1	8
At baseline	Gender^a^	6.8	8
At baseline	Deprivation category^a^	6.7	7
At baseline	Physical disability^a^	6.3	7
At baseline	Learning disability^a^	6.2	7
At baseline	Ethnicity^a^	6.1	7
At baseline	Formally diagnosed with a mental health condition^b^		

*Note*. Outcomes rated by the expert panel as being most important with a mean rating greater than 7 and a median rating greater than or equal to 8 were designated as core for measurement and reporting by BWMIs.

Abbreviations: BMI, body mass index; QoL, quality of life.

a Mean scores were not greater than 7 and/or median scores were not greater than or equal to 8, but outcomes are considered protected characteristics.

b New outcome added to ensure a comprehensive core outcome set.

Twenty‐two outcomes were rated as being reasonably important with a mean rating greater than or equal to 6.5 and less than or equal to 7.1, and a median rating less than or equal to 8. These outcomes were designated as being optional for measurement and reporting by BWMIs. Of these 22 outcomes, nine were to be measured and reported at both first visit and at the end of the programme. Of note, for four of these nine (blood pressure, cardiovascular risk, self‐esteem, and self‐confidence), the mean rating was slightly less than 6.5 for the first visit time point. However, with the corresponding end of programme/follow‐up time point meeting the rating criteria for the optional list, it was felt that these four outcomes should be included in order to ensure the follow‐up measurement was meaningful with a baseline value to compare it to. As such, the optional set included 22 outcomes for measurement and reporting by BWMIs (Table 2).

**Table 2:** Outcomes to be considered optional for measuring and reporting by behavioural weight management interventions (BWMIs)

Time Point	Outcome	Mean Panel Rating	Median Panel Rating
At follow‐up	Depression	6.9	8
At follow‐up	Repeat referrals	7.1	7
At baseline	High blood pressure	7	7
At baseline	Depression	6.9	7
At baseline	High future risk of diabetes (impaired fasting glucose, impaired glucose tolerance, raised HbA1c levels)	6.8	7
At baseline	Overall measure of comorbidity	6.8	7
At baseline	Binge eating disorder	6.8	7
At follow‐up	Representativeness	6.8	7
At follow‐up	Referral to linked services	6.8	7
Follow‐up time point	6 mo	6.8	7
At follow‐up	High blood pressure	6.7	7
At baseline	Mobility issues	6.7	7
At follow‐up	Overall measure of comorbidity	6.6	7
At follow‐up	Cardiovascular risk	6.6	7
At follow‐up	Self confidence	6.6	7
At follow‐up	Sources of referral	6.6	7
At follow‐up	Prescription of anti‐obesity medication	6.6	7
Follow‐up time point	18 mo	6.6	7
At follow‐up	High future risk of diabetes (impaired fasting glucose, impaired glucose tolerance, raised HbA1c Levels)	6.5	7
At follow‐up	Binge eating disorder	6.5	7
At baseline	High cholesterol/lipids	6.5	7
At baseline	Importance of weight loss	6.5	7
At baseline	Disordered eating	6.5	7
At follow‐up	Blood pressure	6.5	7
At follow‐up	Self esteem	6.5	7
At follow‐up	Reach	6.5	7
Follow‐up time point	3 mo	6.5	7
At baseline	Cardiovascular risk^a^	6.4	7
At baseline	Self‐confidence^a^	6.4	7
At baseline	Self‐esteem^a^	6.4	7
At baseline	Blood pressure^a^	6.2	7

*Note*. Outcomes rated by the expert panel as being reasonably important with a mean rating greater than or equal to 6.5 and less than or equal to 7.1, and a median rating less than or equal to 8 were designated as being optional for measurement and reporting by BWMIs.

Abbreviation: HbA1c, haemoglobin A1c.

a Mean scores less than 6.5 for the first visit/baseline time point but corresponding follow‐up time point scores meet rating criteria for the optional list.

The 37 outcomes rated as being least important by the expert panel (mean less than 6.5 and median less than or equal to 7) were grouped together in the “for exclusion” category. These outcomes would not be recommended for measurement and reporting by BWMIs unless participants gave a convincing argument for their inclusion during the round 3 Delphi (Table 3).

**Table 3:** Outcomes not recommended for measuring and reporting by behavioural weight management interventions (BWMIs)

Time Point	Outcome	Mean Panel Rating	Median Panel Rating
At baseline	Confidence in ability to lose weight	6.4	7
At follow‐up	Confidence in ability to lose weight	6.4	7
At follow‐up	Sedentary time	6.4	7
At follow‐up	Importance of weight loss	6.4	7
At baseline	Daily fruit and vegetable intake	6.3	7
At follow‐up	Fitness	6.3	7
At follow‐up	Mobility issues	6.3	7
At follow‐up	Disordered eating	6.3	7
At follow‐up	Anxiety	6.3	7
At baseline	Anxiety	6.2	7
At follow‐up	Waist circumference	6.2	7
At follow‐up	Leisure time physical activity	6.2	7
At follow‐up	Body image	6.2	7
At baseline	Leisure time physical activity	6.1	7
At follow‐up	Nonleisure time physical activity	6.1	7
At follow‐up	Daily fruit and vegetable intake	6	7
At baseline	Body image	6	7
At baseline	Nonleisure time physical activity	6	7
At baseline	Family history of obesity	6	7
At baseline	Smoking status	6	7
At baseline	Suicidal thoughts	6	7
At baseline	Sedentary time	5.9	7
At baseline	Fitness	5.9	7
At baseline	Weight loss history	5.9	7
At baseline	Daily alcohol consumption	5.9	7
At baseline	Asthma	5.9	7
At baseline	Other addictive behaviour	5.9	7
At follow‐up	Fat mass/body composition	5.9	7
At follow‐up	Daily calorie consumption	5.9	7
At follow‐up	Daily alcohol consumption	5.8	7
At baseline	Fat mass/body composition	5.8	7
At baseline	Daily calorie consumption	5.8	7
At follow‐up	Waist to hip ratio	5.6	7
At baseline	Waist circumference	6.2	6
At follow‐up	High cholesterol/lipids	6.1	6
At baseline	Advised To lose weight prior to routine surgery	6	6
At baseline	Osteoarthritis	5.9	6
At baseline	NAFLD	5.9	6
At follow‐up	Overall quality of sleep	5.9	6
At baseline	Overall quality of sleep	5.8	6
At baseline	Obstructive sleep apnoea	5.8	6
At baseline	Chronic back pain	5.8	6
At baseline	Other health conditions requiring a specialist diet	5.8	6
At follow‐up	Suicidal thoughts	5.7	6
At follow‐up	Obstructive sleep apnoea	5.7	6
At follow‐up	Other addictive behaviour	5.6	6
At follow‐up	Chronic back pain	5.6	6
At baseline	Chronic kidney disease	5.6	6
At baseline	Polycystic ovary syndrome (women only)	5.6	6
At baseline	Autism	5.6	6
At baseline	Personality disorders	5.6	6
At follow‐up	Daily free sugar intake	5.6	6
At follow‐up	Self‐reported reduction in clothes size	5.5	6
At follow‐up	Neck circumference	4.9	5
At baseline	Neck circumference	4.7	5

*Note*. Outcomes rated by the expert panel as being least important with a mean rating less than 6.5 and a median rating less than or equal to 7 were designated as being “for exclusion” and would therefore not be recommended for measurement and reporting by BWMIs, unless participants gave a convincing argument for their recommendation during the round 3 Delphi.

Abbreviation: NAFLD, nonalcoholic fatty liver disease.

#### 3.2.3 Round 3

The stage 1 round 3 Delphi questionnaire can be seen, as it appeared to participants, in Section S7.

Prior to commencing the questionnaire, it was explained to participants that the results of the first 2 rounds of Delphi questionnaires had allowed lists of outcomes, which would be considered core and optional for reporting by BWMIs to be made. It was explained that a list of outcomes to be excluded had also been drafted and that we would not recommend these outcomes be measured by BWMIs. Participants were informed that this would not mean that a weight management service could not measure these excluded outcomes should they wish to, but that measuring and reporting the other outcomes should be considered a higher priority.

Participants were asked to study the lists and indicate whether they agreed with the findings of the expert panel. They were advised that should they disagree with the findings, they would have the opportunity to express their disagreement and make suggestions as to any changes they felt should be made. It was made clear that if a number of participants were to express similar opinions, the lists would be altered appropriately.

The 33 expert group members who completed the round 2 questionnaire were invited to participate in the round 3 Delphi. All 33 members completed questionnaires, representing a 100% response rate for round 3. With 33/40 participants completing all three rounds of the stage 1 Delphi process, the overall response rate for stage 1 was 82.5% (100% of academics, policy makers, and commissioners; 90% of weight management staff and members of the public; and 50% of primary care staff).

Following our analyses of the completed round 3 questionnaires, 25 of 33 participants (75.8%) indicated that they were in agreement with the core and optional outcome sets. Comments from the eight participants who were not in agreement are included within Section S8. Having given these comments due consideration, the study team were of the opinion that no changes were required to the core or optional outcome sets (Tables 1 and 2) prior to the stage 2 (instrument selection) Delphi process.

As outlined in Table 1, the final list of core outcomes included “weight” (at baseline and follow‐up), “completion” (at follow‐up), “attendance” (at follow‐up), ”BMI” (at baseline and follow‐up), “diabetes status” (at baseline and follow‐up), “participant satisfaction” (at follow‐up), “cost effectiveness” (at follow‐up), ”age” (at baseline), ”Quality of Life (QoL) score” (at baseline and follow‐up), ”reason for dropout” (at follow‐up), “adverse events/unintended consequences” (at follow‐up), “referral to specialist services” (at follow‐up), “12” and “24 months” follow‐up time points, and “gender,” “deprivation category,” “physical disability,” “learning disability,” “ethnicity,” and “formally diagnosed with a mental health condition” (all at baseline).

The final list of optional outcomes included “depression” (at baseline and follow‐up), “repeat referrals” (at follow‐up), “high blood pressure” (at baseline and follow‐up), “high future risk of diabetes” (at baseline and follow‐up), “overall measure of comorbidity” (at baseline and follow‐up), “binge eating disorder” (at baseline and follow‐up), “representativeness” (at follow‐up), “referral to linked services” (at follow‐up), “mobility issues” (at baseline), “cardiovascular risk” (at baseline and follow‐up), “self‐confidence” (at baseline and follow‐up), “sources of referral” (at follow‐up), “prescription of anti‐obesity medication” (at follow‐up), “high cholesterol/lipids” (at baseline), “importance of weight loss” (at baseline), “disordered eating” (at baseline), “blood pressure” (at baseline and follow‐up), “self‐esteem” (at baseline and follow‐up), “reach” (at follow‐up), and “6,”“18,” and “3 months” follow‐up time points (Table 2).

With regard to outcomes for exclusion, 22 of 33 participants (66.7%) indicated that they were in agreement. Comments from the 11 participants who were not in agreement are included within Section S8. Again, following due consideration, the study team decided that no excluded outcomes should be retained/added to the optional outcome list prior to the stage 2 Delphi. The final list of outcomes for exclusion following the stage 1 Delphi process was, therefore, as outlined in Table 3.

### 3.3 Outcome measurement instrument selection

By reviewing the trials identified by Hartman Boyce et al^7^ and our update, definitions and instruments that could be used for measurement of the core and optional outcomes selected during the stage 1 Delphi process were listed (Table S6). Further, suitable definitions and instruments for these outcomes were added based on the study team's knowledge (Table S6).

For simplification, outcomes for which the definition or instrument was well established or where only a single possible option was available were not included in the stage 2 process, while some outcomes within the optional outcomes set were combined; “binge eating disorder” was combined with “disordered eating,” and, although slightly different concepts, “self‐esteem” and “self‐confidence” were combined. Furthermore, an outcome relating to the presentation of results was added to the core set for inclusion in the stage 2 Delphi. Due to having specific instruments for their measurement, “learning disability QoL score” and “physical disability QoL score” outcomes were also included in the core set. In addition, as it had been borderline for inclusion based on rank, required only a yes/no answer with no patient burden and was specifically mentioned in NICE guidance^1^ as a question for future research, the “repeat referrals” outcome (mean rating of 7.1 and median rating of 7) was moved from the optional to the core outcomes list (Table S6).

### 3.4 Delphi survey—Stage 2/outcome measurement instrument selection

#### 3.4.1 Round 1

The stage 2 round 1 Delphi questionnaire can be seen, as it appeared to study participants, in Section S9. Documents 1 to 8 referred to within the questionnaire were provided in parallel and included full descriptions of all instruments and, where possible, peer‐reviewed publications regarding their validity^23^, ^24^, ^25^, ^26^.

Thirty‐three of 40 completed questionnaires were received, representing an 82.5% response rate (85% of weight management staff, 82% of academics, 80% of commissioners, and 75% of policy makers).

Following analyses of completed questionnaires, 56 of 163 definitions/instruments were found to have been deemed appropriate by the expert group (median rating greater than or equal to 7) with no evidence of disagreement between expert panel members (Table 4). The remaining 107 definitions/instruments were rated as unsure (neither appropriate nor inappropriate) by the expert group (median rating less than or equal to 6.5). The expert group were in agreement (disagreement index less than 1.0) for 104 of these 107 items (Table 4).

**Table 4:** Stage 2 (instrument selection), round 1 Delphi results

Outcome Set	Outcome	Stage 2, Round 1 Questionnaire Item and Brief Description	Importance	Mean Panel Rating	Median Panel Rating	Disagreement Index (IPR:IPRAS)	Report	Retain for Stage 2, Round 2 Delphi	Discard
Core	3. Age	3.1. Mean age in years	Important	7.3	8	0.16	✓		
		3.2. % in age bands	Important	7	7	0.16	✓		
Core	4. Weight	4.1. Mean weight in kg	Important	8	9	0.13	✓		
		4.2. Mean weight change in kg	Important	7.8	9	0.29		✓	
		4.3. Mean % weight change	Important	8.1	9	0.13		✓	
		4.4. % achieving ≥3% weight loss	Important	6.5	7	0.65			✓
		4.5. % achieving ≥5% weight loss	Important	7.6	8	0.29		✓	
		4.6. % achieving ≥10% weight loss	Important	7.5	8	0.29		✓	
		4.7. % achieving ≥3 kg weight loss	Unsure	5.3	5	0.85			✓
		4.8. % achieving ≥5 kg weight loss	Unsure	5.7	5	1.04			✓
		4.9. % achieving ≥10 kg weight loss	Unsure	5.8	5	1.04			✓
Core	5. BMI	5.1. Mean BMI	Important	7.8	8	0.29	✓		
		5.2. % in BMI categories	Important	7.6	8	0.29	✓		
		5.3. Mean change in BMI	Important	7.2	8	0.29	✓		
		5.4. % achieving BMI <25	Unsure	5.2	6	0.85			✓
		5.5. % achieving BMI <30	Unsure	5.6	6	0.52			✓
Core	6. Diabetes status	6.1. % with T1DM	Unsure	5.8	5	0.52			✓
		6.2. % with T2DM	Important	7.2	7	0.49	✓		
		6.3. Mean HbA1c of those with T2DM	Unsure	6.2	6	0.65			✓
		6.4. % of those with T2DM on insulin	Unsure	5.9	6	0.65			✓
		6.5. Mean number of diabetes medications per participant with T2DM	Unsure	5.5	6	0.97			✓
		6.6. Mean change in HbA1c of those with T2DM	Important	6	7	0.52	✓		
		6.7. Mean change in % of those with T2DM on insulin	Unsure	5.5	6	0.52			✓
		6.8. Mean change in number of diabetes medications per participant with T2DM	Unsure	5.5	6	0.52			✓
Core	7. QoL score	7.1. Mean EQ‐5D‐5L scores (baseline)	Important	6.7	7	0.65	✓		
		7.2. Mean SF12 score (baseline)	Unsure	5.8	6	0.52			✓
		7.3. Mean SF36 scores (baseline)	Unsure	5.2	6	0.52			✓
		7.4. Mean IWQOL‐Lite score (baseline)	Unsure	5.7	6	0.52			✓
		7.5. Mean OWLQOL scores (baseline)	Unsure	5.4	5	0.52			✓
		7.6. Mean EQ‐5D‐5L scores (follow‐up)	Important	6.6	7	0.65	✓		
		7.7. Mean SF12 score (follow‐up)	Unsure	5.8	6	0.52			✓
		7.8. Mean SF36 scores (follow‐up)	Unsure	5.3	6	0.52			✓
		7.9. Mean IWQOL‐Lite score (follow‐up)	Unsure	5.7	6	0.52			✓
		7.10. Mean OWLQOL scores (follow‐up)	Unsure	5.5	5	0.52			✓
Core	8. Learning disability QoL score	8.1. Mean PWI‐ID score(s) 8.2. Mean score using another suitable instrument	Unsure Unsure	5.3 4.8	5 5	0.52 0.85	✓		✓
									
Core	9. Adverse events/unintended consequences	9.1. Number experiencing a worsening of pre‐existing medical condition 9.2. Number suffering severe hypoglycaemia	Important Unsure	6 5.5	7 6	0.52 0.97	✓ ✓ (merge with 9.1)		
		9.3. Number sustaining injury during physical activity session	Important	6.2	7	0.52	✓		
		9.4. Number experiencing other side effects	Unsure	5.3	6	0.97			✓
Core	10. Repeat referrals	10.1. % previously referred to service	Important	6.3	7	0.65	✓		
		10.2. % previously referred and attended ≥1 session	Important	6.3	7	0.52	✓		
Core	11. Attendance	11.1. Mean % core sessions attended	Important	7.9	8	0.13	✓		
		11.2. % attending 100% core sessions	Unsure	6.3	6	0.22			✓
		11.3. % attending ≥80% core sessions	Important	6.8	7	0.37			✓
		11.4. % attending ≥70% core sessions	Important	6.5	7	0.37			✓
		11.5. % attending ≥50% core sessions	Unsure	5.8	6	0.32			✓
Core	12. Completion	12.1. % attended 100% core sessions	Important	6.9	7	0.49		✓	
		12.2. % attended 80% core sessions	Important	6.8	7	0.49		✓	
		12.3. % attended 70% core sessions	Important	6.3	7	0.65		✓	
		12.4. % attended 50% core sessions	Unsure	5.6	6	0.32			✓
Core	13. Reason for dropout	13.1. % dropped out due to dissatisfaction with intervention (unrelated to weight loss)	Important	6.7	7	0.37	✓		
		13.2. % dropped out due to poor weight loss	Important	6.8	7	0.37	✓		
		13.3. % dropped out due to illness/hospitalisation	Important	6.8	7	0.16	✓		
		13.4. % dropped out due to pregnancy	Important	6.5	7	0.37	✓		
		13.5. % dropped out for social reason	Important	6.3	7	0.22	✓		
		13.6. % dropped out due to moving from the locale	Important	6.4	7	0.22	✓		
		13.7. % dropped out for another reason	Important	6.2	7	0.52	✓		
Core	14. Participant satisfaction	14.1. Mean adapted OEQ score 14.2 Mean NHS FFT score	Important Important	6.4 6.3	7 7	0.37 0.65		✓ ✓	
									
Core	15. Cost effectiveness	15.1. PHE Weight Management Economic Assessment Tool	Important	6	7	0.52		✓	
		15.2. Cost/kg based on mean weight loss	Unsure	5.6	6	0.52		✓	
		15.3. Cost/“success” (5% weight loss)	Important	6	7	0.52		✓	
		15.4. Cost/“success” (5 kg weight loss)	Unsure	4.5	5	0.52			✓
		15.5. Cost/“success” (3% weight loss)	Unsure	5.5	5	0.52			✓
		15.6. Cost/kg based on any change in weight data	Unsure	4.8	5	0.97			✓
Core	16. Presentation of results	16.1. Report outcomes for all attending ≥1 active weight loss session	Unsure	5.9	6	0.52		✓	
		16.2. Report outcomes for all attending >1 active weight loss session(s)/with weight loss data	Important	6.4	7	0.37		✓	
		16.3. Report outcomes for all completing programme	Important	7.3	8	0.29		✓	
Optional	17. High blood pressure	17.1. % with high blood pressure based on patient report/medication/case notes	Important	6.7	7	0.37	✓		
		17.2. % with high blood pressure based on blood pressure readings	Important	6.2	7	0.65	✓		
		17.3. Mean number blood pressure medications per participant with high blood pressure	Unsure	5.7	6	0.52			✓
		17.4. Change in mean blood pressure	Important	6.4	7	0.37	✓		
		17.5. Change in mean number blood pressure medications per participant with high blood pressure	Unsure	5.6	6	0.52			✓
Optional	18. Blood pressure	18.1. Mean systolic and diastolic blood pressure	Important	6.4	7	0.65	✓		
		18.2. % with blood pressure > 140/80 mmHg	Unsure	5.9	6	0.52			✓
		18.3. % on blood pressure medication based on self‐report/case records	Unsure	5.5	5	0.97			✓
		18.4. Change in mean systolic and diastolic blood pressure	Unsure	6.3	6	0.65	✓		
		18.5. Change in % with blood pressure > 140/80 mmHg	Unsure	5.8	6	0.52			✓
		18.6. Change in % on blood pressure medication based on self‐report/case records	Unsure	5.6	5	1.04			✓
Optional	19. CV risk	19.1. % with previous CVD	Important	6.6	7	0.22	✓		
		19.2. % with high CVD risk	Unsure	6.1	6	0.52	✓		
		19.3. % with high CV risk score (baseline)	Unsure	5.9	6	0.52	✓		
		19.4. Mean CV risk score	Unsure	5.3	6	0.97	✓		
		19.5. % on CV medications	Unsure	5.5	6	0.52			✓
		19.6. Mean number of CV medications per participant on CV medication(s)	Unsure	4.8	5	0.85			✓
		19.7. % with high CV risk score (follow‐up)	Unsure	5.6	6	0.52	✓		
		19.8. Change in mean CV risk score	Unsure	5.6	6	0.52	✓		
		19.9. Change in % on CV medications 19.10. Change in mean number of CV medications per participant on CV medication(s)	Unsure Unsure	4.9 4.8	5 5	0.85 0.97			✓ ✓
Optional	20. High cholesterol/lipids	20.1. % with high cholesterol/lipids based on self‐report/case records (baseline)	Unsure	5.8	6	0.52	✓		
		20.2. % on statin/lipid lowering medication based on self‐report/case records (baseline)	Unsure	5.5	5	0.97			✓
		20.3. Mean total cholesterol/HDL/triglycerides (baseline)	Unsure	5.6	6	0.52	✓		
		20.4. % with high cholesterol/lipids based on self‐report/case records (follow‐up)	Unsure	5.5	5	0.32			✓
		20.5. % on statin/lipid lowering medication based on self‐report/case records (follow‐up)	Unsure	5.3	5	0.97			✓
		20.6. Mean total cholesterol/HDL/triglycerides (follow‐up)	Unsure	5.4	6	0.97	✓		
Optional	21. High future risk of diabetes	21.1. % with medical record of HDR (baseline)	Unsure	6	6	0.52	✓		
		21.2. % with HDR determined by OGTT (baseline)	Unsure	4.8	5	0.85			✓
		21.3. % with HDR determined by HbA1c (baseline)	Unsure	5.9	6	0.52	✓		
		21.4. % with medical record of HDR (follow‐up)	Unsure	5.7	6	0.52	✓		
		21.5. % with HDR determined by OGTT (follow‐up)	Unsure	4.8	5	0.85			✓
		21.6. % of those with HDR at baseline who still have HDR at follow‐up as determined by OGTT	Unsure	4.7	4	0.85			✓
		21.7. % with HDR determined by HbA1c (follow‐up)	Unsure	5.9	6	0.52	✓		
		21.8. % of those with HDR at baseline who still have HDR at follow‐up as determined by HbA1c	Unsure	5.6	6	0.97	✓		
Optional	22. Overall Measure of comorbidity	22.1. mean CCI score (baseline)	Unsure	5	5	0.85		✓	
		22.2. Mean EOSS score (baseline)	Unsure	5.5	5	0.97		✓	
		22.3. Mean Chronic Disease Score (baseline)	Unsure	5	5	0.85		✓	
		22.4. Mean number dispensed medications per participant (baseline)	Unsure	5.3	5	0.52		✓	
		22.5. Mean CCI score (follow‐up)	Unsure	5	5	0.97		✓	
		22.6. Mean EOSS score (follow‐up)	Unsure	5.3	5	0.97		✓	
		22.7. Mean Chronic Disease Score (follow‐up)	Unsure	5	5	0.97		✓	
		22.8. Mean number dispensed medications per participant (follow‐up)	Unsure	5.2	6	0.97		✓	
Optional	23. Depression	23.1. % with depression based on self‐report/medication/case notes (baseline)	Important	6.2	7	0.65	✓		
		23.2. % on medication for depression (baseline)	Important	5.9	7	0.52	✓		
		23.3. Mean HADS score (baseline)	Unsure	5.7	6	0.52		✓	
		23.4. Mean PHQ9 score (baseline)	Unsure	5.9	6	0.52		✓	
		23.5. Mean Beck Depression Inventory score (baseline)	Unsure	5.5	6	0.52		✓	
		23.6. % on medication for depression (follow‐up)	Unsure	5.7	6	0.97	✓		
		23.7. % of those identified as having depression at baseline on medication for depression (follow‐up)	Unsure	5.6	6	0.52	✓		
		23.8. mean HADS score (follow‐up)	Unsure	5.6	6	0.52		✓	
		23.9. Mean PHQ9 score (follow‐up) 23.10. mean Beck Depression Inventory score (follow‐up)	Unsure Unsure	5.8 5.3	6 6	0.52 0.32		✓ ✓	
Optional	24. Self‐confidence and self‐esteem	24.1. Mean Tennesse Self‐concept Scale score (baseline)	Unsure	4.4	5	0.52			✓
		24.2. Mean Rosenberg Self‐esteem Scale score (baseline)	Unsure	5.3	5	0.97			✓
		24.3. Mean General Well‐being Schedule score (baseline)	Unsure	5.1	5	0.85			✓
		24.4. Mean ICECAP‐A score (baseline)	Unsure	4.9	5	0.85			✓
		24.5. Mean WEMWBS score (baseline)	Unsure	5.8	6	0.52	✓		
		24.6. Mean Tennesse Self‐concept Scale score (follow‐up)	Unsure	4.2	4	0.52			✓
		24.7. Mean Rosenberg Self‐esteem Scale score (follow‐up)	Unsure	5.3	5	0.97			✓
		24.8. Mean General Well‐being Schedule score (follow‐up)	Unsure	5	5	0.85			✓
		24.9. Mean ICECAP‐A score (follow‐up) 24.10. mean WEMWBS score (follow‐up)	Unsure Unsure	4.8 5.7	5 6	0.85 0.97	✓		✓
Optional	25. Importance of weight loss	25.1. Mean Dieting Readiness Scale score(s) (baseline)	Unsure	5.4	5	0.97		✓	
		25.2. Mean DIET score(s) (baseline)	Unsure	5	5	0.85		✓	
		25.3. Mean Self‐Efficacy for Eating Behaviours Scale score(s) (baseline)	Unsure	5.1	5	0.97		✓	
		25.4. Mean Dieting Readiness Scale score(s) (follow‐up)	Unsure	5.3	5	0.97		✓	
		25.5. Mean DIET score(s) (follow‐up)	Unsure	4.9	5	0.97		✓	
		25.6. Mean Self‐Efficacy for Eating Behaviours Scale score(s) (follow‐up)	Unsure	5	5	0.97		✓	
Optional	26. Disordered eating	26.1. % with disordered eating (defined as per service) (baseline)	Important	6	7	0.52	✓		
		26.2. Mean TEFQ score (baseline)	Unsure	4.8	5	0.85			✓
		26.3. Mean EDEQ score (baseline)	Unsure	5	5	0.85			✓
		26.4. Mean BES score (baseline)	Unsure	5.2	5	0.97			✓
		26.5. Mean QEWP (baseline)	Unsure	4.5	5	0.97			✓
		26.6. % with disordered eating (defined as per service) (follow‐up)	Unsure	5.8	6	0.97	✓		
		26.7. Mean TEFQ score (follow‐up)	Unsure	4.8	5	0.85			✓
		26.8. Mean EDEQ score (follow‐up)	Unsure	4.8	5	0.97			✓
		26.9. Mean BES score (follow‐up) 26.10. mean QEWP (follow‐up)	Unsure Unsure	5.2 4.5	5 5	0.97 0.97			✓ ✓
Optional	27. Reach	27.1. Age < 30	Unsure	5.6	6	0.52	✓		
		27.2. Male	Important	7.1	7	0.16	✓		
		27.3. People with T2DM	Important	7.2	7	0.16	✓		
		27.4. Other subgroups	Unsure	5.7	6	0.52	✓		
Optional	28. Representativeness	28.1. Based on age	Important	6.1	7	0.52	✓		
		28.2. Based on sex	Important	6.6	7	0.22	✓		
		28.3. Based on BMI	Important	6.7	7	0.37	✓		
		28.4. Based on deprivation category	Important	6.9	7	0.16	✓		
		28.5. Based on ethnicity	Important	6.6	7	0.37	✓		
		28.6. Based on diabetes status	Important	6.5	7	0.22	✓		
		28.7. Based on other criteria	Unsure	4.9	5	0.32	✓		
Optional	29. Prescription of anti‐obesity medication	29.1. % on any anti‐obesity medication (baseline)	Important	6.5	7	0.00	✓		
		29.2. % on specific anti‐obesity medications (baseline)	Unsure	5.7	6	0.52			✓
		29.3. % on anti‐obesity medication (follow‐up)	Important	6.2	7	0.22	✓		
		29.4. % on specific anti‐obesity medications (follow‐up)	Unsure	5.6	6	0.52			✓

*Note*. Fifty‐six of 163 definitions/instruments were rated as appropriate by the expert group (median rating greater than or equal to 7) with no disagreement between experts. One hundred seven definitions/instruments were rated as unsure (median rating less than or equal to 6.5). The expert group was in agreement (disagreement index less than 1.0) for 104 of these 107 items.

Abbreviations: BES, Binge Eating Scale; BMI, body mass index; CCI, Charlson Comorbidity Index; CV, cardiovascular; CVD, cardiovascular disease; DIET, Dieter's Inventory of Eating Temptations; EDEQ, Eating Disorder Examination Questionnaire; EOSS, Edmonton Obesity Staging System; EQ‐5D‐5L, EuroQol 5‐level EQ‐5D version; FFT, Friends and Family Test; HADS, Hospital Anxiety and Depression Scale; HbA1c, haemoglobin A1c; HDL, high‐density lipoprotein; HDR, high diabetes risk; ICECAP‐A, ICEpop CAPability measure for Adults; IPR, inter‐percentile range;IPRAS, inter‐percentile range adjusted for symmetry; IWQOL‐Lite, 31‐Item Impact of Weight on Quality of Life; NHS, National Health Service; OEQ, Outcomes and Experiences Questionnaire; OGTT, oral glucose tolerance test; OWLQOL, Obesity and Weight‐Loss Quality of Life; PHE, Public Health England; PHQ‐9, Patient Health Questionnaire‐9; PWI‐ID, Personal Wellbeing Index–Intellectual Disability; QEWP, Questionnaire on Eating and Weight PatternsQoL, quality of life; SF12, 12‐Item Short Form Health Survey; SF36, 36‐Item Short Form Health Survey; T1DM, type 1 diabetes mellitus; T2DM, type 2 diabetes mellitus; TFEQ, Three Factor Eating Questionnaire; WEMWBS, Warwick‐Edinburgh Mental Wellbeing Scale.

For all but eight outcomes, round 1 scores allowed discrimination between the definition/instrument options provided. In the majority of instances, options were selected for reporting if they were rated as important (median score greater than or equal to 7). For outcomes where none of the definition/instrument options were rated as important (learning disability QoL score, high cholesterol/lipids, high future risk of diabetes, and self‐confidence and self‐esteem), the highest scoring of the options deemed unsure were selected (Table 4). In cases where one of many definition/instrument options for an outcome received a much higher rating than the others, this option was selected for reporting and the lower scoring options were discarded despite some being rated as important (median greater than or equal to 7). An example of this can be seen for the “attendance” outcome where item 11.1, “mean % of core/mandatory sessions attended by participants“ (median value of 8 and mean value of 7.9) was selected for reporting and items 11.3, “% of participants attending greater than or equal to 80% of core/mandatory sessions,” and 11.4, “% of participants attending greater than or equal to 70% of core/mandatory sessions,” (median values of 7 and mean values of 6.8 and 6.5, respectively) were discarded. Conversely, for the “representativeness” outcome, item 28.7, “based on other criteria” was included for reporting despite being rated as unsure (median value of 5). This was because this item requested suggestions for additional measures, and one of the free text suggestions provided (geographical location) was deemed suitable for reporting. Participants' free text comments from round 1 can be seen in Section S10. Thirty‐five definitions/instruments relating to the eight outcomes listed above were carried forward to the round 2 Delphi questionnaire (Table 4).

#### 3.4.2 Round 2

The stage 2 round 2 Delphi questionnaire can be seen, as it appeared to study participants, in Section S11. Within this questionnaire, participants were required, for each of the eight included outcomes, to rank the options provided in terms of their appropriateness for use or to select a single preferred definition/instrument. As stated, 35 definitions/instruments were carried forward from the stage 2, round 1 questionnaire. However, participants were asked to consider 31 options during the stage 2 questionnaire, the result of baseline and follow‐up time points being combined where possible, and the addition of options representing a combination of definitions/instruments for a given outcome (Section S11).

The 33 expert group members who completed the stage 2, round 1 questionnaire were invited to participate in round 2 and 29/33 completed questionnaires were received, representing an 88% response rate (100% of weight management staff, 88.9% of academics, 66.7% of policy makers, and 50% of commissioners).

As shown in Section S11, participants were asked to rank seven definitions for measuring and reporting weight loss at follow‐up in order of their appropriateness for use. Results are summarised in Table 5. Based on mean and median ratings, all four potential definitions (items 3.1, 3.2, 3.3, and 3.4) were selected to be carried forward to the final definition/instrument selection Delphi (stage 2, round 3 questionnaire).

**Table 5:** Central tendency and spread of ratings for stage 2 (instrument selection), round 2 Delphi items relating to the measuring and reporting of weight loss at follow‐up

Stage 2, Round 2 Questionnaire Item	Definition/Instrument	Mean Panel Rating	*SD*	Median Panel Rating	IQR
3.1	Mean change in participants' weight in kg	4.66	2.22	5	2 to 7
3.2	Mean % weight change of participants	3.72	1.69	4	3 to 5
3.3	% of participants achieving ≥5% weight loss	3.82	1.5	4	3 to 5
3.4	% of participants achieving ≥10% weight loss	4.93	1.41	5	4 to 6
3.5	all of the above measurements (3.1 + 3.2 + 3.3 + 3.4)	3	2.31	3	1 to 5
3.6	measurements 3.2 + 3.3 (mean % weight change + % achieving ≥5% weight loss)	3.55	2.01	3	2 to 6
3.7	measurements 3.3 + 3.4 (% achieving ≥5% weight loss + % achieving ≥10% weight loss)	4.31	2.19	4	3 to 7

*Note*. Participants were asked to rank seven definitions for measuring and reporting weight loss at follow‐up in order of their appropriateness for use. Based on mean and median ratings, all 4 potential definitions (items 3.1, 3.2, 3.3, and 3.4) were selected to be carried forward to the final definition/instrument selection Delphi (stage 2, round 3).

Similarly, the expert panel ranked five options pertaining to the presentation of results at follow‐up in order of their appropriateness for use (Section S11). Results are shown in Table 6. Based on mean and median ratings, item 7.5 (combining both items 7.2 and 7.3) was selected to be carried forward to round 3.

**Table 6:** Central tendency and spread of ratings for stage 2 (instrument selection), round 2 Delphi items relating to the presentation of results at follow‐up

Stage 2, Round 2 Questionnaire Item	Definition/Instrument	Mean Panel Rating Mean	*SD*	Median Panel Rating Median	IQR
7.1	Report outcomes for all participants attending ≥1 active weight loss session(s) (does not include introductory sessions/information sessions about the service).	4.1	1.21	5	4 to 5
7.2	Report outcomes for all participants attending >1 active weight loss session(s) and therefore having weight change data (does not include introductory sessions/information sessions about the service).	3.26	1.1	3	3 to 4
7.3	Report outcomes for all participants completing the programme.	3.03	1.43	3	2 to 4
7.4	Report 7.1 + 7.3	2.62	1.18	2	2 to 4
7.5	Report 7.2 + 7.3	1.97	1.3	2	1 to 2

*Note*. Participants were asked to rank five options pertaining to the presentation of results at follow‐up in order of their appropriateness for use. Based on mean and median ratings, 2 items (items 7.2 and 7.3) were selected to be carried forward to the final definition/instrument selection Delphi (stage 2, round 3).

For the remaining six outcomes (completion, participant satisfaction, cost effectiveness, overall measure of comorbidity, depression, and importance of weight loss), experts were instructed to select the most appropriate definition/instrument for measurement and reporting from the options provided (Section S11). Selection frequency for each option was determined, and the option selected most frequently for a given outcome was then carried forward (Table 7), the exceptions being “participant satisfaction” and “overall measure of comorbidity.” For the former, experts' comments and scores indicated that neither of the suggested instruments (questionnaires) was ideal. Therefore, it was decided that both instrument options would be retained for round 3, but the expert panel would be informed that alternative methods to measure this outcome could be used. In the case of “overall measure of comorbidity,” the majority of experts indicated that they had insufficient knowledge of the instruments and were therefore unable to select which would be most appropriate for use. Consequently, the most frequently selected of the remaining options, mean Edmonton Obesity Scale Score (EOSS) score, was selected to be carried forward to round 3.

**Table 7:** Selection frequencies for remaining stage 2 (instrument selection), round 2 Delphi items

Stage 2, Round 2 Questionnaire Item	Outcome	Definition/Instrument	Selection Frequency	Selection Percentage (%)	Retain for Round 3 Delphi
4.1	Completion	% of participants who attended 100% of possible/core/mandatory sessions	2	7	
4.1	Completion	% of participants who attended 80% of possible/core/mandatory sessions	15	52	✓
4.1	Completion	% of participants who attended 70% of possible/core/mandatory sessions	12	41	
5.1	Participant satisfaction	Mean OEQ score adapted to suit weight management services	13	45	✓^a^
5.1	Participant satisfaction	Mean NHS FFT score	16	55	✓^a^
6.1	Cost effectiveness	The PHE Weight Management Economic Assessment Tool	18	62	✓
6.1	Cost effectiveness	Cost/kg (based on mean weight loss)	6	21	
6.1	Cost effectiveness	Cost per success with success being 5% weight loss	5	17	
8.1	Overall measure of comorbidity	Mean CCI score	2	7	
8.1	Overall measure of comorbidity	Mean EOSS score	7	24	✓^b^
8.1	Overall measure of comorbidity	Mean Chronic Disease Score	3	10	
8.1	Overall measure of comorbidity	Mean number of dispensed medications per participant	1	3	
8.1	Overall measure of comorbidity	I have insufficient knowledge of the instruments and am therefore unable to select one.	16	55	
9.1	Depression	Mean HADS questionnaire score of participants	10	34	
9.1	Depression	Mean PHQ9 questionnaire score of participants	12	41	✓
9.1	Depression	Mean Beck Depression Inventory score of participants	7	24	
10.1	Importance of weight loss	Mean Dieting Readiness Scale score(s)	15	52	✓
10.1	Importance of weight loss	Mean DIET score(s)	8	28	
10.1	Importance of weight loss	Mean Self‐Efficacy for Eating Behaviours Scale score(s)	6	21	

*Note*. Participants were instructed to select the most appropriate definition/instrument for measurement and reporting from the options provided for each outcome. Selection frequency for each option was determined and the option selected most frequently retained for the stage 2, round 3 Delphi.

Abbreviations: SD, standard deviation; IQR, interquartile range; OEQ, Outcomes and Experiences Questionnaire; NHS, National Health Service; FFT, Friends and Family Test; PHE, Public Health England; CCI, Charlson Comorbidity Index; EOSS, Edmonton Obesity Staging System; HADS, Hospital Anxiety and Depression Scale; PHQ‐9, Patient Health Questionnaire‐9; DIET, Dieter's Inventory of Eating Temptations.

a Participants' comments and scores indicated that neither of the suggested instruments was ideal. Therefore, no instrument was selected. These two options will be given as suggestions but other methods could be used.

b The majority of participants indicated that they had insufficient knowledge of the instruments and were therefore unable to select which would be most appropriate for use. Consequently, the most frequently selected of the remaining options, mean EOSS score, was retained for the stage 2, round 3.

Participants' free text comments from round 2 can be seen in Section S12.

#### 3.4.3 Round 3

Experts were asked to study the final list of selected definitions/instruments and indicate whether they were in agreement with the findings of the expert panel. If participants disagreed with the findings they had the opportunity to express this disagreement and make suggestions as to any changes they felt should be made. It was made clear that should a number of experts express similar opinions, instruments/measurements would be altered appropriately. The stage 2, round 3, questionnaire is included, as it appeared to participants, as Section S13.

The 29 expert group members who completed the stage 2, round 2, questionnaire were invited to participate in the round 3 Delphi process, and 27/29 completed round 3 questionnaires were received, representing a 93% response rate for this round (100% of weight management staff, 100% of academics, 50% of policy makers, and 50% of commissioners). With 27/40 participants completing all three rounds of the stage 2 Delphi process, the overall response rate for stage 2 was 67.5% (85% of weight management staff, 72.7% of academics, 25% of policy makers, and 20% of commissioners).

Following analyses of round 3 questionnaires, results revealed that 19/27 experts (70%) approved the results as presented and 8/27 experts (30%) did not. With regard to expert panel subgroups, 7/8 academics (88%) approved the results as presented and 1/8 (13%) did not. The participant who identified as a commissioner accepted the results as presented, as did the participant who identified as a policy maker. Of the weight management staff, 10/17 (59%) agreed with the results as presented and 7/17 (41%) did not. Therefore, the most disagreement and, consequently, free text comments came from weight management staff who tended to pre‐empt their responses by stating that they partially accepted the results rather than rejecting them outright (Section S14). Comments suggested that the main concern was related to measures of diabetes status with participants questioning whether there was capacity in services to perform the necessary medical tests, who would fund these tests and whether performing them would place an unreasonable burden on weight management staff (Section S14). However, with the vast majority of the expert group in agreement with the results and free text comments of those not in agreement failing to provide a convincing argument for alteration of the final definition/instrument list, our core and optional outcome and definition/instrument sets were finalised and are included as Table 8. As shown, “outcomes” within both sets were designated as being either process outcomes, outcomes, or guidance for presentation of results (Table 8).

**Table 8:** Core and optional outcome and definition/instrument sets

Core Outcome Set			
Process Outcomes			
Item	Definition	Instrument/Measurement/Presentation to be Used/Reported (Baseline)	Instrument/Measurement/ Presentation to be Used/Reported (Follow‐up)
Age	How old participants are/the age (in years) of participants	Mean age of participants in years % of participants in age bands (16‐24, 25‐34 , 35‐44, 45‐54, 55‐64, 65‐74, 75+ y)	
Gender^a^	How participants identify themselves with regard to being male, female, or nonbinary/third gender	% of male, female, or other participants	
Ethnicity^a^	The social group with common national and cultural tradition that participants identify as belonging to, eg, white/white British, Asian/Asian British, black/African/Caribbean/black British	% of participants identifying as being white, black, Asian, or Minority Ethnicities	
Deprivation category^a^	A measure of the level of poverty in the area in which the participant lives	Scotland—% of participants in each Scottish Index of Mass Deprivation (SIMD) quintile England—% of participants in each English Index of Mass Deprivation (EIMD) quintile Wales—% of participants in each Welsh Index of Mass Deprivation (WIMD) quintile Northern Ireland—% of participants in each Northern Ireland Multiple Deprivation Measure (NIMDM) decile	
Physical disability^a^	Whether participants have a recognised physical disadvantage or disability		
Learning disability^a^	Whether participants have a recognised mental/cognitive disadvantage or disability		
Formally diagnosed with a mental health condition^a^	Whether participants have a current mental health condition as diagnosed by a GP or health professional	% of participants answering yes	
Referral to specialist services (real world services only)	Whether a participant has been referred to a specialist management service (tier 3 or 4) by a GP or tier 2 weight management service after failing to lose the required amount of weight via a lifestyle weight management programme or due to a condition needing specialist input.		% of participants
Repeat referrals (real world evaluations only)	Whether a participant has been referred to the weight management service on more than one occasion.		% of participants previously referred to the service, not necessarily having attended any sessions) % of participants answering yes, having previously attended at least 1 weight management session
Attendance	How many people attended the weight management service		Mean % of core/mandatory sessions attended by participants
Completion	How many people finished the weight management programme		% of participants who attended 80% of possible/core/mandatory sessions
Reason for dropout	Why those participants who did not complete the programme failed to do so.		% of participants who dropped out due to: Dissatisfaction with the intervention (unrelated to weight loss) Poor weight loss Illness/ hospitalisation Pregnancy Change in personal circumstances/social reason Moving from the geographical area Any other reason Unknown reason
Core Outcome Set			
Outcomes			
Item	Definition	Instrument/Measurement/Presentation to be Used/Reported (Baseline)	Instrument/Measurement/ Presentation to be Used/Reported (Follow‐up)
Weight	The measurement of how heavy a participant is in kilograms (kg) or stones and pounds	Mean weight of participants in kg	Mean change in participants' weight in kg Mean % weight change of participants % of participants achieving ≥5% weight loss % of participants achieving ≥10% weight loss
Body mass index (BMI)	An approximate measure of whether a participant is overweight or underweight, calculated by dividing their weight in kilograms by the square of their height in metres	Mean BMI of participants % of participants in BMI categories <25, 25‐29.9, 30‐34.9, 35‐39.9, 40‐49.9, 50‐59.9, ≥60	mean change in participants' BMI
Diabetes status	Whether a participant has diabetes, a condition, which occurs when the body does not produce enough insulin to function properly, or the body's cells do not react to insulin. This means glucose stays in the blood and isn't used as fuel for energy. Type 2 diabetes is often associated with obesity and an increased risk of developing cardiovascular disease.	% of participants with type 2 diabetes mellitus (based on self‐report, case record, or blood test)	Mean change in HbA1c levels of those participants with T2DM
Quality of life (QoL) score	A measure of the general well‐being of participants.	Mean EQ‐5D‐5L scores of participants	Mean change in EQ‐5D‐5L scores of participants
Learning disability QoL score	A measure of the general well‐being of participants with a learning disability.	Mean Personal Wellbeing Index‐Intellectual Disability (PWI‐ID) score(s) of participants	Mean change in PWI‐ID score(s) of participants
Adverse events/unintended consequences	Whether participants suffered any unfortunate side effects as a result of attending the weight loss service.		Number of participants experiencing a worsening of a pre‐existing medical condition, such as An undiagnosed eating disorder Other pre‐existing medical conditions Number of participants sustaining an injury during a physical activity session run by the weight management service
Participant satisfaction	How happy/satisfied participants were with the weight loss service. **In this instance, the weight management service should select the questionnaire/method they feel is most appropriate for their use.**		Comments and scores indicate that neither of the suggested instruments for measuring patient satisfaction is ideal. Therefore, it is proposed that no instrument is selected. The two options below will be given as suggestions but other methods could be used. Mean Outcomes and Experiences Questionnaire (OEQ) score adapted to suit weight management services Mean NHS Friends and Family Test (FFT) score
Cost effectiveness	The value for money of the weight management service in terms of long term economic benefits to the NHS.		The Public Health England Weight Management Economic Assessment Tool: http://webarchive.nationalarchives.gov.uk/20170110165804/http://www.noo.org.uk/visualisation/economic_asessment_tool
Guidance for Presentation of Results (Core Outcome Set)			
Item	Definition	Presentation to be Used	
Presentation of results	Which participants' outcomes to include in reporting	Report outcomes for all participants attending >1 active weight loss session(s) and therefore having weight change data (does not include introductory sessions/information sessions about the service) Report outcomes for all participants completing the programme	
12‐mo follow‐up	Reporting outcomes 12 mo after starting the weight loss programme		
24‐mo follow‐up	Reporting outcomes 24 mo after starting the weight loss programme		
Missing data	How to deal with participants with missing weight data (usually because they have dropped out of the programme)	baseline observation carried forward (BOCF) and last observation carried forward (LOCF) for data at <12 months BOCF for data at ≥12 months	
Optional Outcome Set			
Process Outcomes			
Item	Definition	Instrument/Measurement/Presentation to be Used/Reported (Baseline)	Instrument/Measurement/Presentation to be Used/Reported (Follow‐up)
Reach (% eligible population who are referred to/take up weight management service)	The percentage of the eligible population (people who are overweight or obese within that particular geographical area) referred to the weight management service.		For a specific population subgroup of concern, what % of that population has been referred to/ attended the weight management service. Local data (eg, Quality and Outcomes Framework) can be used to obtain prevalence rates. Population subgroups of interest: Age <30 Male People with T2DM Other subgroups
Representativeness (how similar the people attending the service are to the local eligible population)	How representative of the entire eligible population (people with body mass in the overweight or obese range within that particular geographical area) the people attending the weight management service are.		Based on age of participants Based on sex of participants Based on BMI of participants Based on deprivation category of participants Based on ethnicity of participants Based on diabetes status of participants Based on the geographical spread of the home addresses of participants
Referral to linked services	The number of participants referred to services linked to weight management services		% of participants referred to smoking cessation services, mental health services, alcohol services etc
Sources of referral	Where participants received their referral to the weight management service	% of participants receiving their referral from each possible source dependent on service, eg, from primary care, from secondary care, self‐referral, from allied health professionals, from pharmacy, from tier 3 weight management services, from tier 4 weight management services	
Mobility issues	Whether participants are unable to move with ease and without restriction. Being overweight has been associated with restricted mobility.	% of participants who have difficulty accessing certain weight loss service venues and have impaired ability to exercise	
Optional Outcome Set			
Outcomes			
High blood pressure	Whether a participant has high blood pressure. High blood pressure increases the risk of developing cardiovascular disease.	% of participants with high blood pressure based on patient report/medication/case notes % of participants with high blood pressure based on blood pressure readings	Change in % of individuals with blood pressure above current recommended treatment thresholds (ie, normotensive or adequately treated)
Blood pressure	The pressure of blood in the arteries, the vessels that carry blood from the heart to the rest of the body. A certain amount of pressure is required to get the blood around the body but consistently high blood pressure increases the risk of cardiovascular disease.	Mean systolic and diastolic blood pressure of participants	Change in mean systolic and diastolic blood pressure of participants
Cardiovascular risk	A measure of how likely participants are to develop cardiovascular disease, including heart disease and stroke	% of participants with previous cardiovascular disease (CVD), including myocardial infarction, stroke, transient ischaemic attack (TIA), angina, and peripheral vascular disease % of participants with high CVD risk (previous CVD or a high cardiovascular risk score—N.B. information on blood pressure and lipids would be required to calculate the risk score) % of participants with a high cardiovascular risk score (primary prevention/not those with previous cardiovascular disease) mean CVD risk score of participants (primary prevention/not those with previous cardiovascular disease)	% of participants with a high cardiovascular risk score (primary prevention/not those with previous cardiovascular disease) change in mean cardiovascular risk score of participants (primary prevention/not those with previous cardiovascular disease)
High cholesterol/ lipids	A measure of whether a participant has an abnormal amount of fat and/or cholesterol, known as lipids, in their blood (also called dyslipidaemia). Being overweight can increase the likelihood of developing dyslipidaemia. Dyslipidaemia is associated with increased risk of developing cardiovascular disease.	% of participants with high cholesterol/lipids based on self‐report /case records Mean total cholesterol/ high density lipoprotein/ triglycerides of participants as obtained via blood test	Mean change in total cholesterol/ high density lipoprotein/ triglycerides of participants as obtained via blood test
High future risk of diabetes (impaired fasting glucose, impaired glucose tolerance, raised HbA1c levels, previous gestational diabetes)	Whether measures of the amount of glucose in a participant's blood suggests he/she is likely to develop type 2 diabetes in the future.	% of participants with a medical record of high diabetes risk (HDR) as determined by measuring HbA1c/fasting glucose/Oral Glucose Tolerance Test (OGTT) (either measured during intervention or in medical records)	% of all participants with HDR as determined by measuring HbA1c/fasting glucose/OGTT (either measured during intervention or in medical records) % of those participants identified as having HDR at baseline who still have HDR (as determined by measuring HbA1c/fasting glucose/OGTT) , normoglycemia or type 2 diabetes
Overall measure of comorbidity	Measure of the presence of additional diseases or disorders co‐occurring with obesity/being overweight	Mean Edmonton Obesity Staging System (EOSS) score	Mean change in EOSS score
Depression	Whether a participant suffers from a mental illness characterised by a profound and persistent feeling of sadness or despair and/or a loss of interest in things that once were pleasurable.	% of participants with depression based on patient report/medication/case notes % of participants on medication for depression Mean Patient Health Questionnaire‐9 (PHQ‐9) score of participants	Change in % of all participants on medication for depression Change in % of those patients identified as having depression at baseline on medication for depression Mean change in PHQ‐9 questionnaire score of participants
Self‐confidence and self‐esteem	How participants feel about their own abilities and worth	Mean Warwick‐Edinburgh Mental Well‐being Scale (WEMWBS) score	Mean change in WEMWBS score
Importance of weight loss	How important participants feel it is for them to lose weight	Mean Dieting Readiness Scale score(s)	
Disordered eating	Whether participants have disturbed and unhealthy eating patterns that can include restrictive dieting, compulsive eating or skipping meals. Disordered eating can include behaviours, which reflect many but not all of the symptoms of feeding and eating disorders such as anorexia nervosa, bulimia nervosa, and binge eating disorder.	% of participants with disordered eating (defined as per service)	Change in % of participants with disordered eating (defined as per service)
Prescription of anti‐obesity medication	The number of participants taking drugs to help reduce or control their weight	% of participants on any anti‐obesity medication (total and by class/medication)	Change in % of participants on anti‐obesity medication (total and by class/medication)
Guidance For Presentation of Results (Optional Outcome Set)			
3‐mo follow‐up	Reporting outcomes 3 mo after starting the weight loss programme		
6‐mo follow‐up	Reporting outcomes 6 mo after starting of the weight loss programme		
18‐mo follow‐up	Reporting outcomes 18 mo after starting the weight loss programme		

*Note*. The expert group agreed on a final core outcome and corresponding definition/instrument set consisting of 24 items. Twelve of these items were designated as processes, eight were designated as outcomes, and four were designated as guidance for presentation of results. Experts agreed on an optional outcome set consisting of 19 items; five processes, 11 outcomes, and three items relating to presentation of results.

a These items are considered “protected characteristics” and therefore, in keeping with government guidelines, have been included in our core outcome set. These items are more relevant for real world services which are required to report such items to higher authorities. As such, these items are only core or mandatory for reporting when required in real life.

## 4 DISCUSSION

A COS is an agreed minimum set of outcomes for measuring and reporting for a specific area of health. COSs have been developed across a range of health areas, including bariatric and metabolic surgery^27^. While a recent study obtained expert panel consensus on recommendations for standard baseline assessment in medical obesity management clinics^28^, to our knowledge, the study described herein is the first of its kind to develop a COS and corresponding definition/instrument set for BWMIs for adults with overweight and obesity. This is much needed in order to standardise reporting which, in turn, will lead to a better evidence base and improvements in weight management provision. Indeed, within the United Kingdom, PHE and Health Scotland have agreed to use this work to inform evaluation plans for adult BWMIs.

A wide range of sources, including the research literature and guideline and policy documents, were used to generate lists of potential outcomes and definitions/instruments. Consensus as to which of these should be included in the final outcome sets was then determined by a group of individuals with wide‐ranging expertise in behavioural weight management. This was achieved by means of the internationally recognised Delphi process. Experts included members of the public with experience of BWMIs, academics/commissioners/policy makers working in weight management, weight management staff and primary care staff (referrers). There is no published agreement on the optimal size of an expert group^29^; pragmatism is required while ensuring a range of opinions is garnered. For this study, experts were selected according to our sampling framework to ensure they were representative of the United Kingdom as a whole, and the online nature of the Delphi process ensured that opinions expressed by members of the public were given equal weighting to those expressed by professionals. However, throughout the majority of the Delphi process, experts from each of the four groups were observed to be in agreement as to the importance of outcomes for reporting from BWMIs and the appropriateness of definitions/instruments for their measurement. In addition, retention rates for our experts were high throughout the Delphi process with 82.5% completing stage 1 (outcome selection) and 67.5% completing stage 2 (instrument selection). These high retention rates can be attributed to the nature of our recruitment and selection processes. In order to select a panel based on our sampling framework, potential experts were asked to provide information on geographical location etc. Those responding appropriately in a timely manner demonstrated their willingness to participate and their commitment to the process and were therefore considered for Delphi expert panel selection. Those failing to respond to our requests were deemed unlikely to fully engage with the Delphi process and were not included in the selection process.

Experts agreed on a final core outcome and corresponding definition/instrument set consisting of 24 items, which were designated as either processes, outcomes, or guidance for presentation of results. As we may have expected, weight, BMI, attendance, completion, and cost effectiveness featured in the final COS and follow‐up time points of 12 and 24 months were stipulated. Experts also agreed that an additional optional COS was necessary. This included 19 items, again designated as either processes, outcomes, or guidance for presentation of results, which BWMIs could report should they wish to do so. Both the core and optional outcome sets were observed to include outcomes relevant to patients, clinicians, and commissioners/policy makers, reflecting the composition of our expert group.

While the vast majority of experts were in agreement with the final outcome and corresponding definition/instrument sets, some issues were raised by weight management staff with regard to the feasibility of the outcomes. With these concerns in mind, it should be noted that the measurement of each outcome is not considered mandatory for every patient/participant; the outcome sets are merely intended to serve as a guide for planned evaluations. A lack of funding and requirement for evaluation is a key issue for real‐world services. The majority of outcomes in the COS are generally measured during routine care, but it is recognised that certain outcomes will prove more challenging for weight management staff, an example being the determination of haemoglobin A1c (HbA1c) levels if linking to routinely measured test results is not possible. In addition, information on longer term outcomes (at 12 and 24 months) is likely to be difficult to obtain given the relatively short duration of the majority of BWMIs. Furthermore, those participants who regain weight are less likely to provide weight details or return to be weighed at a later stage. As such, research is needed in order to improve linkage to health records and to determine how best to persuade patients/participants to engage with longer term outcomes^1^, perhaps by digital means, such as blue tooth scales or mobile apps. There is also a need for commissioners to consider the benefits of evaluation at the point of commissioning a service and ensuring that the service is funded sufficiently in order to gain meaningful insights^30^.

This study was, of course, restricted to the United Kingdom. This is due to BWMIs and their settings within health services being fairly country‐specific. For example, in France and the Netherlands, there is no health insurance funding of BWMIs, and in the United States of America (USA), obesity services are tertiary, combining behavioural programmes with medication and bariatric surgery. Instruments can also be country‐specific due to differences in language and health economic models, for example. In addition, “international” studies are often tokenistic, including only a small percentage of participants from outside the country in which the study is set. Within the “international” BARIACT study for example, the vast majority of professionals (95.2%) and patients (95.6%) participating were from the United Kingdom^27^. Our preference was to develop a COS with a balanced stakeholder group using a sampling framework to ensure wide representation; to do this on a truly international scale would be impossible. Consequently, if used in an international context for trials or real world services, our core outcome and definition/instrument set may require further adaptation. Therefore, the next step may be to undertake international validation of the COS. This could involve consensus meetings with professionals and patients in other countries.

In conclusion, this study has used internationally recognised methodology to develop a COS for BWMIs. Its widespread adoption by both clinical trialists and weight management programmes will improve the quality of data from research studies and real‐life services, thus improving the evidence base and weight management provision.

## FUNDING INFORMATION

This work was supported by the CSO of the Scottish Government Health Department, grant reference number CGA/17/08. SAS was supported by a Medical Research Council (MRC) Strategic Award (MC‐PC‐13027, MC_UU_12017_14, and SPHSU14).

## CONFLICT OF INTERESTS

J.L. leads a joint working project between University of Glasgow, National Health Service (NHS) Greater Glasgow and Clyde, MSD. and Astra Zeneca. The project also involved an educational grant from Janssen. J.L. received funding to attend a conference from Novo Nordisk. L.J.E. has a part time secondment with P.H.E.. L.J.E. has also received funding from North Yorkshire County Council, PHE, Sport England, National Prevention Research Initiative (NPRI), and Chief Scientist Office (CSO) Scotland in the last 3 years.

## AUTHOR CONTRIBUTIONS

R.M.M. and J.L. drafted the manuscript. L.J.E. and S.A.S. critically reviewed the manuscript. R.M.M. and J.L. finalised the manuscript.

## Supporting information

Data S1. Supporting Information
