# Supplementary material for: Core outcome set for behavioural weight management interventions for adults with overweight and obesity: Standardised reporting of lifestyle weight management interventions to aid evaluation (STAR‐LITE)
Source: Obes Rev. 2019 Nov 22;21(2):e12961. doi: 10.1111/obr.12961 (PMC7050499; doi:10.1111/obr.12961)
Supplement: Supplementary file 1 — Data S1. Supporting Information [file OBR-21-e12961-s001.pdf]

Core Outcome Set for Behavioural Weight Management Interventions for Adults with Overweight and Obesity: STAndardised Reporting of Lifestyle Weight Management InTerventions to Aid Evaluation (STAR-LITE)

Ruth M. Mackenzie[1], Louisa J. Ells[2], Sharon Anne Simpson[3], Jennifer Logue[4]

[1]Dr Ruth M. Mackenzie

Institute of Cardiovascular and Medical Sciences

University of Glasgow

Ruth.Mackenzie@glasgow.ac.uk

[2]Professor Louisa J. Ells

School of Health & Social Care

Teesside University

L.Ells@tees.ac.uk

[3]Professor Sharon Anne Simpson

Institute of Health and Wellbeing

University of Glasgow

Sharon.Simpson@glasgow.ac.uk

[4]Dr Jennifer Logue

Institute of Cardiovascular and Medical Sciences

University of Glasgow

Jennifer.Logue@glasgow.ac.uk

Supporting Information 1. Inclusion criteria for Hartmann-Boyce *et al.*[1] and updated systematic review.

Inclusion and exclusion criteria

Criteria for including and excluding studies were similar to those used in the Loveman[2] 2011 report, with two key changes: the NICE review did not include behavioural weight management programmes (BWMPs) that involved medications for obesity of any type, unless their use was not part of the BWMP and was comparable in both intervention and control groups. In addition, the NICE review included studies with 12 month follow-up or longer (Loveman[2] required a minimum of 18 months follow-up). The revised inclusion criteria are listed below.

Population

- Adults (≥ 18 years) classified as overweight or obese, i.e. people with a BMI of ≥ 25 kg/m2 and ≥ 30 kg/m^2, respectively, or a BMI of ≥ 23 kg/m^2 in Asian populations. Where overweight or obesity was not an inclusion criterion, they included studies where greater than 80% of each arm was overweight/obese (note, this differs from Loveman[2], who did not specify guidelines for dealing with such studies).

- Studies in children, pregnant women, and people with eating disorders were not included, nor were studies specifically in people with a pre-existing medical condition such as diabetes, heart failure, uncontrolled hypertension or angina. However, they did include studies in specific at-risk populations, most notably studies aiming for diabetes prevention, conducted in populations with elevated fasting glucose or impaired glucose tolerance (but without diabetes mellitus). This also differs from Loveman's approach which excluded diabetes prevention studies[2].

Intervention

• Structured, sustained multi-component weight management programmes (i.e. the intervention had to be a combination of diet and physical activity with a behaviour change strategy to influence lifestyle).

• Components of the programme had to be clearly specified (i.e. details provided of the diet, behavioural definition, and exercise components; see below). • Programmes that included a long-term follow-up of more than 12 months. Unlike Loveman[2], who required follow-up of 18 months or longer.

• The programme was delivered in the health sector, in the community or commercially.

• Multi-component programmes that involved the use of any surgery or medication, over the counter or otherwise, were excluded.

• Interventions incorporating other lifestyle changes such as efforts at smoking cessation or reduction of alcohol intake were not included. Unlike Loveman[2], they excluded studies which only looked at a specific component of an intervention so that comparator interventions differed only by a single element, for example presence or absence of self-monitoring, or differences in dietary composition.

Comparators

The comparator had to fit into one of the following groups

1. No intervention at all or leaflet/s only.

2. Discussion/advice/counselling in one-off session +/-leaflet .

3. Seeing someone more than once for discussion of something other than weight loss.

4. Seeing someone more than once for weight management, person untrained +/- leaflets.

This is in contrast to Loveman[2], where the control condition was normal practice (as defined by the study).

Outcomes

• Studies were required to include a measure of weight loss.

Types of studies

• Randomized controlled trials (RCTs) only.

• Studies published as abstracts or conference presentations were only included if sufficient details were presented to allow an appraisal of the methodology and the assessment of results to be undertaken.

Location

• Undertaken in any setting (i.e. community, commercial, primary care, online).

• Studies conducted in Organisation for Economic Co-operation and Development (OECD) countries were considered for inclusion. In the instance that a study was conducted in an OECD country but the reviewers and advisory panel judged that the intervention would not be feasible for implementation in the UK, the reviewers consulted with CPHE regarding its inclusion.

• Studies conducted in non OECD countries were excluded.

Specification of components of intervention

Loveman *et al.*[2] required that, in order for a study to be included, at least two items under each of the below components (diet, exercise, and behaviour modification) had to be specified.

Diet

- type of diet
- calories
- proportion of diet (e.g. proportion of diet made up of fats, protein, carbohydrate)
- monitoring

Exercise

- mode
- type
- frequency/length sessions
- delivered by
- level of supervision
- monitoring

Behaviour modification

- mode
- type
- content
- frequency/length sessions
- delivered by.

They required these same criteria, but modified them as follows. Where studies were multicomponent but the study report did not meet the above criteria, they followed the approach below:

- If the study reported on the effectiveness of a weight loss programme , they searched online for details of the weight loss programme and used these to classify the study components. Where insufficient details were available online, they contacted the programme directly, specifying that a response would be needed by 20 December 2012.
- If the details of the programme were not available online, they emailed study authors with a template email asking them to provide any details they had on the above elements, specifying that a response was needed by 20 December 2012.
- Where authors did not respond by the deadline specified, provided insufficient information, or where they could not find a current e-mail address, the study was excluded, with the reason for exclusion clearly identified.

• For consistency, they followed this same approach for studies that Loveman[2] had listed as excluded on the basis of insufficient intervention detail.

Updated Hartman-Boyce *et al.*[1] Review

Additional outcomes were identified by updating the Hartmann-Boyce[1] systematic review, using the same inclusion criteria but extending search dates so that studies from 1/11/2012 until 30/09/2017 were included. This identified 31 additional papers: Anderson *et al*.(2014)[3], Arden-Close *et al.*(2017)[4], Batsis *et al.*(2017)[5], Beavers *et al.*(2013)[6], Bennett *et al.* (2013)[7], Bhopal *et al.* (2014)[8], Burgess *et al.* (2017)[9], Compernolle *et al.* (2014)[10], Danielsen et al. (2013)[11], de Vos et al. (2016)[12], Gray et al. (2013)[13], Greaves et al. (2015)[14], Gudzune et al. (2015)[15], Hassan et al. (2016)[16], Huseinovic et al. (2016)[17], Johns et al. (2014)[18], Johnston et al. (2014)[19], Jull et al. (2014)[20], Mason et al. (2014)[21], Moreno et al. (2014)[22], Neville et al. (2014)[23], Partridge et al. (2015)[24], Pekkarinen et al. (2015)[25], Phelan et al. (2017)[26], Poulsen et al. (2015)[27], Runhaar et al. (2015)[28], Schwingshackl et al. (2014)[29], Unick et al. (2015)[30], Wadden et al. (2014)[31], Weerasekara et al. (2016)[32], Williams et al. (2015)[33].

References

(1) Hartmann-Boyce J, Johns D, Aveyard P, Lewis A, Jebb S, Phillips D et al. Managing overweight and obese adults: The clinical effectiveness of long-term weight management schemes for adults. 2013.

(2) Loveman E, Frampton GK, Shepherd J, Picot J, Cooper K, Bryant J et al. The clinical effectiveness and cost-effectiveness of long-term weight management schemes for adults: a systematic review. *Health Technol Assess* 2011; 15(2):1-182.

(3) Anderson AS, Craigie AM, Caswell S, Treweek S, Stead M, Macleod M et al. The impact of a bodyweight and physical activity intervention (BeWEL) initiated through a national colorectal cancer screening programme: randomised controlled trial. *BMJ* 2014; 348:g1823.

(4) Arden-Close E, McGrath N. Health behaviour change interventions for couples: A systematic review. *Br J Health Psychol* 2017; 22(2):215-237.

(5) Batsis JA, Gill LE, Masutani RK, Adachi-Mejia AM, Blunt HB, Bagley PJ et al. Weight Loss Interventions in Older Adults with Obesity: A Systematic Review of Randomized Controlled Trials Since 2005. *J Am Geriatr Soc* 2017; 65(2):257-268.

(6) Beavers KM, Ambrosius WT, Nicklas BJ, Rejeski WJ. Independent and combined effects of physical activity and weight loss on inflammatory biomarkers in overweight and obese older adults. *J Am Geriatr Soc* 2013; 61(7):1089-1094.

(7) Bennett GG, Foley P, Levine E, Whiteley J, Askew S, Steinberg DM et al. Behavioral treatment for weight gain prevention among black women in primary care practice: a randomized clinical trial. *JAMA Intern Med* 2013; 173(19):1770-1777.

(8) Bhopal RS, Douglas A, Wallia S, Forbes JF, Lean ME, Gill JM et al. Effect of a lifestyle intervention on weight change in south Asian individuals in the UK at high risk of type 2 diabetes: a family-cluster randomised controlled trial. *Lancet Diabetes Endocrinol* 2014; 2(3):218-227.

(9) Burgess E, Hassmen P, Welvaert M, Pumpa KL. Behavioural treatment strategies improve adherence to lifestyle intervention programmes in adults with obesity: a systematic review and meta-analysis. *Clin Obes* 2017; 7(2):105-114.

(10) Compernolle S, De CK, Lakerveld J, Mackenbach JD, Nijpels G, Oppert JM et al. A RE-AIM evaluation of evidence-based multi-level interventions to improve obesity-related behaviours in adults: a systematic review (the SPOTLIGHT project). *Int J Behav Nutr Phys Act* 2014; 11:147.

(11) Danielsen KK, Svendsen M, Maehlum S, Sundgot-Borgen J. Changes in body composition, cardiovascular disease risk factors, and eating behavior after an intensive lifestyle intervention with high volume of physical activity in severely obese subjects: a prospective clinical controlled trial. *J Obes* 2013; 2013:325464.

(12) de Vos BC, Runhaar J, van MM, Krul M, Bierma-Zeinstra SM. Long-term effects of a randomized, controlled, tailor-made weight-loss intervention in primary care on the health and lifestyle of overweight and obese women. *Am J Clin Nutr* 2016; 104(1):33-40.

(13) Gray CM, Hunt K, Mutrie N, Anderson AS, Treweek S, Wyke S. Weight management for overweight and obese men delivered through professional football clubs: a pilot randomized trial. *Int J Behav Nutr Phys Act* 2013; 10:121.

(14) Greaves C, Gillison F, Stathi A, Bennett P, Reddy P, Dunbar J et al. Waste the waist: a pilot randomised controlled trial of a primary care based intervention to support lifestyle change in people with high cardiovascular risk. *Int J Behav Nutr Phys Act* 2015; 12:1.

(15) Gudzune KA, Bleich SN, Clark JM. Efficacy of Commercial Weight-Loss Programs. *Ann Intern Med* 2015; 163(5):399.

(16) Hassan Y, Head V, Jacob D, Bachmann MO, Diu S, Ford J. Lifestyle interventions for weight loss in adults with severe obesity: a systematic review. *Clin Obes* 2016; 6(6):395-403.

(17) Huseinovic E, Bertz F, Leu AM, Hellebo JE, Winkvist A, Brekke HK. Effectiveness of a weight loss intervention in postpartum women: results from a randomized controlled trial in primary health care. *Am J Clin Nutr* 2016; 104(2):362-370.

(18) Johns DJ, Hartmann-Boyce J, Jebb SA, Aveyard P. Diet or exercise interventions vs combined behavioral weight management programs: a systematic review and meta-analysis of direct comparisons. *J Acad Nutr Diet* 2014; 114(10):1557-1568.

(19) Johnston BC, Kanters S, Bandayrel K, Wu P, Naji F, Siemieniuk RA et al. Comparison of weight loss among named diet programs in overweight and obese adults: a meta-analysis. *JAMA* 2014; 312(9):923-933.

(20) Jull J, Stacey D, Beach S, Dumas A, Strychar I, Ufholz LA et al. Lifestyle interventions targeting body weight changes during the menopause transition: a systematic review. *J Obes* 2014; 2014:824310.

(21) Mason C, Xiao L, Imayama I, Duggan C, Wang CY, Korde L et al. Vitamin D3 supplementation during weight loss: a double-blind randomized controlled trial. *Am J Clin Nutr* 2014; 99(5):1015-1025.

(22) Moreno B, Bellido D, Sajoux I, Goday A, Saavedra D, Crujeiras AB et al. Comparison of a very low-calorie-ketogenic diet with a standard low-calorie diet in the treatment of obesity. *Endocrine* 2014; 47(3):793-805.

(23) Neville CE, McKinley MC, Holmes VA, Spence D, Woodside JV. The effectiveness of weight management interventions in breastfeeding women--a systematic review and critical evaluation. *Birth* 2014; 41(3):223-236.

(24) Partridge SR, Juan SJ, McGeechan K, Bauman A, Allman-Farinelli M. Poor quality of external validity reporting limits generalizability of overweight and/or obesity lifestyle prevention interventions in young adults: a systematic review. *Obes Rev* 2015; 16(1):13-31.

(25) Pekkarinen T, Kaukua J, Mustajoki P. Long-term weight maintenance after a 17-week weight loss intervention with or without a one-year maintenance program: a randomized controlled trial. *J Obes* 2015; 2015:651460.

(26) Phelan S, Hagobian T, Brannen A, Hatley KE, Schaffner A, Munoz-Christian K et al. Effect of an Internet-Based Program on Weight Loss for Low-Income Postpartum Women: A Randomized Clinical Trial. *JAMA* 2017; 317(23):2381-2391.

(27) Poulsen SK, Crone C, Astrup A, Larsen TM. Long-term adherence to the New Nordic Diet and the effects on body weight, anthropometry and blood pressure: a 12-month follow-up study. *Eur J Nutr* 2015; 54(1):67-76.

(28) Runhaar J, van MM, Reijman M, Willemsen S, Oei EH, Vroegindeweij D et al. Prevention of knee osteoarthritis in overweight females: the first preventive randomized controlled trial in osteoarthritis. *Am J Med* 2015; 128(8):888-895.

(29) Schwingshackl L, Dias S, Hoffmann G. Impact of long-term lifestyle programmes on weight loss and cardiovascular risk factors in overweight/obese participants: a systematic review and network meta-analysis. *Syst Rev* 2014; 3:130.

(30) Unick JL, Leahey T, Kent K, Wing RR. Examination of whether early weight loss predicts 1-year weight loss among those enrolled in an Internet-based weight loss program. *Int J Obes (Lond)* 2015; 39(10):1558-1560.

(31) Wadden TA, Butryn ML, Hong PS, Tsai AG. Behavioral treatment of obesity in patients encountered in primary care settings: a systematic review. *JAMA* 2014; 312(17):1779-1791.

(32) Weerasekara YK, Roberts SB, Kahn MA, LaVertu AE, Hoffman B, Das SK. Effectiveness of Workplace Weight Management Interventions: a Systematic Review. *Curr Obes Rep* 2016; 5(2):298-306.

(33) Williams RL, Wood LG, Collins CE, Callister R. Effectiveness of weight loss interventions--is there a difference between men and women: a systematic review. *Obes Rev* 2015; 16(2):171-186.

Supporting Information 2. Sampling framework.

To ensure our volunteers would be a representative UK group, of the 20 weight management staff selected, at least 50% would be from England. Similarly, at least 50% of the 20 academic/policy maker/commissioner group would be from England. 8 of the 20 (40%) would be academics, 6 of the 20 (30%) would be policy makers and 6 of the 20 (30%) would be commissioners. At least 50% of the 10 primary care staff selected would also be from England. With regard to members of the public, more than 50% would have experience of commercial BWMPs, more than 50% would be of working age, more than 30% would be male and less than 30% would be from any one region of the UK.

Supporting Information 3. Stage 1 (outcome selection), round 1 Delphi questionnaire as it appeared to participants.

1. * I have read the Invite and Information Letter (v2.0 07/09/17 or v3.0 07/09/17) and I consent to participate in this Delphi process to develop a core outcome set for lifestyle weight management. I know that my free text comments will be analysed and may be quoted in publications arising from this work. (You have to tick YES to participate in the rest of the questionnaire.)

- Yes
- No

2. * I consent to being named as a member of this development group in the acknowledgements of any publication arising from this work. (OPTIONAL)

- Yes
- No

3.

Outcomes Using Information from First Visit

There now follows a list of potential outcomes presented under 7 categories (Demographics, Physical Measurements, Physical Activity, Diet, Comorbidities, Lifestyle Behaviours and Psychological Factors). Please rate how important you think it is for weight management services to measure and report a given outcome. By reporting the following outcomes, a weight management service will be showing what type of people are referred to and start the programme. When these outcomes are reported, it is likely that averages or percentages will be given.

Demographics (At First Visit)

Reminder: For each item please rate how important you think it is for weight management services to measure and report a given outcome. For each outcome listed below, please use the 9-point scale to rate importance.

3.1. * Age
How old participants are/the age (in years) of participants.

Not at all important — Very important

1	2	3	4	5	6	7	8	9

3.2. * Gender
How participants identify themselves with regard to being male, female or non-binary/third gender.

Not at all important — Very important

1	2	3	4	5	6	7	8	9

3.3. * Sexual Orientation
Participants' sexual identity in relation to the gender to which they are attracted e.g. heterosexual, homosexual or bisexual.

Not at all important — Very important

1	2	3	4	5	6	7	8	9

3.4. * Marital Status
Participants' situation with regard to being single, married, cohabiting, separated, divorced or widowed.

Not at all important — Very important

1	2	3	4	5	6	7	8	9

3.5. * Parity (women only)
The number of children a participant has given birth to.

Not at all important — Very important

1	2	3	4	5	6	7	8	9

3.6. * Ethnicity
The social group with common national and cultural tradition that participants identify as belonging to e.g. White/White British, Asian/Asian British, Black/African/Caribbean/Black British.

Not at all important — Very important

1	2	3	4	5	6	7	8	9

3.7. * Religion
The faith participants follow e.g. Christianity, Judaism, Islam, Hinduism, Sikhism etc.

Not at all important — Very important

1	2	3	4	5	6	7	8	9

3.8. * Physical Disability
Whether participants have a recognised physical disadvantage or handicap.

Not at all important — Very important

1	2	3	4	5	6	7	8	9

3.9. * Learning Disability
Whether participants have a recognised mental/cognitive disadvantage or handicap.

Not at all important — Very important

1	2	3	4	5	6	7	8	9

3.10. * Deprivation Category
A measure of the level of poverty in the area in which the participant lives.

Not at all important — Very important

1	2	3	4	5	6	7	8	9

3.11. * Housing Tenure
The financial arrangement under which a participant is entitled to live in their home. The most common forms of housing tenure are tenancy, in which rent is paid to a landlord, and owner-occupancy in which the property is owned outright or owned with a mortgage or loan.

Not at all important — Very important

1	2	3	4	5	6	7	8	9

3.12. * Education
The highest level of schooling participants have reached.

Not at all important — Very important

1	2	3	4	5	6	7	8	9

3.13. * Employment
Whether participants are in paid work and what type of job they have.

Not at all important — Very important

1	2	3	4	5	6	7	8	9

3.14. * Working Pattern
How participants' contractual hours are split across their working week e.g. shift work, full time work, part time work.

Not at all important — Very important

1	2	3	4	5	6	7	8	9

3.15. * Family History of Obesity
Whether participants have close family members who have been identified as being overweight/obese.

Not at all important — Very important

1	2	3	4	5	6	7	8	9

3.16. * Weight Loss History
Whether participants have made previous attempts to lose weight and, if so, whether these attempts were successful. If attempts were successful, how much weight was lost and how was the weight loss achieved.

Not at all important — Very important

1	2	3	4	5	6	7	8	9

3.17. You may use this space to elaborate on your views in relation to any of the above outcomes. Please note to which of the above outcomes your comment(s) relates

Maximum length: 5000 characters. Characters left: 5000

3.18. Please add any additional outcomes in the box below.

Maximum length: 5000 characters. Characters left: 5000

Physical Measurements (At First Visit)

Reminder: For each item please rate how important you think it is for weight management services to measure and report a given outcome. For each outcome listed below, please use the 9-point scale to rate importance.

3.19. * Weight
The measurement of how heavy a participant is in kilograms (kg) or stones and pounds.

Not at all important — Very important

1	2	3	4	5	6	7	8	9

3.20. * Body Mass Index (BMI)
An approximate measure of whether a participant is overweight or underweight, calculated by dividing their weight in kilograms by the square of their height in metres.

Not at all important — Very important

1	2	3	4	5	6	7	8	9

3.21. * Waist Circumference
A measurement taken around the abdomen at the level of the umbilicus (belly button). Health experts use waist circumference to screen patients for possible weight-related health problems.

Not at all important — Very important

1	2	3	4	5	6	7	8	9

3.22. * Waist to Hip Ratio
An indicator of health and the risk of developing serious health conditions, obtained by calculating the ratio of a participant's waist circumference to their hip circumference.

Not at all important — Very important

1	2	3	4	5	6	7	8	9

3.23. * Blood Pressure
The pressure of blood in the arteries, the vessels that carry blood from the heart to the rest of the body. A certain amount of pressure is required to get the blood around the body but consistently high blood pressure increases the risk of cardiovascular disease.

Not at all important — Very important

1	2	3	4	5	6	7	8	9

3.24. * Heart Rate
The number of time the heart beats per minute. Being overweight is associated with increased heart rate.

Not at all important — Very important

1	2	3	4	5	6	7	8	9

3.25. * Fat Mass/Body Composition
The portion of the human body that is composed strictly of fat or muscle.

Not at all important — Very important

1	2	3	4	5	6	7	8	9

3.26. You may use this space to elaborate on your views in relation to any of the above outcomes. Please note to which of the above outcomes your comment(s) relates.

Maximum length: 5000 characters. Characters left: 5000

3.27. Please add any additional outcomes in the box below.

Maximum length: 5000 characters. Characters left: 5000

Physical Activity (At First Visit)

Reminder: For each item please rate how important you think it is for weight management services to measure and report a given outcome. For each outcome listed below, please use the 9-point scale to rate importance.

3.28. * Non Leisure Time Physical Activity
Physical activity performed during a participant's regular occupation, housework or transportation.

Not at all important — Very important

1	2	3	4	5	6	7	8	9

3.29. * Leisure Time Physical Activity
Physical activity performed during exercise, recreation or any time other than during a participant's regular occupation, housework, or transportation.

Not at all important — Very important

1	2	3	4	5	6	7	8	9

3.30. * Fitness
The ability of participants to undertake sustained physical exertion without undue breathlessness.

Not at all important — Very important

1	2	3	4	5	6	7	8	9

3.31. * Sedentary Time
Time, during waking hours, when there is little or no physical activity. Examples of sedentary behaviours include reading, watching television, playing video games and sitting at a computer. A sedentary lifestyle may contribute to weight gain.

Not at all important — Very important

1	2	3	4	5	6	7	8	9

3.32. You may use this space to elaborate on your views in relation to any of the above outcomes. Please note to which of the above outcomes your comment(s) relates.

Maximum length: 5000 characters. Characters left: 5000

3.33. Please add any additional outcomes in the box below.

Maximum length: 5000 characters. Characters left: 5000

Diet (At First Visit)

Reminder: For each item please rate how important you think it is for weight management services to measure and report a given outcome. For each outcome listed below, please use the 9-point scale to rate importance.

3.34. * Vegetarian
Whether participants follow a vegetarian diet wherein they do not eat meat and fish.

Not at all important — Very important

1	2	3	4	5	6	7	8	9

3.35. * Vegan
Whether participants follow a vegan diet wherein they do not consume any animal products.

Not at all important — Very important

1	2	3	4	5	6	7	8	9

3.36. * Daily Calorie Consumption
The amount of fuel (food), measured in kilocalories (kcal), that a participant consumes (by eating or drinking) in a twenty four hour period in order to provide their body with energy to perform bodily processes e.g. maintenance of body temperature, movement of muscles etc. To maintain a stable weight, the energy going into the body must be the same as the energy being used up through normal body functions and physical activity. An important part of a healthy diet is eating the right amount of calories; balancing the energy going into the body with the energy being used.

Not at all important — Very important

1	2	3	4	5	6	7	8	9

3.37. * Daily Protein Intake
The amount of protein (a nutrient found in meat, fish, milk, eggs and pulses) that a participant consumes in a twenty four hour period.

Not at all important — Very important

1	2	3	4	5	6	7	8	9

3.38. * Daily Saturated Fat Intake
The amount of saturated fat (a type of fat found in meat, eggs, milk, cheese, etc.) that a participant consumes in a twenty four hour period. Eating too much saturated fat can increase the levels of cholesterol in the blood which, in turn, can increase the risk of cardiovascular disease.

Not at all important — Very important

1	2	3	4	5	6	7	8	9

3.39. * Daily Salt Intake
The amount of salt that a participant consumes in a twenty four hour period. Too much salt can raise blood pressure, increasing the risk of health problems such as cardiovascular disease.

Not at all important — Very important

1	2	3	4	5	6	7	8	9

3.40. * Daily Carbohydrate Intake
The amount of carbohydrate (a nutrient found in starchy foods, including potatoes, rice, pasta and bread) that a participant consumes in a twenty four hour period.

Not at all important — Very important

1	2	3	4	5	6	7	8	9

3.41. * Daily Free Sugar Intake
The amount of free sugar a participant consumes in a twenty four hour period. Free sugar is defined by the World Health Organization as all monosaccharides and disaccharides added to foods by the manufacturer, cook, or consumer, plus sugars naturally present in honey, syrups, and fruit juices. It is used to distinguish between the sugars that are naturally present in fully unrefined carbohydrates such as brown rice, whole wheat pasta, fruit, etc. and those sugars (or carbohydrates) that have been, to some extent, refined (normally by humans but sometimes by animals, such as the free sugars present in honey). Too much free sugar can cause weight gain and increase the risk of type 2 diabetes.

Not at all important — Very important

1	2	3	4	5	6	7	8	9

3.42. * Daily Fruit & Vegetable Intake
The number of portions of fruit and vegetables a participant consumes in a twenty four hour period. Guidelines recommend that 5 portions of fruit and vegetables are consumed every day as part of a healthy diet.

Not at all important — Very important

1	2	3	4	5	6	7	8	9

3.43. * Daily Fibre Intake
The amount of fibre that a participant consumes in a twenty four hour period. Fibre is a dietary material found in food that comes from plants. Fibre is an important part of a healthy balanced diet and can help prevent heart disease, diabetes, weight gain and some cancers. It can also improve digestive health.

Not at all important — Very important

1	2	3	4	5	6	7	8	9

3.44. * Daily Alcohol Consumption
The number of units of alcohol a participant consumes in a twenty four hour period. Units are a simple way of expressing the quantity of pure alcohol in a drink.

Not at all important								Very important
1	2	3	4	5	6	7	8	9

3.45. You may use this space to elaborate on your views in relation to any of the above outcomes. Please note to which of the above outcomes your comment(s) relates.

Maximum length: 5000 characters. Characters left: 5000

3.46. Please add any additional outcomes in the box below.

Maximum length: 5000 characters. Characters left: 5000

Comorbidities (Other Diseases/ Conditions A Participant May Have*)

The final selected outcome will be a simple percentage of participants with the condition or may be specific blood test results e.g. blood cholesterol).

*N.B. Psychological/mental health conditions are not included here but in a subsequent section.

Reminder: For each item please rate how important you think it is for weight management services to measure and report a given outcome. For each outcome listed below, please use the 9-point scale to rate importance.

3.47. * Cardiovascular Risk
A measure of how likely participants are to develop cardiovascular disease, including heart disease and stroke.

Not at all important								Very important
1	2	3	4	5	6	7	8	9

3.48. * Diabetes Status
Whether a participant has diabetes, a condition which occurs when the body doesn't produce enough insulin to function properly, or the body's cells don't react to insulin. This means glucose stays in the blood and isn't used as fuel for energy. Type 2 diabetes is often associated with obesity and an increased risk of developing cardiovascular disease.

Not at all important								Very important
1	2	3	4	5	6	7	8	9

3.49. * High Future Risk of Diabetes (Impaired Fasting Glucose, Impaired Glucose Tolerance, Raised HbA1c Levels)
Whether measures of the amount of glucose in a participant’s blood suggests he/she is likely to develop type 2 diabetes in the future.

Not at all important								Very important
1	2	3	4	5	6	7	8	9

3.50. * High Blood Pressure
Whether a participant has high blood pressure. High blood pressure increases the risk of developing cardiovascular disease.

Not at all important								Very important
1	2	3	4	5	6	7	8	9

3.51. * High Cholesterol/Lipids
A measure of whether a participant has an abnormal amount of fat and/or cholesterol, known as lipids, in their blood (also called dyslipidaemia). Being overweight can increase the likelihood of developing dyslipidaemia. Dyslipidaemia is associated with increased risk of developing cardiovascular disease.

Not at all important								Very important
1	2	3	4	5	6	7	8	9

3.52. * Obstructive Sleep Apnoea
Whether participants have obstructive sleep apnoea, a condition wherein the walls of the throat relax and narrow during sleep, interrupting normal breathing. Being overweight can increase the risk of developing obstructive sleep apnoea.

Not at all important								Very important
1	2	3	4	5	6	7	8	9

3.53. * Osteoarthritis
Whether participants have osteoarthritis, a condition that causes joints to become painful and stiff. Being overweight puts excess strain on the joints and can therefore increase the risk of developing osteoarthritis.

Not at all important								Very important
1	2	3	4	5	6	7	8	9

3.54. * Chronic Back Pain
Whether participants have back pain which has lasted more than 12 weeks. Being overweight can increase the risk of developing back pain.

Not at all important								Very important
1	2	3	4	5	6	7	8	9

3.55. * Psoriasis
Whether participants have psoriasis, a skin disease marked by red, itchy, scaly patches. Being overweight may increase the risk of developing psoriasis.

Not at all important								Very important
1	2	3	4	5	6	7	8	9

3.56. * Chronic Kidney Disease
Whether participants have experienced progressive loss in kidney function over a period of months or years. High blood pressure, high cholesterol and diabetes may cause chronic kidney disease.

Not at all important								Very important
1	2	3	4	5	6	7	8	9

3.57. * Non Alcoholic Fatty Liver Disease (NAFLD)
Whether participants have a condition caused by a build-up of fat in the liver. NAFLD is usually seen in people who are overweight or obese.

Not at all important								Very important
1	2	3	4	5	6	7	8	9

3.58. * Mobility Issues
Whether participants are unable to move with ease and without restriction. Being overweight has been associated with restricted mobility.

Not at all important								Very important
1	2	3	4	5	6	7	8	9

3.59. * Advised To Lose Weight Prior To Routine Surgery
Whether a participant's GP has recommended he/she lose weight before having a standard surgical procedure to reduce the risk of suffering complications while under general anaesthetic.

Not at all important								Very important
1	2	3	4	5	6	7	8	9

3.60. * Polycystic Ovary Syndrome (women only)
Whether participants have a condition characterised by the accumulation of numerous cysts (fluid-filled sacs) on the ovaries and associated with high male hormone levels, absent ovulation and other metabolic disturbances. Being overweight can worsen symptoms of polycystic ovary syndrome.

Not at all important								Very important
1	2	3	4	5	6	7	8	9

3.61. * Infertility
Whether participants are unable to conceive despite having regular unprotected sex. Obesity has been linked to infertility.

Not at all important								Very important
1	2	3	4	5	6	7	8	9

3.62. * Asthma
Whether participants have asthma, a lung condition which causes occasional breathing difficulties. Being overweight is known to worsen asthma symptoms.

Not at all important								Very important
1	2	3	4	5	6	7	8	9

3.63. * Coeliac Disease
Whether participants have coeliac disease, a digestive condition caused by an adverse reaction to the dietary protein, gluten. Following a gluten-free diet helps control symptoms and prevents long-term consequences of coeliac disease.

Not at all important								Very important
1	2	3	4	5	6	7	8	9

3.64. * Other Health Conditions Requiring A Specialist Diet
Whether participants have a health condition (other than coeliac disease) which means they must follow a particular diet e.g. kidney disease, metabolic disorders etc.

Not at all important								Very important
1	2	3	4	5	6	7	8	9

3.65. * Advised To Follow The Low FODMAP Diet
Whether it has been recommended to participants that they follow the low FODMAP diet, a specialist diet low in certain carbohydrates, in order to treat gut symptoms such as bloating, wind, abdominal pain, an altered bowel habit etc.

Not at all important								Very important
1	2	3	4	5	6	7	8	9

3.66. You may use this space to elaborate on your views in relation to any of the above outcomes. Please note to which of the above outcomes your comment(s) relates.

Maximum length: 5000 characters. Characters left: 5000

3.67. Please add any additional outcomes in the box below.

Maximum length: 5000 characters. Characters left: 5000

Lifestyle Behaviours (At Baseline)

Reminder: For each item please rate how important you think it is for weight management services to measure and report a given outcome. For each outcome listed below, please use the 9-point scale to rate importance.

3.68. * Smoking Status
Whether participants smoke tobacco regularly. Smoking can increase the risk of developing cardiovascular disease.

Not at all important | Very important

1	2	3	4	5	6	7	8	9

3.69. * Other Addictive Behaviour
Whether participants have a dependency on a particular substance (other than food) or behaviour e.g. alcohol, narcotics, sex etc.

Not at all important | Very important

1	2	3	4	5	6	7	8	9

3.70. You may use this space to elaborate on your views in relation to any of the above outcomes. Please note to which of the above outcomes your comment(s) relates.

Maximum length: 5000 characters. Characters left: 5000

3.71. Please add any additional outcomes in the box below.

Maximum length: 5000 characters. Characters left: 5000

Psychological Factors (At Baseline)

It is well known that people who are overweight or obese can have a range of psychological complications which may be either a cause or consequence of their weight.

Reminder: For each item please rate how important you think it is for weight management services to measure and report a given outcome. For each outcome listed below, please use the 9-point scale to rate importance.

3.72. * Self Esteem
How participants feel about their own worth.

Not at all important | Very important

1	2	3	4	5	6	7	8	9

3.73. * Self Confidence
How participants feel about their own abilities.

Not at all important | Very important

1	2	3	4	5	6	7	8	9

3.74. * Importance of Weight Loss
How important participants feel it is for them to lose weight.

Not at all important | Very important

1	2	3	4	5	6	7	8	9

3.75. * Confidence in Ability to Lose Weight
How sure participants feel that they will be able to lose weight.

Not at all important | Very important

1	2	3	4	5	6	7	8	9

3.76. * Body Image
A participant's perception of how their own body looks and how attractive it is.

Not at all important | Very important

1	2	3	4	5	6	7	8	9

3.77. * Depression
Whether a participant suffers from a mental illness characterised by a profound and persistent feeling of sadness or despair and/or a loss of interest in things that once were pleasurable.

Not at all important | Very important

1	2	3	4	5	6	7	8	9

3.78. * Anxiety
Whether a participant suffers from a disorder characterised by nervousness, fear, apprehension and worrying.

Not at all important | Very important

1	2	3	4	5	6	7	8	9

3.79. * Suicidal Thoughts
Whether a participant has thoughts about killing himself/herself.

Not at all important Very important

1	2	3	4	5	6	7	8	9

3.80. * Quality of Life (QoL) Score
A measure of the general well-being of participants.

Not at all important Very important

1	2	3	4	5	6	7	8	9

3.81. * Overall Quality of Sleep
How well a participant sleeps based on how tired they feel when waking and throughout the day, how well rested and relaxed they feel on waking, the number of times they awake during the night etc.

Not at all important Very important

1	2	3	4	5	6	7	8	9

3.82. * Relationship With Family
How participants rate the relationships they have with family members e.g. a good relationship with communication and time spent together or a poor relationship with little communication and little time spent together.

Not at all important Very important

1	2	3	4	5	6	7	8	9

3.83. * Relationship With Friends
Whether participants have friends and, if so, how they rate the relationships they have with them e.g. a good relationship with communication and time spent together or a poor relationship with little communication and little time spent together.

Not at all important Very important

1	2	3	4	5	6	7	8	9

3.84. * Social Support
The physical and emotional comfort given by family, friends, co-workers and others.

Not at all important Very important

1	2	3	4	5	6	7	8	9

3.85. * Binge Eating Disorder
Whether participants have an illness which causes them to overeat on a regular basis.

Not at all important Very important

1	2	3	4	5	6	7	8	9

3.86. * Autism
Whether participants have autism, a lifelong, developmental disability which affects how a person communicates with and relates to other people, and how they experience the world around them.

Not at all important Very important

1	2	3	4	5	6	7	8	9

3.87. * Personality Disorders
Whether participants have conditions wherein they differ significantly from an average person, in terms of how they think, perceive, feel or relate to others.

Not at all important Very important

1	2	3	4	5	6	7	8	9

3.88. You may use this space to elaborate on your views in relation to any of the above outcomes. Please note to which of the above outcomes your comment(s) relates.

Maximum length: 5000 characters. Characters left: 5000

3.89. Please add any additional outcomes in the box below.

Maximum length: 5000 characters. Characters left: 5000

4.

Outcomes Using Information from Follow-up Visits and The End of The Programme

There now follows a list of potential outcomes presented under 7 categories (Demographics, Physical Measurements, Physical Activity, Diet, Comorbidities, Lifestyle Behaviours and Psychological Factors). Please rate how important you think it is for weight management services to measure and report a given outcome.

By reporting the following outcomes, a weight management service will be showing what effect the programme has had on participants. When these outcomes are reported, it is likely that averages or percentages will be given.

Change in Physical Measurements (At Follow Up/ End of Programme)

Reminder: For each item please rate how important you think it is for weight management services to measure and report a given outcome. For each outcome listed below, please use the 9-point scale to rate importance.

4.1. * Weight
The measurement of how heavy a participant is in kilograms (kg) or stones and pounds.

Not at all important — Very important

1	2	3	4	5	6	7	8	9

4.2. * Body Mass Index (BMI)
An approximate measure of whether a participant is overweight or underweight, calculated by dividing their weight in kilograms by the square of their height in metres.

Not at all important — Very important

1	2	3	4	5	6	7	8	9

4.3. * Waist Circumference
A measurement taken around the abdomen at the level of the umbilicus (belly button). Health experts use waist circumference to screen patients for possible weight-related health problems.

Not at all important — Very important

1	2	3	4	5	6	7	8	9

4.4. * Waist to Hip Ratio
An indicator of health and the risk of developing serious health conditions, obtained by calculating the ratio of a participant's waist circumference to their hip circumference.

Not at all important — Very important

1	2	3	4	5	6	7	8	9

4.5. * Blood Pressure
The pressure of blood in the arteries, the vessels that carry blood from the heart to the rest of the body. A certain amount of pressure is required to get the blood around the body but consistently high blood pressure increases the risk of cardiovascular disease.

Not at all important — Very important

1	2	3	4	5	6	7	8	9

4.6. * Heart Rate
The number of time the heart beats per minute. Being overweight is associated with increased heart rate.

Not at all important — Very important

1	2	3	4	5	6	7	8	9

4.7. * Fat Mass/Body Composition
The portion of the human body that is composed strictly of fat or muscle.

Not at all important — Very important

1	2	3	4	5	6	7	8	9

4.8. You may use this space to elaborate on your views in relation to any of the above outcomes. Please note to which of the above outcomes your comment(s) relates.

Maximum length: 5000 characters. Characters left: 5000

4.9. Please add any additional outcomes in the box below.

Maximum length: 5000 characters. Characters left: 5000

Change in Physical Activity (At Follow Up/ End of Programme)

Reminder: For each item please rate how important you think it is for weight management services to measure and report a given outcome. For each outcome listed below, please use the 9-point scale to rate importance.

4.10. * Non Leisure Time Physical Activity
Physical activity performed during a participant's regular occupation, housework or transportation.

Not at all important — Very important

1	2	3	4	5	6	7	8	9

4.11. * Leisure Time Physical Activity
Physical activity performed during exercise, recreation or any time other than during a participant's regular occupation, housework, or transportation.

Not at all important — Very important

1	2	3	4	5	6	7	8	9

4.12. * Fitness
The ability of participants to undertake sustained physical exertion without undue breathlessness.

Not at all important — Very important

1	2	3	4	5	6	7	8	9

4.13. * Sedentary Time
Time, during waking hours, when there is little or no physical activity. Examples of sedentary behaviours include reading, watching television, playing video games and sitting at a computer. A sedentary lifestyle may contribute to weight gain.

Not at all important — Very important

1	2	3	4	5	6	7	8	9

4.14. You may use this space to elaborate on your views in relation to any of the above outcomes. Please note to which of the above outcomes your comment(s) relates.

Maximum length: 5000 characters. Characters left: 5000

4.15. Please add any additional outcomes in the box below.

Maximum length: 5000 characters. Characters left: 5000

Change in Diet (At Follow Up/ End of Programme)

Reminder: For each item please rate how important you think it is for weight management services to measure and report a given outcome. For each outcome listed below, please use the 9-point scale to rate importance.

4.16. * Daily Calorie Consumption
The amount of fuel (food), measured in kilocalories (kcal), that a participant consumes (by eating or drinking) in a twenty four hour period in order to provide their body with energy to perform bodily processes e.g. maintenance of body temperature, movement of muscles etc. To maintain a stable weight, the energy going into the body must be the same as the energy being used up through normal body functions and physical activity. An important part of a healthy diet is eating the right amount of calories; balancing the energy going into the body with the energy being used.

Not at all important — Very important

1	2	3	4	5	6	7	8	9

4.17. * Daily Protein Intake
The amount of protein (a nutrient found in meat, fish, milk, eggs and pulses) that a participant consumes in a twenty four hour period.

Not at all important — Very important

1	2	3	4	5	6	7	8	9

4.18. * Daily Saturated Fat Intake
The amount of saturated fat (a type of fat found in meat, eggs, milk, cheese, etc.) that a participant consumes in a twenty four hour period. Eating too much saturated fat can increase the levels of cholesterol in the blood which, in turn, can increase the risk of cardiovascular disease.

Not at all important — Very important

1	2	3	4	5	6	7	8	9

4.19. * Daily Salt Intake
The amount of salt that a participant consumes in a twenty four hour period. Too much salt can raise blood pressure, increasing the risk of health problems such as cardiovascular disease.

Not at all important — Very important

1	2	3	4	5	6	7	8	9

4.20. * Daily Carbohydrate Intake
The amount of carbohydrate (a nutrient found in starchy foods, including potatoes, rice, pasta and bread) that a participant consumes in a twenty four hour period.

Not at all important — Very important

1	2	3	4	5	6	7	8	9

4.21. * Daily Free Sugar Intake
The amount of free sugar a participant consumes in a twenty four hour period. Free sugar is defined by the World Health Organization as all monosaccharides and disaccharides added to foods by the manufacturer, cook, or consumer, plus sugars naturally present in honey, syrups, and fruit juices. It is used to distinguish between the sugars that are naturally present in fully unrefined carbohydrates such as brown rice, whole wheat pasta, fruit, etc. and those sugars (or carbohydrates) that have been, to some extent, refined (normally by humans but sometimes by animals, such as the free sugars present in honey). Too much free sugar can cause weight gain and increase the risk of type 2 diabetes.

Not at all important — Very important

1	2	3	4	5	6	7	8	9

4.22. * Daily Fruit & Vegetable Intake
The number of portions of fruit and vegetables a participant consumes in a twenty four hour period. Guidelines recommend that 5 portions of fruit and vegetables are consumed every day as part of a healthy diet.

Not at all important — Very important

1	2	3	4	5	6	7	8	9

4.23. * Daily Fibre Intake
The amount of fibre that a participant consumes in a twenty four hour period. Fibre is a dietary material found in food that comes from plants. Fibre is an important part of a healthy balanced diet and can help prevent heart disease, diabetes, weight gain and some cancers. It can also improve digestive health.

Not at all important — Very important

1	2	3	4	5	6	7	8	9

4.24. * Daily Alcohol Consumption
The number of units of alcohol a participant consumes in a twenty four hour period. Units are a simple way of expressing the quantity of pure alcohol in a drink.

Not at all important — Very important

1	2	3	4	5	6	7	8	9

4.25. You may use this space to elaborate on your views in relation to any of the above outcomes. Please note to which of the above outcomes your comment(s) relates.

Maximum length: 5000 characters. Characters left: 5000

4.26. Please add any additional outcomes in the box below.

Maximum length: 5000 characters. Characters left: 5000

Changes in Comorbidities (Other Diseases/ Conditions A Participant May Have*)

The final selected outcome will be a simple percentage of participants with the condition and the change, or may be a change in a specific blood test result (e.g. blood cholesterol).

*N.B. Psychological/mental health conditions are not included here but in a subsequent section.

Reminder: For each item please rate how important you think it is for weight management services to measure and report a given outcome. For each outcome listed below, please use the 9-point scale to rate importance.

4.27. * Cardiovascular Risk
A measure of how likely participants are to develop cardiovascular disease, including heart disease and stroke.

Not at all important — Very important

1	2	3	4	5	6	7	8	9

4.28. * Diabetes Status
Whether a participant has diabetes, a condition which occurs when the body doesn't produce enough insulin to function properly, or the body's cells don't react to insulin. This means glucose stays in the blood and isn't used as fuel for energy. Type 2 diabetes is often associated with obesity and an increased risk of developing cardiovascular disease.

Not at all important — Very important

1	2	3	4	5	6	7	8	9

4.29. * High Future Risk of Diabetes (Impaired Fasting Glucose, Impaired Glucose Tolerance, Raised HbA1c Levels)
Whether measures of the amount of glucose in a participant's blood suggests he/she is likely to develop type 2 diabetes in the future.

Not at all important — Very important

1	2	3	4	5	6	7	8	9

4.30. * High Blood Pressure
Whether a participant has high blood pressure. High blood pressure increases the risk of developing cardiovascular disease.

Not at all important — Very important

1	2	3	4	5	6	7	8	9

4.31. * High Cholesterol/Lipids
A measure of whether a participant has an abnormal amount of fat and/or cholesterol, known as lipids, in their blood (also called dyslipidaemia). Being overweight can increase the likelihood of developing dyslipidaemia. Dyslipidaemia is associated with increased risk of developing cardiovascular disease.

Not at all important — Very important

1	2	3	4	5	6	7	8	9

4.32. * Obstructive Sleep Apnoea
Whether participants have obstructive sleep apnoea, a condition wherein the walls of the throat relax and narrow during sleep, interrupting normal breathing. Being overweight can increase the risk of developing obstructive sleep apnoea.

Not at all important — Very important

1	2	3	4	5	6	7	8	9

4.33. * Osteoarthritis
Whether participants have osteoarthritis, a condition that causes joints to become painful and stiff. Being overweight puts excess strain on the joints and can therefore increase the risk of developing osteoarthritis.

Not at all important								Very important
1	2	3	4	5	6	7	8	9

4.34. * Chronic Back Pain
Whether participants have back pain which has lasted more than 12 weeks. Being overweight can increase the risk of developing back pain.

Not at all important								Very important
1	2	3	4	5	6	7	8	9

4.35. * Psoriasis
Whether participants have psoriasis, a skin disease marked by red, itchy, scaly patches. Being overweight may increase the risk of developing psoriasis.

Not at all important								Very important
1	2	3	4	5	6	7	8	9

4.36. * Chronic Kidney Disease
Whether participants have experienced progressive loss in kidney function over a period of months or years. High blood pressure, high cholesterol and diabetes may cause chronic kidney disease.

Not at all important								Very important
1	2	3	4	5	6	7	8	9

4.37. * Non Alcoholic Fatty Liver Disease (NAFLD)
Whether participants have a condition caused by a build-up of fat in the liver. NAFLD is usually seen in people who are overweight or obese.

Not at all important								Very important
1	2	3	4	5	6	7	8	9

4.38. * Mobility Issues
Whether participants are unable to move with ease and without restriction. Being overweight has been associated with restricted mobility.

Not at all important								Very important
1	2	3	4	5	6	7	8	9

4.39. * Advised To Lose Weight Prior To Routine Surgery
Whether a participant's GP has recommended he/she lose weight before having a standard surgical procedure to reduce the risk of suffering complications while under general anaesthetic.

Not at all important								Very important
1	2	3	4	5	6	7	8	9

4.40. * Polycystic Ovary Syndrome (women only)
Whether participants have a condition characterised by the accumulation of numerous cysts (fluid-filled sacs) on the ovaries and associated with high male hormone levels, absent ovulation and other metabolic disturbances. Being overweight can worsen symptoms of polycystic ovary syndrome.

Not at all important								Very important
1	2	3	4	5	6	7	8	9

4.41. * Infertility
Whether participants are unable to conceive despite having regular unprotected sex. Obesity has been linked to infertility.

Not at all important								Very important
1	2	3	4	5	6	7	8	9

4.42. * Asthma
Whether participants have asthma, a lung condition which causes occasional breathing difficulties. Being overweight is known to worsen asthma symptoms.

Not at all important								Very important
1	2	3	4	5	6	7	8	9

4.43. You may use this space to elaborate on your views in relation to any of the above outcomes. Please note to which of the above outcomes your comment(s) relates.

Maximum length: 5000 characters. Characters left: 5000

4.44. Please add any additional outcomes in the box below.

Maximum length: 5000 characters. Characters left: 5000

Change in Lifestyle Behaviours (At Follow Up/End of Programme)

Reminder: For each item please rate how important you think it is for weight management services to measure and report a given outcome. For each outcome listed below, please use the 9-point scale to rate importance.

4.45. * Smoking Status
Whether participants smoke tobacco regularly. Smoking can increase the risk of developing cardiovascular disease.

Not at all important								Very important
1	2	3	4	5	6	7	8	9

4.46. * Other Addictive Behaviour
Whether participants have a dependency on a particular substance (other than food) or behaviour e.g. alcohol, narcotics, sex etc.

Not at all important								Very important
1	2	3	4	5	6	7	8	9

4.47. You may use this space to elaborate on your views in relation to any of the above outcomes. Please note to which of the above outcomes your comment(s) relates.

Maximum length: 5000 characters. Characters left: 5000

4.48. Please add any additional outcomes in the box below.

Maximum length: 5000 characters. Characters left: 5000

Changes in Psychological Factors (At Follow Up/End of Programme)

It is well known that people who are overweight or obese can have a range of psychological complications which may be either a cause or consequence of their weight.

Reminder: For each item please rate how important you think it is for weight management services to measure and report a given outcome. For each outcome listed below, please use the 9-point scale to rate importance.

4.49. * Self Esteem
How participants feel about their own worth.

Not at all important								Very important
1	2	3	4	5	6	7	8	9

4.50. * Self Confidence
How participants feel about their own abilities.

Not at all important								Very important
1	2	3	4	5	6	7	8	9

4.51. * Importance of Weight Loss
How important participants feel it is for them to lose weight.

Not at all important								Very important
1	2	3	4	5	6	7	8	9

4.52. * Confidence in Ability to Lose Weight
How sure participants feel that they will be able to lose weight.

Not at all important								Very important
1	2	3	4	5	6	7	8	9

4.53. * Body Image
A participant's perception of how their own body looks and how attractive it is.

Not at all important								Very important
1	2	3	4	5	6	7	8	9

4.54. * Depression
Whether a participant suffers from a mental illness characterised by a profound and persistent feeling of sadness or despair and/or a loss of interest in things that once were pleasurable.

Not at all important								Very important
1	2	3	4	5	6	7	8	9

4.55. * Anxiety
Whether a participant suffers from a disorder characterised by nervousness, fear, apprehension and worrying.

Not at all important								Very important
1	2	3	4	5	6	7	8	9

4.56. * Suicidal Thoughts
Whether a participant has thoughts about killing himself/herself.

Not at all important								Very important
1	2	3	4	5	6	7	8	9

4.57. * Quality of Life (QoL) Score
A measure of the general well-being of participants.

Not at all important								Very important
1	2	3	4	5	6	7	8	9

4.58. * Overall Quality of Sleep
How well a participant sleeps based on how tired they feel when waking and throughout the day, how well rested and relaxed they feel on waking, the number of times they awake during the night etc.

Not at all important								Very important
1	2	3	4	5	6	7	8	9

4.59. * Relationship With Family
How participants rate the relationships they have with family members e.g. a good relationship with communication and time spent together or a poor relationship with little communication and little time spent together.

Not at all important								Very important
1	2	3	4	5	6	7	8	9

4.60. * Relationship With Friends
Whether participants have friends and, if so, how they rate the relationships they have with them e.g. a good relationship with communication and time spent together or a poor relationship with little communication and little time spent together.

Not at all important								Very important
1	2	3	4	5	6	7	8	9

4.61. * Social Support
The physical and emotional comfort given by family, friends, co-workers and others.

Not at all important								Very important
1	2	3	4	5	6	7	8	9

4.62. * Binge Eating Disorder
Whether participants have an illness which causes them to overeat on a regular basis.

Not at all important								Very important
1	2	3	4	5	6	7	8	9

4.63. * Autism
Whether participants have autism, a lifelong, developmental disability which affects how a person communicates with and relates to other people, and how they experience the world around them.

Not at all important								Very important
1	2	3	4	5	6	7	8	9

4.64. * Personality Disorders
Whether participants have conditions wherein they differ significantly from an average person, in terms of how they think, perceive, feel or relate to others.

Not at all important								Very important
1	2	3	4	5	6	7	8	9

4.65. You may use this space to elaborate on your views in relation to any of the above outcomes. Please note to which of the above outcomes your comment(s) relates.

Maximum length: 5000 characters. Characters left: 5000

4.66. Please add any additional outcomes in the box below.

Maximum length: 5000 characters. Characters left: 5000

Programme Specific Outcomes

These are outcomes related to how well used the service is and may be related to the service design, access and communication with GPs and hospitals.

Reminder: For each item please rate how important you think it is for weight management services to measure and report a given outcome. For each outcome listed below, please use the 9-point scale to rate importance.

4.67. * Reach
The percentage of the eligible population (people who are overweight or obese within that particular geographical area) referred to the weight management service.

Not at all important								Very important
1	2	3	4	5	6	7	8	9

4.68. * Representativeness
How representative of the entire eligible population (people who are overweight or obese within that particular geographical area) the people attending the weight

management service are.

Not at all important								Very important
1	2	3	4	5	6	7	8	9

4.69. * Attendance
How many people attended the weight management service.

Not at all important								Very important
1	2	3	4	5	6	7	8	9

4.70. * Completion
How many people finished the entire weight management programme.

Not at all important								Very important
1	2	3	4	5	6	7	8	9

4.71. * Cost Effectiveness
The value for money of the weight management service in terms of long term economic benefits to the NHS.

Not at all important								Very important
1	2	3	4	5	6	7	8	9

4.72. * Sources of Referral
Where participants received their referral to the weight management service.

Not at all important								Very important
1	2	3	4	5	6	7	8	9

4.73. * Repeat Referrals
The number of participants who were referred to the weight management service on more than one occasion.

Not at all important								Very important
1	2	3	4	5	6	7	8	9

4.74. * Referral to Specialist Services
The number of participants referred to a specialist management service after failing to lose the required amount of weight via a lifestyle weight management programme or due to a condition needing specialist input.

Not at all important								Very important
1	2	3	4	5	6	7	8	9

4.75. * Referral to Linked Services
The number of participants referred to services linked to weight management services e.g. smoking cessation services, psychiatric services etc.

Not at all important								Very important
1	2	3	4	5	6	7	8	9

4.76. * Reason for Dropout
Why those participants who did not complete the programme failed to do so.

Not at all important								Very important
1	2	3	4	5	6	7	8	9

4.77. * Participant Satisfaction
How happy/satisfied participants were with the weight loss service.

Not at all important								Very important
1	2	3	4	5	6	7	8	9

4.78. * Adverse Events/Unintended Consequences
Whether participants suffered any unfortunate side effects as a result of attending the weight loss service.

Not at all important								Very important
1	2	3	4	5	6	7	8	9

4.79. * Prescription of Anti-obesity Medication
The number of participants taking drugs to help reduce or control their weight.

Not at all important								Very important
1	2	3	4	5	6	7	8	9

4.80. You may use this space to elaborate on your views in relation to any of the above outcomes. Please note to which of the above outcomes your comment(s) relates.

Maximum length: 5000 characters. Characters left: 5000

4.81. Please add any additional outcomes in the box below.

Maximum length: 5000 characters. Characters left: 5000

5.

Length of Follow Up

Please rate how important you think it is to report outcomes at the following time-points post completion of the weight loss programme.

Reminder: For each item please rate how important you think it is for weight management services to measure and report a given outcome. For each outcome listed below, please use the 9-point scale to rate importance.

5.1. * 1 month

Not at all important — Very important

1	2	3	4	5	6	7	8	9

5.2. * 3 months

Not at all important — Very important

1	2	3	4	5	6	7	8	9

5.3. * 6 months

Not at all important — Very important

1	2	3	4	5	6	7	8	9

5.4. * 12 months

Not at all important — Very important

1	2	3	4	5	6	7	8	9

5.5. * 18 months

Not at all important — Very important

1	2	3	4	5	6	7	8	9

5.6. * 24 months

Not at all important — Very important

1	2	3	4	5	6	7	8	9

5.7. You may use this space to elaborate on your views in relation to any of the above outcomes. Please note to which of the above outcomes your comment(s) relates.

Maximum length: 5000 characters. Characters left: 5000

Thank you, please click Save to finish.

Save

Prev 1 2 3 4 ... 16 17 Next

Supporting Information 4. Stage 1 (outcome selection), round 1 Delphi qualitative analysis.

Demographics (first visit)

Generic comments

A wish not to overburden people was expressed within free-text responses; only asking about outcomes with a clear rationale for their inclusion was suggested, as in this comment from 1e5f5:

"...each question should have an underpinning reason why being asked and not simply being nosey!"

It was noted that information should be easy to collect and data that patients were happy to supply. A couple of participants said people should not feel discriminated against, including d5efb, who wrote:

"Holistic consideration of a person's individual needs is key. It does not matter what religion or sexual orientation a person has, or, to a degree, what education they have had."

A contrast was made between information for a trial, to evaluate an intervention, and information to develop a rapport with someone engaging in weight management services, by understanding their background to tailor interventions. Furthermore, 18738 stated:

"I think there is a distinction here between what is important to know in order to determine the generalisability of an RCT's findings, and what is interesting to know with regards to more observational analyses about predictors of weight loss. I have answered this in terms of the former."

Finally, c4b29 commented that socioeconomic circumstances and environment may have more of an impact on outcomes from weight management services than traits that cannot be changed (e.g. age, gender).

<u>Comments about specific outcomes</u>

3.3 Sexual Orientation: In relation to people not feeling singled out/discriminated against, this was an area quite a few respondents felt should not be an outcome.

3.5 Parity: One respondent (f6c44) stated that perhaps this should be number of pregnancies (women may have had miscarriages), but this could be sensitive information to collect. Certain participants struggled rating this one, as suggested by their comments:

"...could be reworded as it would suggest that it should only be answered by women." (ddcea)

"...you have asked me to complete question 3.5 - women only - but I am male! This question should be able to recognise the respondents gender!" (92f98)

"I have no idea under section 3.5 and have placed my score in the middle" (0764f)

3.6 Ethnicity: Several respondents mentioned a genetic predisposition to specific diseases, making this information important to gather data on. It was also stated that language barriers could prevent people from following health education material. However, f6c44 remarked on the vast diversity in ethnic groups, which could make this difficult:

"...given the complexity of our multi-cultural society, not everyone falls into a neat box."

3.7 Religion: One respondent (ff8ee) noted that this could affect the food people selected to eat.

3.8 Physical Disability: Some people who rated this as important elaborated by suggesting that individuals may be on medication, like steroids, or that the disability could affect what someone could eat and could impede activity levels.

3.9 Learning Disability: It was noted that this could make it hard for individuals to understand programmes/follow advice.

3.10 Deprivation Category: One person (4ec71) said this was closely related to housing tenure and another (df6f8) felt it might be more sensitive than education, adding that it was more clearly related to obesity.

3.11 Housing Tenure: Respondent d5cd3 did state they had rated this highly because owning a home could help reduce economic stress and, therefore, enable people to engage with weight management services.

3.12 Education: It was suggested by ff8ee that this should not be rated highly due to obesity touching all social groups, regardless of educational level.

3.14 Working Pattern: This was seen as important because it affected people's eating patterns; d5efb commented on a *"grab and go culture"* and others wrote about night/shift work being linked to obesity. It was noted individuals may not be able to work because of their weight and that

“...unemployment may increase risk of lack of motivation for change and difficulty occupying hours, and eating more." (d5cd3)

3.15 Family History of Obesity: Some respondents thought this was important because it could influence people’s approach to diet, their *“hard-wiring to eat and drink unhealthily" (d5efb).* It was also suggested that genetic influences could be important in this respect. Participant f6c44 gave this a relatively low rating because it was based on subjective information and individuals may not recognise obesity in their family. Conversely, 92f98 rated it highly because it could affect how people perceived weight and health.

3.16 Weight Loss History: It was observed that this could affect motivation, so was important. However, 25d91 commented that patients may have an extensive weight loss history, so what is asked may need to be simplified into broad approaches tried previously (e.g. medication, slimming clubs, seeing a dietician, exercise).

<u>Other areas suggested</u>

- Living arrangements, because having others to cook for could affect (negatively or positively) one’s eating choices
- 3.14 could be expanded to define level of activity or inactivity in the type of work being done
- The participant’s goals for attending weight management

Physical measurements (first visit)

There were several comments about the need to measure physical aspects to assess whether an intervention/service was effective. However, respondent 8b0d4 felt there might be a clash between such measures and the goals of weight management services (which aim to address personal goals and lifestyle.) This participant added how such a focus *"could make the service too `medicalised' and less likely to be engaged with."* Similarly, c4b29 wrote that on a first visit attention should be on finding out what people thought had led to their obesity, not on physical measures. It was clear that others felt not all the measures listed should be given a priority, for the reasons listed below.

Accuracy

BMI was dismissed by several participants as potentially inaccurate, as was waist to hip measurement because with individuals who are obese this could be difficult to get right (depending on clothes being worn, if the person is uncomfortable with undergoing or performing this measurement). It was noted that the person measuring heart rate and blood pressure had to be properly trained to interpret results. Conducting fat mass assessments could call on patients to fast and refrain from caffeine/alcohol, according to f6c44, who scored this outcome 3. It was likewise noted that the same machine needed to be used for assessing this outcome by c2e0e, who still rated it 7. This person also scored blood pressure a 9 because it was associated with heart disease, but they added it required a standardised methodology (e.g. relaxation for 5 minutes before measurement, not crossed legs, emptied bladder etc).

Clinically useful predictor

Although scoring body fat mass a 9, d5efb stated that even thin people could have *"dangerous visceral fat on the inside".* Weight was seen as important by some individuals to measure because it was related to health indicators, including things like blood pressure. However, there was a suggestion that waist-hip ratio may be better than BMI because it is more linked to possible risks from disease. It was noted that for children, blood pressure and heart rate should be avoided, unless clinically indicated, as *"it is not helpful to over medicalise their care and support" (ab65c).* Respondent ff8ee proposed that blood pressure may be normal in people classed as obese (they still scored this 7), and 4ec71 marked lower those measures that clinicians were not used to taking and, therefore, may not be as interested in.

Resources (time and equipment)

Measuring body fat/composition was seen as needing expensive equipment, which was unlikely to be available in weight management services. It was observed that where weight management services take place (e.g. church halls, community centres) might not allow for the easy collection of some outcomes listed (e.g. blood pressure, heartrate). For 8b0d4, measuring blood pressure, heart rate and fat mass/body composition called for staff training and equipment, and risked taking attention from the service. This individual added, *"It is highly recommended that weight and height are the only physical measurements taken, when thinking about simple, scalable and cost effective service provision."*

Motivating

It was suggested that telling people their blood pressure was going down and so medication for this could be stopped, might motivate people to continue with losing weight.

Other areas suggested

- Neck circumference, presence of acanthosis or signs of lipodystrophy
- Cholesterol, fasting blood sugars and thyroid stimulating hormone
- HbA1c (for diabetes status/measuring change in glycaemia)
- An Edmonton obesity severity score measured overall
- Self reported reduction in clothes size

Physical activity (first visit)

Respondent c4b29 rated all outcomes in this section low, citing over-identification of physical activity and weight loss:

"Generally this doesn't happen so people disengage with activity. The reason for being active is to reduce risk of chronic disease but between 25 and 50% not to lose weight which may be a pleasant side effect in some."

It was also noted by 8b0d4 that:

"In relation to point 3.3 - it is highly recommended that a fitness test is not suggested to be a core outcome ...not only would this put people off, but again add complexity, cost and is of little value."

Some participants felt physical activity level was not important in assessing the success of weight management services, was an added burden on services and patients, and there was a questioning of patients honesty/accuracy. Others commented that the link between sedentary behaviour and health risks made this an important outcome to measure.

Other Areas Suggested

- Frequency of bouts of activity in the day

Diet (first visit)

There was a general sense of there being too many items in this section. It was feared that collecting the data would be a burden for clinicians and patients. It was felt that too great a focus on diet at the first visit might interfere with developing a good therapeutic rapport:

"There needs to be a balance between getting the information needed and establishing a collaborative working relationship with the client - often too much probing into exact nature of diet/quantities etc can disrupt this." (25d91)

"It would be too overwhelming, and I think that some people would end the session, and go to the supermarket or shop on the way home, and buy as many 'bad' foods and drink as they want, as they are already a 'failure', because they are not eating enough fibre, protein, plant based foods etc." (d5efb)

Respondent ddcea commented that when starting weight management treatment, patients may lack the knowledge of what is required in terms of daily nutritional intake. To keep things simple, 1e5f5 proposed just focusing on daily calories, to avoid over-medicalising weight loss.

However, c4b29 did remark that daily calorie intake:

"…..may not be useful as a measure. If you are fat you will consume around 400 extra calories as that body is harder to heave around and other metabolic functions like digestion and heat loss are less efficient. So having a `standard' will be different depending on your weight."

For d7ff7, using a 24 hour recall period may lead to inaccurate accounts, especially if someone knows they are being monitored. This notion of it being difficult to gather accurate data on what patients consume was raised by a number of participants, who thought that individuals may not recall or give a precise account of what they had eaten. Consequently, 5035d suggested that it might be better to get information about patients' dietary goals from being part of a weight management service. Using mobile phone apps to record dietary intake could be one way to help with recall, according to c2e0e. Finally, 4ec71 believed that information about alcohol consumption could be important to gather not just due to calories, but also to identify issues of dependency.

Other areas suggested

- Frequency of consumption of confectionery
- Patients' dietary goal(s)

Comorbidities (at baseline)

Some participants regarded comorbidities as important to measure if the weight loss intervention aimed to see an improvement in them, but scored lower if this was not seen to be the case. A commissioner said they would be interested in seeing improvements in these areas because they are linked to national priorities (e.g. diabetes, cardiovascular disease and high blood pressure). However, responses from members of the public implied that they did not really understand the rationale behind asking about these outcomes. For instance, 1e5f5 wrote:

"I have answered 4 to all of these. I am really unsure about the ethics of what could be seen as overly intrusive questions and also implying direct casual links to conditions that may have nothing to do with weight. For example, I have chronic kidney disease - due to Lupus not weight and would find it hard if someone was implying it was 'all because of being overweight'."

Some healthcare professionals worried that collecting data on comorbidities over-medicalised obesity, and took the focus away from lifestyle changes. Concern was also expressed by 8b0d4 that the process of gathering information on these comorbidities would put extra pressure on clinicians in weight management services.

Respondent 0764f stated that the questions about polycystic ovaries and infertility applied only to women and, therefore, gave a score in the middle of the rating scale. Another member of the public commented:

"PCOS - as this affects such a large proportion of women I think it is imperative this is measured. Especially as the Royal College of Gynaecology are recommending weight loss surgery as frontline treatment for women with long term obesity difficulties." (ff8ee)

<u>Other areas suggested</u>

- Participants with vitamin d deficiency

- Previous bariatric surgery

Lifestyle behaviours (at baseline)

Participant 4ec71 decided to rate these outcomes 9, because they may influence whether someone can have bariatric surgery in the future. Conversely, 8b0d4 thought that as outcomes were about assessing the effect of weight management services, not offering a public health profile of participants, they should be scored 1. Likewise, df6f8 scored them 6, explaining:

"Would tend to see these as part of routine clinical assessment rather than outcome measure."

It was proposed that it might be difficult for patients to be open about addictive behaviours and a risk of individuals feeling judged. However, ff8ee deemed that these were problems that needed to be known about because they could be exacerbated during weight loss attempts, and may be *"indicative of an underlying psychiatric need"*. Although d5efb rated these outcomes 8, they noted that people who smoked required education and support, but would make an individual choice about stopping.

Psychological factors (at baseline)

In response to this section, considerable concern was expressed about overburdening clinicians and patients. It was noted that putting too many questionnaires in front of patients could get in the way of developing a rapport, and was seen by some as intrusive and addressing areas that patients may have never spoken about before. Some participants queried whether individuals would answer honestly about their mental health. Furthermore, it was noted that a service would have to know how to respond if suicidal thoughts were revealed. Measurement of these areas was not seen, by

certain participants, as important for assessing the effectiveness of weight management services, which were not set up to address psychological issues. Others felt it was important to consider these areas to redress the balance that favoured physical aspects of obesity, and because they could influence patients motivation:

“I think the above outcomes are extremely important and currently I believe them to be over-looked in comparison to the more ‘pressing’ issues of physical health. However emotional and psychological wellbeing I believe to be one of the main underlying issues with people experiencing long term obesity and there is a lack of support available to address this." (ff8ee)

“I think self-esteem and confidence is very important for these patients due to the impact on their likely success at making behavioural changes." (4ec71)

Quality of life was mentioned by a couple of participants as a cost-effective measure and a good overall way of considering well-being, although ab65c remarked that it depended on questionnaires used and their reliability. The same comment was made about depression and anxiety by some participants. Respondent c2e0e stated measuring things like social relationships and social support might be important, depending on the nature of the intervention. A member of the public struggled to make a judgment on some of these outcomes because of a lack of experience of them. Respondent c4b29 scored them all 1, stating it was obvious patients who were obese would experience unhappiness.

<u>Other areas suggested</u>

- Other measures of disordered eating as well as binge eating, night eating, secret eating, bulimia etc

Physical measurements (at follow-up/end of programme)

A continued concern was expressed by 7c978 about BMI not being appropriate for all and that waist circumference was not helpful. A focus on weight loss was called for by several individuals as the core remit of a weight management service, although c4b29 wrote, *"It is lives you need to change not kilograms."* Several participants regarded positive changes in physical areas as motivating for patients, as well as showing the effectiveness of an intervention.

Physical activity (at follow-up/end of programme)

As in other sections, low scores were given to a number of these items which people felt would be difficult to measure accurately. Due to this difficulty, c4b29 questioned whether it was worth the effort:

"All this recording takes time. I am repeating myself but the best use of time is figuring out why that person is fat in the first place and addressing these issues."

There was a suggestion from 3d9b3 that recording changes in physical activity levels could be used as a motivational tool for patients.

<u>Other areas suggested</u>

- Shortness of breath on exertion - a fitness test with a before and after measure

Diet (at follow-up/end of programme)

Doubt was expressed as to how easily or accurately information on diet could be recorded. For 7c978, it would be burdensome to do so. Respondent c4b29 scored some areas in this section very low because of the message they seemed to relay:

"I do despair that eggs, milk, cheese, still get a bad press and implicit in this question is we should be eating less of these."

Other areas suggested

How far personal dietary goals, set at initial visit, have been met

Comorbidities (at follow-up/end of programme)

Uncertainty was expressed by some people who scored these outcomes low because of the complexity associated with their onset and maintenance; it was noted that improvements in a condition could be due to a range of factors, not just weight loss:

"There might be changes in medication during the process such as starting statins or changing meds for T2D which would confound results." (7c978)

Respondent c4b29 was dismissive about the need to record this information (scoring them all 1), stating:

"These parameters will change if you become thinner so why bother to record them?"

Conversely, d5efb scored them all 9, stating they were important to patients who were obese:

"To potentially reverse some of these health issues, or at least get them under control, is worth more than gold."

Other areas suggested

- An accepted definition of diabetes remission (e.g. at post intervention and 12months post intervention, diabetic blood markers being used to describe a non diabetic state)

Lifestyle behaviours (at follow-up/end of programme)

A reason for giving low scores to outcomes in this section was because people felt that the aim of a weight management service was not to address these areas and, therefore, a change in them should not be expected. Even d5efb, who gave a score of 8 and said it would be useful to know this information, noted that it was unrealistic to anticipate that patients would address *"all of his/her demons at once".*

Psychological factors (at follow-up/end of programme)

Again, some people were concerned about overburdening patients with questionnaires and, therefore, suggested just assessing quality of life (QoL):

"I would just report QoL as recommended by NOO SEF." (7c978)

In contrast, d5efb felt it was important to assess all areas because losing weight brings contentment and happiness in a thinner body, helping to resolve psychological problems. Others suggested that it

may be useful to gather this information to indicate how to shape a programme to best support a patient. However, a couple of participants queried whether it was necessary to assess things like autism and personality disorder, which were not going to change following weight loss.

Programme specific outcomes

There was some uncertainty expressed here about where data for some of the outcomes would be located/stored (e.g. reach, representativeness). In addition, a commissioner who scored the reach outcome 5 explained that:

"There are such a high number of residents who would be eligible for a weight management service, that looking at the reach as a percentage of the eligible populations isn't that helpful a study as it will be a very small percentage." (d7ff7)

Cost effectiveness was scored relatively low by some participants because it was not something services felt able to measure (lack of expertise in this area). However, others rated it high, arguing that such information was required to support the case for NHS funding. Respondent 18738 scored all these outcomes relatively high, stating:

"Far too often we only see the results of completers and not the bigger picture."

Yet some participants remarked that patients would not give the reason for dropping out of a service. Information on referral was said to help with understanding the patient pathway.

<u>Other areas suggested</u>

- Uptake (number and characteristics of those invited/referred vs those who actually went)

- Outcomes relating to anti-obesity medication, to see whether its effectiveness warrants the NHS using it

Length of follow-up

Some participants remarked that it would be difficult to engage patients in too many assessment points. Assessment at 12 months seemed popular because longer than that was a challenge. Respondent 4ec71 noted that most programmes lasted for 3 months and that 12 months was reasonable for showing a long-term effect. Conversely, 1e5f5 stated that earlier assessments were important because this is when people might need most support. Several individuals made this link between follow-up and keeping people motivated to lose weight; for picking up if someone had *"fallen off the wagon" (d5efb)* and for reinforcing people's commitment to the programme. Respondent 5f9c2 noted that services often state that after two years down the line people's behaviours will be influenced by a range of other variables. While for 4ec71, assessment at one month was too early to see behaviour change. Likewise, c4b29 scored most of the time points low because although regarding follow-up as helpful, this person felt sustained weight loss called for resiliency which was not just affected by attending a service. It was noted that the length and type of intervention could influence when data should be collected. The complexity in time points was also reflected in this comment from f9a2d:

"...weight requires ongoing support and management, moving from a weight loss phase to prevention of relapse and weight loss maintenance. Many community weight management services are also set up to provide this long term support intervention and as such do not have a set programme length, allowing the option of clients/patients being able to return to the service at any given point for longer term support."

Supporting Information 5. Stage 1 (outcome selection), round 2 Delphi questionnaire as it appeared to participants.

Question ID: 133

1.

Outcomes Using Information from First Visit

There now follows a list of potential outcomes presented under 7 categories (Demographics, Physical Measurements, Physical Activity, Diet, Comorbidities, Lifestyle Behaviours and Psychological Factors). Please rate how important you think it is for weight management services to measure and report a given outcome. By reporting the following outcomes, a weight management service will be showing what type of people are referred to and start the programme. When these outcomes are reported, it is likely that averages or percentages will be given.

For each item, you will be shown the median (the point where 50% (or half) of the panel's responses were above and half were below). You will also be shown your answer from the previous round.

Your answer last time: #demo

Question ID: 134

Demographics (At First Visit)

Your answer last time: #demo

Question ID: 135

1.1. * Age
How old participants are/the age (in years) of participants.

Median rating for the panel: 8

Your answer last time: #demo

Not at all important								Very important
1	2	3	4	5	6	7	8	9

Question ID: 136

1.2. * Gender
How participants identify themselves with regard to being male, female or non-binary/third gender.

Median rating for the panel: 7.5

Your answer last time: #demo

Not at all important								Very important
1	2	3	4	5	6	7	8	9

Question ID: 140

1.3. * Ethnicity
The social group with common national and cultural tradition that participants identify as belonging to e.g. White/White British, Asian/Asian British, Black/African/Caribbean/Black British.

Median rating for the panel: 7

Your answer last time: #demo

Not at all important								Very important
1	2	3	4	5	6	7	8	9

Question ID: 142

1.4. * Physical Disability
Whether participants have a recognised physical disadvantage or handicap.

Median rating for the panel: 7

Your answer last time: #demo

Not at all important								Very important
1	2	3	4	5	6	7	8	9

Question ID: 143

1.5. * Learning Disability
Whether participants have a recognised mental/cognitive disadvantage or handicap.

Median rating for the panel: 7

Your answer last time: #demo

Not at all important								Very important
1	2	3	4	5	6	7	8	9

Question ID: 144

1.6. * Deprivation Category
A measure of the level of poverty in the area in which the participant lives.

Median rating for the panel: 7

Your answer last time: #demo

Not at all important								Very important
1	2	3	4	5	6	7	8	9

Question ID: 149

1.7. * Family History of Obesity
Whether participants have close family members who have been identified as being overweight/obese.

Median rating for the panel: 8

Your answer last time: #demo

Not at all important								Very important
1	2	3	4	5	6	7	8	9

Question ID: 150

1.8. * Weight Loss History
Whether participants have made previous attempts to lose weight and, if so, whether these attempts were successful. If attempts were successful, how much weight was lost and how was the weight loss achieved.

Median rating for the panel: 7

Your answer last time: #demo

Not at all important								Very important
1	2	3	4	5	6	7	8	9

Question ID: 151

1.9. You may use this space to elaborate on your views in relation to any of the above outcomes. Please note to which of the above outcomes your comment(s) relatesYour answer last time: #demo

Maximum length: 5000 characters. Characters left: 5000

Question ID: 154

Physical Measurements (At First Visit)Your answer last time: #demo

Reminder: For each item please rate how important you think it is for weight management services to measure and report a given outcome. For each outcome listed below, please use the 9-point scale to rate importance.

Question ID: 155

1.10. * Weight
The measurement of how heavy a participant is in kilograms (kg) or stones and pounds.

Median rating for the panel: 9

Your answer last time: #demo

Not at all important								Very important
1	2	3	4	5	6	7	8	9

Question ID: 156

1.11. * Body Mass Index (BMI)
An approximate measure of whether a participant is overweight or underweight, calculated by dividing their weight in kilograms by the square of their height in metres.

Median rating for the panel: 9

Your answer last time: #demo

Not at all important								Very important
1	2	3	4	5	6	7	8	9

Question ID: 157

1.12. * Waist Circumference
A measurement taken around the abdomen at the level of the umbilicus (belly button). Health experts use waist circumference to screen patients for possible weight-related health problems.

Median rating for the panel: 7

Your answer last time: #demo

Not at all important								Very important
1	2	3	4	5	6	7	8	9

Question ID: 159

1.13. * Blood Pressure
The pressure of blood in the arteries, the vessels that carry blood from the heart to the rest of the body. A certain amount of pressure is required to get the blood around the body but consistently high blood pressure increases the risk of cardiovascular disease.

Median rating for the panel: 7

Your answer last time: #demo

Not at all important								Very important
1	2	3	4	5	6	7	8	9

Question ID: 161

1.14. * Fat Mass/Body Composition
The portion of the human body that is composed strictly of fat or muscle.

Median rating for the panel: 7

Your answer last time: #demo

Not at all important								Very important
1	2	3	4	5	6	7	8	9

Question ID: 342

1.15. * Neck Circumference
The measurement of the circumference of a participant's neck. Increased neck circumference has been shown to be a useful initial screening tool for overweight/obesity.

This is a new item for this round and has no previous rating.

Your answer last time: #demo

Not at all important								Very important
1	2	3	4	5	6	7	8	9

Question ID: 162

1.16. You may use this space to elaborate on your views in relation to any of the above outcomes. Please note to which of the above outcomes your comment(s) relates.Your answer last time: #demo

Maximum length: 5000 characters. Characters left: 5000

Question ID: 165

Physical Activity (At First Visit)Your answer last time: #demo

Reminder: For each item please rate how important you think it is for weight management services to measure and report a given outcome. For each outcome listed below, please use the 9-point scale to rate importance.

Question ID: 335

1.17. * Non Leisure Time Physical Activity
Physical activity performed during a participant's regular occupation, housework or transportation.

Median rating for the panel: 7

Your answer last time: #demo

Not at all important								Very important
1	2	3	4	5	6	7	8	9

Question ID: 166

1.18. * Leisure Time Physical Activity
Physical activity performed during exercise, recreation or any time other than during a participant's regular occupation, housework, or transportation.

Median rating for the panel: 7

Your answer last time: #demo

Not at all important								Very important
1	2	3	4	5	6	7	8	9

Question ID: 167

1.19. * Fitness
The ability of participants to undertake sustained physical exertion without undue breathlessness.

Median rating for the panel: 7

Your answer last time: #demo

Not at all important								Very important
1	2	3	4	5	6	7	8	9

Question ID: 168

1.20. * Sedentary Time
Time, during waking hours, when there is little or no physical activity. Examples of sedentary behaviours include reading, watching television, playing video games and sitting at a computer. A sedentary lifestyle may contribute to weight gain.

Median rating for the panel: 7

Your answer last time: #demo

Not at all important								Very important
1	2	3	4	5	6	7	8	9

Question ID: 169

1.21. You may use this space to elaborate on your views in relation to any of the above outcomes. Please note to which of the above outcomes your comment(s) relates.Your answer last time: #demo

Maximum length: 5000 characters. Characters left: 5000

Question ID: 172

Diet (At First Visit)Your answer last time: #demo

Reminder: For each item please rate how important you think it is for weight management services to measure and report a given outcome. For each outcome listed below, please use the 9-point scale to rate importance.

Question ID: 173

1.22. * Daily Calorie Consumption
The amount of fuel (food), measured in kilocalories (kcal), that a participant consumes (by eating or drinking) in a twenty four hour period in order to provide their body with energy to perform bodily processes e.g. maintenance of body temperature, movement of muscles etc. To maintain a stable weight, the energy going into the body must be the same as the energy being used up through normal body functions and physical activity. An important part of a healthy diet is eating the right amount of calories; balancing the energy going into the body with the energy being used.

Median rating for the panel: 7

Your answer last time: #demo

Not at all important								Very important
1	2	3	4	5	6	7	8	9

Question ID: 179

1.23. * Daily Fruit & Vegetable Intake
The number of portions of fruit and vegetables a participant consumes in a twenty four hour period. Guidelines recommend that 5 portions of fruit and vegetables are consumed every day as part of a healthy diet.

Median rating for the panel: 7

Your answer last time: #demo

Not at all important								Very important
1	2	3	4	5	6	7	8	9

Question ID: 181

1.24. * Daily Alcohol Consumption
The number of units of alcohol a participant consumes in a twenty four hour period. Units are a simple way of expressing the quantity of pure alcohol in a drink.

Median rating for the panel: 7

Your answer last time: #demo

Not at all important								Very important
1	2	3	4	5	6	7	8	9

Question ID: 182

1.25. You may use this space to elaborate on your views in relation to any of the above outcomes. Please note to which of the above outcomes your comment(s) relates.Your answer last time: #demo

Maximum length: 5000 characters. Characters left: 5000

Question ID: 185

Comorbidities (Other Diseases/ Conditions A Participant May Have*)

The final selected outcome will be a simple percentage of participants with the condition or may be specific blood test results e.g. blood cholesterol).

*N.B. Psychological/mental health conditions are not included here but in a subsequent section.

Your answer last time: #demo

Reminder: For each item please rate how important you think it is for weight management services to measure and report a given outcome. For each outcome listed below, please use the 9-point scale to rate importance.

Question ID: 186

1.26. * Cardiovascular Risk
A measure of how likely participants are to develop cardiovascular disease, including heart disease and stroke.

Median rating for the panel: 7

Your answer last time: #demo

Not at all important | Very important

1	2	3	4	5	6	7	8	9

Question ID: 187

1.27. * Diabetes Status
Whether a participant has diabetes, a condition which occurs when the body doesn't produce enough insulin to function properly, or the body's cells don't react to insulin. This means glucose stays in the blood and isn't used as fuel for energy. Type 2 diabetes is often associated with obesity and an increased risk of developing cardiovascular disease.

Median rating for the panel: 8

Your answer last time: #demo

Not at all important | Very important

1	2	3	4	5	6	7	8	9

Question ID: 188

1.28. * High Future Risk of Diabetes (Impaired Fasting Glucose, Impaired Glucose Tolerance, Raised HbA1c Levels)
Whether measures of the amount of glucose in a participant's blood suggests he/she is likely to develop type 2 diabetes in the future.

Median rating for the panel: 7.5

Your answer last time: #demo

Not at all important | Very important

1	2	3	4	5	6	7	8	9

Question ID: 189

1.29. * High Blood Pressure
Whether a participant has high blood pressure. High blood pressure increases the risk of developing cardiovascular disease.

Median rating for the panel: 7.5

Your answer last time: #demo

Not at all important | Very important

1	2	3	4	5	6	7	8	9

Question ID: 190

1.30. * High Cholesterol/Lipids
A measure of whether a participant has an abnormal amount of fat and/or cholesterol, known as lipids, in their blood (also called dyslipidaemia). Being overweight can increase the likelihood of developing dyslipidaemia. Dyslipidaemia is associated with increased risk of developing cardiovascular disease.

Median rating for the panel: 7

Your answer last time: #demo

Not at all important | Very important

1	2	3	4	5	6	7	8	9

Question ID: 191

1.31. * Obstructive Sleep Apnoea
Whether participants have obstructive sleep apnoea, a condition wherein the walls of the throat relax and narrow during sleep, interrupting normal breathing. Being overweight can increase the risk of developing obstructive sleep apnoea.

Median rating for the panel: 7

Your answer last time: #demo

Not at all important | Very important

1	2	3	4	5	6	7	8	9

Question ID: 192

1.32. * Osteoarthritis
Whether participants have osteoarthritis, a condition that causes joints to become painful and stiff. Being overweight puts excess strain on the joints and can therefore increase the risk of developing osteoarthritis.

Median rating for the panel: 7

Your answer last time: #demo

Not at all important | Very important

1	2	3	4	5	6	7	8	9

Question ID: 193

1.33. * Chronic Back Pain
Whether participants have back pain which has lasted more than 12 weeks. Being overweight can increase the risk of developing back pain.

Median rating for the panel: 7

Your answer last time: #demo

Not at all important								Very important
1	2	3	4	5	6	7	8	9

Question ID: 195

1.34. * Chronic Kidney Disease
Whether participants have experienced progressive loss in kidney function over a period of months or years. High blood pressure, high cholesterol and diabetes may cause chronic kidney disease.

Median rating for the panel: 7

Your answer last time: #demo

Not at all important								Very important
1	2	3	4	5	6	7	8	9

Question ID: 196

1.35. * Non Alcoholic Fatty Liver Disease (NAFLD)
Whether participants have a condition caused by a build-up of fat in the liver. NAFLD is usually seen in people who are overweight or obese.

Median rating for the panel: 7

Your answer last time: #demo

Not at all important								Very important
1	2	3	4	5	6	7	8	9

Question ID: 197

1.36. * Mobility Issues
Whether participants are unable to move with ease and without restriction. Being overweight has been associated with restricted mobility.

Median rating for the panel: 8

Your answer last time: #demo

Not at all important								Very important
1	2	3	4	5	6	7	8	9

Question ID: 198

1.37. * Advised To Lose Weight Prior To Routine Surgery
Whether a participant's GP has recommended he/she lose weight before having a standard surgical procedure to reduce the risk of suffering complications while under general anaesthetic.

Median rating for the panel: 7

Your answer last time: #demo

Not at all important								Very important
1	2	3	4	5	6	7	8	9

Question ID: 199

1.38. * Polycystic Ovary Syndrome (women only)
Whether participants have a condition characterised by the accumulation of numerous cysts (fluid-filled sacs) on the ovaries and associated with high male hormone levels, absent ovulation and other metabolic disturbances. Being overweight can worsen symptoms of polycystic ovary syndrome.

Median rating for the panel: 7

Your answer last time: #demo

Not at all important								Very important
1	2	3	4	5	6	7	8	9

Question ID: 201

1.39. * Asthma
Whether participants have asthma, a lung condition which causes occasional breathing difficulties. Being overweight is known to worsen asthma symptoms.

Median rating for the panel: 7

Your answer last time: #demo

Not at all important								Very important
1	2	3	4	5	6	7	8	9

Question ID: 339

1.40. * Other Health Conditions Requiring A Specialist Diet
Whether participants have a health condition (other than coeliac disease) which means they must follow a particular diet e.g. kidney disease, metabolic disorders etc.

Median rating for the panel: 7

Your answer last time: #demo

Not at all important | Very important

1	2	3	4	5	6	7	8	9

Question ID: 344

1.41. * Overall Measure of Comorbidity
Using a standard scale, a measurement of how affected a participant is overall by the presence of conditions, diseases or disorders they have in addition to overweight/obesity.

This is a new item for this round and has no previous rating.

Your answer last time: #demo

Not at all important | Very important

1	2	3	4	5	6	7	8	9

Question ID: 202

1.42. You may use this space to elaborate on your views in relation to any of the above outcomes. Please note to which of the above outcomes your comment(s) relates.Your answer last time: #demo

Maximum length: 5000 characters. Characters left: 5000

Question ID: 205

Lifestyle Behaviours (At Baseline)Your answer last time: #demo

Reminder: For each item please rate how important you think it is for weight management services to measure and report a given outcome. For each outcome listed below, please use the 9-point scale to rate importance.

Question ID: 206

1.43. * Smoking Status
Whether participants smoke tobacco regularly. Smoking can increase the risk of developing cardiovascular disease.

Median rating for the panel: 7

Your answer last time: #demo

Not at all important | Very important

1	2	3	4	5	6	7	8	9

Question ID: 207

1.44. * Other Addictive Behaviour
Whether participants have a dependency on a particular substance (other than food) or behaviour e.g. alcohol, narcotics, sex etc.

Median rating for the panel: 7

Your answer last time: #demo

Not at all important | Very important

1	2	3	4	5	6	7	8	9

Question ID: 208

1.45. You may use this space to elaborate on your views in relation to any of the above outcomes. Please note to which of the above outcomes your comment(s) relates.Your answer last time: #demo

Maximum length: 5000 characters. Characters left: 5000

Question ID: 211

Psychological Factors (At Baseline)

It is well known that people who are overweight or obese can have a range of psychological complications which may be either a cause or consequence of their weight.

Your answer last time: #demo

Reminder: For each item please rate how important you think it is for weight management services to measure and report a given outcome. For each outcome listed below, please use the 9-point scale to rate importance.

Question ID: 212

1.46. * Self Esteem
How participants feel about their own worth.

Median rating for the panel: 7

Your answer last time: #demo

Not at all important								Very important
1	2	3	4	5	6	7	8	9

Question ID: 213

1.47. * Self Confidence
How participants feel about their own abilities.

Median rating for the panel: 7

Your answer last time: #demo

Not at all important								Very important
1	2	3	4	5	6	7	8	9

Question ID: 214

1.48. * Importance of Weight Loss
How important participants feel it is for them to lose weight.

Median rating for the panel: 8

Your answer last time: #demo

Not at all important								Very important
1	2	3	4	5	6	7	8	9

Question ID: 215

1.49. * Confidence in Ability to Lose Weight
How sure participants feel that they will be able to lose weight.

Median rating for the panel: 7.5

Your answer last time: #demo

Not at all important								Very important
1	2	3	4	5	6	7	8	9

Question ID: 216

1.50. * Body Image
A participant's perception of how their own body looks and how attractive it is.

Median rating for the panel: 7

Your answer last time: #demo

Not at all important								Very important
1	2	3	4	5	6	7	8	9

Question ID: 217

1.51. * Depression
Whether a participant suffers from a mental illness characterised by a profound and persistent feeling of sadness or despair and/or a loss of interest in things that once were pleasurable.

Median rating for the panel: 8

Your answer last time: #demo

Not at all important								Very important
1	2	3	4	5	6	7	8	9

Question ID: 218

1.52. * Anxiety
Whether a participant suffers from a disorder characterised by nervousness, fear, apprehension and worrying.

Median rating for the panel: 7

Your answer last time: #demo

Not at all important								Very important
1	2	3	4	5	6	7	8	9

Question ID: 219

1.53. * Suicidal Thoughts
Whether a participant has thoughts about killing himself/herself.

Median rating for the panel: 7

Your answer last time: #demo

Not at all important | Very important

1	2	3	4	5	6	7	8	9

Question ID: 220

1.54. * Quality of Life (QoL) Score
A measure of the general well-being of participants.

Median rating for the panel: 8

Your answer last time: #demo

Not at all important | Very important

1	2	3	4	5	6	7	8	9

Question ID: 221

1.55. * Overall Quality of Sleep
How well a participant sleeps based on how tired they feel when waking and throughout the day, how well rested and relaxed they feel on waking, the number of times they awake during the night etc.

Median rating for the panel: 7

Your answer last time: #demo

Not at all important | Very important

1	2	3	4	5	6	7	8	9

Question ID: 225

1.56. * Binge Eating Disorder
Whether participants have an illness which causes them to overeat on a regular basis.

Median rating for the panel: 8

Your answer last time: #demo

Not at all important | Very important

1	2	3	4	5	6	7	8	9

Question ID: 226

1.57. * Autism
Whether participants have autism, a lifelong, developmental disability which affects how a person communicates with and relates to other people, and how they experience the world around them.

Median rating for the panel: 7

Your answer last time: #demo

Not at all important | Very important

1	2	3	4	5	6	7	8	9

Question ID: 227

1.58. * Personality Disorders
Whether participants have conditions wherein they differ significantly from an average person, in terms of how they think, perceive, feel or relate to others.

Median rating for the panel: 7

Your answer last time: #demo

Not at all important | Very important

1	2	3	4	5	6	7	8	9

Question ID: 347

1.59. * Disordered Eating
Disturbed and unhealthy eating patterns including, secret eating, night eating etc.

This is a new item for this round and has no previous rating.

Your answer last time: #demo

Not at all important | Very important

1	2	3	4	5	6	7	8	9

Question ID: 228

1.60. You may use this space to elaborate on your views in relation to any of the above outcomes. Please note to which of the above outcomes your comment(s) relates.Your answer last time: #demo

Maximum length: 5000 characters. Characters left: 5000

Question ID: 231

2.

Outcomes Using Information from Follow-up Visits and The End of The Programme

There now follows a list of potential outcomes presented under 7 categories (Demographics, Physical Measurements, Physical Activity, Diet, Comorbidities, Lifestyle Behaviours and Psychological Factors). Please rate how important you think it is for weight management services to measure and report a given outcome.

By reporting the following outcomes, a weight management service will be showing what effect the programme has had on participants. When these outcomes are reported, it is likely that averages or percentages will be given.

For each item, you will be shown the median (the point where 50% (or half) of the panel's responses were above and half were below). You will also be shown your answer from the previous round.

Your answer last time: #demo

Question ID: 232

Change in Physical Measurements (At Follow Up/ End of Programme)Your answer last time: #demo

Reminder: For each item please rate how important you think it is for weight management services to measure and report a given outcome. For each outcome listed below, please use the 9-point scale to rate importance.

Question ID: 233

2.1. * Weight
The measurement of how heavy a participant is in kilograms (kg) or stones and pounds.

Median rating for the panel: 9

Your answer last time: #demo

Not at all important | Very important

1	2	3	4	5	6	7	8	9

Question ID: 234

2.2. * Body Mass Index (BMI)
An approximate measure of whether a participant is overweight or underweight, calculated by dividing their weight in kilograms by the square of their height in metres.

Median rating for the panel: 9

Your answer last time: #demo

Not at all important | Very important

1	2	3	4	5	6	7	8	9

Question ID: 235

2.3. * Waist Circumference
A measurement taken around the abdomen at the level of the umbilicus (belly button). Health experts use waist circumference to screen patients for possible weight-related health problems.

Median rating for the panel: 7

Your answer last time: #demo

Not at all important | Very important

1	2	3	4	5	6	7	8	9

Question ID: 236

2.4. * Waist to Hip Ratio
An indicator of health and the risk of developing serious health conditions, obtained by calculating the ratio of a participant's waist circumference to their hip circumference.

Median rating for the panel: 7

Your answer last time: #demo

Not at all important | Very important

1	2	3	4	5	6	7	8	9

Question ID: 237

2.5. * Blood Pressure
The pressure of blood in the arteries, the vessels that carry blood from the heart to the rest of the body. A certain amount of pressure is required to get the blood around the body but consistently high blood pressure increases the risk of cardiovascular disease.

Median rating for the panel: 7

Your answer last time: #demo

Not at all important | Very important

1	2	3	4	5	6	7	8	9

Question ID: 239

2.6. * Fat Mass/Body Composition
The portion of the human body that is composed strictly of fat or muscle.

Median rating for the panel: 7

Your answer last time: #demo

Not at all important Very important

1	2	3	4	5	6	7	8	9

Question ID: 343

2.7. * Neck Circumference
The measurement of the circumference of a participant's neck. Increased neck circumference has been shown to be a useful initial screening tool for overweight/obesity.

This is a new item for this round and has no previous rating.

Your answer last time: #demo

Not at all important Very important

1	2	3	4	5	6	7	8	9

Question ID: 346

2.8. * Self Reported Reduction in Clothes Size
Whether a participant is wearing clothes in a smaller size due to weight loss.

This is a new item for this round and has no previous rating.

Your answer last time: #demo

Not at all important Very important

1	2	3	4	5	6	7	8	9

Question ID: 240

2.9. You may use this space to elaborate on your views in relation to any of the above outcomes. Please note to which of the above outcomes your comment(s) relates.Your answer last time: #demo

Maximum length: 5000 characters. Characters left: 5000

Question ID: 243

Change in Physical Activity (At Follow Up/ End of Programme)Your answer last time: #demo

Reminder: For each item please rate how important you think it is for weight management services to measure and report a given outcome. For each outcome listed below, please use the 9-point scale to rate importance.

Question ID: 341

2.10. * Non Leisure Time Physical Activity
Physical activity performed during a participant's regular occupation, housework or transportation.

Median rating for the panel: 7

Your answer last time: #demo

Not at all important Very important

1	2	3	4	5	6	7	8	9

Question ID: 244

2.11. * Leisure Time Physical Activity
Physical activity performed during exercise, recreation or any time other than during a participant's regular occupation, housework, or transportation.

Median rating for the panel: 7

Your answer last time: #demo

Not at all important Very important

1	2	3	4	5	6	7	8	9

Question ID: 245

2.12. * Fitness
The ability of participants to undertake sustained physical exertion without undue breathlessness.

Median rating for the panel: 7

Your answer last time: #demo

Not at all important								Very important
1	2	3	4	5	6	7	8	9

Question ID: 246

2.13. * Sedentary Time
Time, during waking hours, when there is little or no physical activity. Examples of sedentary behaviours include reading, watching television, playing video games and sitting at a computer. A sedentary lifestyle may contribute to weight gain.

Median rating for the panel: 7

Your answer last time: #demo

Not at all important								Very important
1	2	3	4	5	6	7	8	9

Question ID: 247

2.14. You may use this space to elaborate on your views in relation to any of the above outcomes. Please note to which of the above outcomes your comment(s) relates.Your answer last time: #demo

Maximum length: 5000 characters. Characters left: 5000

Question ID: 250

Change in Diet (At Follow Up/ End of Programme)Your answer last time: #demo

Reminder: For each item please rate how important you think it is for weight management services to measure and report a given outcome. For each outcome listed below, please use the 9-point scale to rate importance.

Question ID: 251

2.15. * Daily Calorie Consumption
The amount of fuel (food), measured in kilocalories (kcal), that a participant consumes (by eating or drinking) in a twenty four hour period in order to provide their body with energy to perform bodily processes e.g. maintenance of body temperature, movement of muscles etc. To maintain a stable weight, the energy going into the body must be the same as the energy being used up through normal body functions and physical activity. An important part of a healthy diet is eating the right amount of calories; balancing the energy going into the body with the energy being used.

Median rating for the panel: 7

Your answer last time: #demo

Not at all important								Very important
1	2	3	4	5	6	7	8	9

Question ID: 256

2.16. * Daily Free Sugar Intake
The amount of free sugar a participant consumes in a twenty four hour period. Free sugar is defined by the World Health Organization as all monosaccharides and disaccharides added to foods by the manufacturer, cook, or consumer, plus sugars naturally present in honey, syrups, and fruit juices. It is used to distinguish between the sugars that are naturally present in fully unrefined carbohydrates such as brown rice, whole wheat pasta, fruit, etc. and those sugars (or carbohydrates) that have been, to some extent, refined (normally by humans but sometimes by animals, such as the free sugars present in honey). Too much free sugar can cause weight gain and increase the risk of type 2 diabetes.

Median rating for the panel: 7

Your answer last time: #demo

Not at all important								Very important
1	2	3	4	5	6	7	8	9

Question ID: 257

2.17. * Daily Fruit & Vegetable Intake
The number of portions of fruit and vegetables a participant consumes in a twenty four hour period. Guidelines recommend that 5 portions of fruit and vegetables are consumed every day as part of a healthy diet.

Median rating for the panel: 7

Your answer last time: #demo

Not at all important								Very important
1	2	3	4	5	6	7	8	9

Question ID: 259

2.18. * Daily Alcohol Consumption
The number of units of alcohol a participant consumes in a twenty four hour period. Units are a simple way of expressing the quantity of pure alcohol in a drink.

Median rating for the panel: 7

Your answer last time: #demo

Not at all important								Very important
1	2	3	4	5	6	7	8	9

Question ID: 260

2.19. You may use this space to elaborate on your views in relation to any of the above outcomes. Please note to which of the above outcomes your comment(s) relates.Your answer last time: #demo

Maximum length: 5000 characters. Characters left: 5000

Question ID: 263

Changes in Comorbidities (Other Diseases/ Conditions A Participant May Have*)

The final selected outcome will be a simple percentage of participants with the condition and the change, or may be a change in a specific blood test result (e.g. blood cholesterol).

*N.B. Psychological/mental health conditions are not included here but in a subsequent section.

Your answer last time: #demo

Reminder: For each item please rate how important you think it is for weight management services to measure and report a given outcome. For each outcome listed below, please use the 9-point scale to rate importance.

Question ID: 264

2.20. * Cardiovascular Risk
A measure of how likely participants are to develop cardiovascular disease, including heart disease and stroke.

Median rating for the panel: 7

Your answer last time: #demo

Not at all important | Very important

1	2	3	4	5	6	7	8	9

Question ID: 265

2.21. * Diabetes Status
Whether a participant has diabetes, a condition which occurs when the body doesn't produce enough insulin to function properly, or the body's cells don't react to insulin. This means glucose stays in the blood and isn't used as fuel for energy. Type 2 diabetes is often associated with obesity and an increased risk of developing cardiovascular disease.

Median rating for the panel: 8

Your answer last time: #demo

Not at all important | Very important

1	2	3	4	5	6	7	8	9

Question ID: 266

2.22. * High Future Risk of Diabetes (Impaired Fasting Glucose, Impaired Glucose Tolerance, Raised HbA1c Levels)
Whether measures of the amount of glucose in a participant’s blood suggests he/she is likely to develop type 2 diabetes in the future.

Median rating for the panel: 7.5

Your answer last time: #demo

Not at all important | Very important

1	2	3	4	5	6	7	8	9

Question ID: 267

2.23. * High Blood Pressure
Whether a participant has high blood pressure. High blood pressure increases the risk of developing cardiovascular disease.

Median rating for the panel: 7.5

Your answer last time: #demo

Not at all important | Very important

1	2	3	4	5	6	7	8	9

Question ID: 268

2.24. * High Cholesterol/Lipids
A measure of whether a participant has an abnormal amount of fat and/or cholesterol, known as lipids, in their blood (also called dyslipidaemia). Being overweight can increase the likelihood of developing dyslipidaemia. Dyslipidaemia is associated with increased risk of developing cardiovascular disease.

Median rating for the panel: 7

Your answer last time: #demo

Not at all important | Very important

1	2	3	4	5	6	7	8	9

Question ID: 269

2.25. * Obstructive Sleep Apnoea
Whether participants have obstructive sleep apnoea, a condition wherein the walls of the throat relax and narrow during sleep, interrupting normal breathing. Being overweight can increase the risk of developing obstructive sleep apnoea.

Median rating for the panel: 7

Your answer last time: #demo

Not at all important								Very important
1	2	3	4	5	6	7	8	9

Question ID: 271

2.26. * Chronic Back Pain
Whether participants have back pain which has lasted more than 12 weeks. Being overweight can increase the risk of developing back pain.

Median rating for the panel: 7

Your answer last time: #demo

Not at all important								Very important
1	2	3	4	5	6	7	8	9

Question ID: 275

2.27. * Mobility Issues
Whether participants are unable to move with ease and without restriction. Being overweight has been associated with restricted mobility.

Median rating for the panel: 8

Your answer last time: #demo

Not at all important								Very important
1	2	3	4	5	6	7	8	9

Question ID: 345

2.28. * Overall Measure of Comorbidity
Using a standard scale, a measurement of how affected a participant is overall by the presence of conditions, diseases or disorders they have in addition to overweight/obesity.

This is a new item for this round and has no previous rating.

Your answer last time: #demo

Not at all important								Very important
1	2	3	4	5	6	7	8	9

Question ID: 280

2.29. You may use this space to elaborate on your views in relation to any of the above outcomes. Please note to which of the above outcomes your comment(s) relates.Your answer last time: #demo

Maximum length: 5000 characters. Characters left: 5000

Question ID: 283

Change in Lifestyle Behaviours (At Follow Up/End of Programme)Your answer last time: #demo

Reminder: For each item please rate how important you think it is for weight management services to measure and report a given outcome. For each outcome listed below, please use the 9-point scale to rate importance.

Question ID: 285

2.30. * Other Addictive Behaviour
Whether participants have a dependency on a particular substance (other than food) or behaviour e.g. alcohol, narcotics, sex etc.

Median rating for the panel: 7

Your answer last time: #demo

Not at all important								Very important
1	2	3	4	5	6	7	8	9

Question ID: 286

2.31. You may use this space to elaborate on your views in relation to any of the above outcomes. Please note to which of the above outcomes your comment(s) relates.Your answer last time: #demo

Maximum length: 5000 characters. Characters left: 5000

Question ID: 289

Changes in Psychological Factors (At Follow Up/End of Programme)

It is well known that people who are overweight or obese can have a range of psychological complications which may be either a cause or consequence of their weight.

Your answer last time: #demo

Reminder: For each item please rate how important you think it is for weight management services to measure and report a given outcome. For each outcome listed below, please use the 9-point scale to rate importance.

Question ID: 290

2.32. * Self Esteem
How participants feel about their own worth.

Median rating for the panel: 7

Your answer last time: #demo

Not at all important | Very important

1	2	3	4	5	6	7	8	9

Question ID: 291

2.33. * Self Confidence
How participants feel about their own abilities.

Median rating for the panel: 7

Your answer last time: #demo

Not at all important | Very important

1	2	3	4	5	6	7	8	9

Question ID: 292

2.34. * Importance of Weight Loss
How important participants feel it is for them to lose weight.

Median rating for the panel: 8

Your answer last time: #demo

Not at all important | Very important

1	2	3	4	5	6	7	8	9

Question ID: 293

2.35. * Confidence in Ability to Lose Weight
How sure participants feel that they will be able to lose weight.

Median rating for the panel: 7.5

Your answer last time: #demo

Not at all important | Very important

1	2	3	4	5	6	7	8	9

Question ID: 294

2.36. * Body Image
A participant's perception of how their own body looks and how attractive it is.

Median rating for the panel: 7

Your answer last time: #demo

Not at all important | Very important

1	2	3	4	5	6	7	8	9

Question ID: 295

2.37. * Depression
Whether a participant suffers from a mental illness characterised by a profound and persistent feeling of sadness or despair and/or a loss of interest in things that once were pleasurable.

Median rating for the panel: 8

Your answer last time: #demo

Not at all important | Very important

1	2	3	4	5	6	7	8	9

Question ID: 296

2.38. * Anxiety
Whether a participant suffers from a disorder characterised by nervousness, fear, apprehension and worrying.

Median rating for the panel: 7

Your answer last time: #demo

Not at all important | Very important

1	2	3	4	5	6	7	8	9

Question ID: 297

2.39. * Suicidal Thoughts
Whether a participant has thoughts about killing himself/herself.

Median rating for the panel: 7

Your answer last time: #demo

Not at all important Very important

1	2	3	4	5	6	7	8	9

Question ID: 298

2.40. * Quality of Life (QoL) Score
A measure of the general well-being of participants.

Median rating for the panel: 8

Your answer last time: #demo

Not at all important Very important

1	2	3	4	5	6	7	8	9

Question ID: 299

2.41. * Overall Quality of Sleep
How well a participant sleeps based on how tired they feel when waking and throughout the day, how well rested and relaxed they feel on waking, the number of times they awake during the night etc.

Median rating for the panel: 7

Your answer last time: #demo

Not at all important Very important

1	2	3	4	5	6	7	8	9

Question ID: 303

2.42. * Binge Eating Disorder
Whether participants have an illness which causes them to overeat on a regular basis.

Median rating for the panel: 8

Your answer last time: #demo

Not at all important Very important

1	2	3	4	5	6	7	8	9

Question ID: 348

2.43. * Disordered Eating
Disturbed and unhealthy eating patterns including, secret eating, night eating etc.

This is a new item for this round and has no previous rating.

Your answer last time: #demo

Not at all important Very important

1	2	3	4	5	6	7	8	9

Question ID: 306

2.44. You may use this space to elaborate on your views in relation to any of the above outcomes. Please note to which of the above outcomes your comment(s) relates.Your answer last time: #demo

Maximum length: 5000 characters. Characters left: 5000

Question ID: 309

Programme Specific Outcomes

These are outcomes related to how well used the service is and may be related to the service design, access and communication with GPs and hospitals.

Your answer last time: #demo

Reminder: For each item please rate how important you think it is for weight management services to measure and report a given outcome. For each outcome listed below, please use the 9-point scale to rate importance.

Question ID: 310

2.45. * Reach
The percentage of the eligible population (people who are overweight or obese within that particular geographical area) referred to the weight management service.

Median rating for the panel: 7

Your answer last time: #demo

Not at all important / Very important

1	2	3	4	5	6	7	8	9

Question ID: 311

2.46. * Representativeness
How representative of the entire eligible population (people who are overweight or obese within that particular geographical area) the people attending the weight management service are.

Median rating for the panel: 7.5

Your answer last time: #demo

Not at all important / Very important

1	2	3	4	5	6	7	8	9

Question ID: 312

2.47. * Attendance
How many people attended the weight management service.

Median rating for the panel: 9

Your answer last time: #demo

Not at all important / Very important

1	2	3	4	5	6	7	8	9

Question ID: 313

2.48. * Completion
How many people finished the entire weight management programme.

Median rating for the panel: 9

Your answer last time: #demo

Not at all important / Very important

1	2	3	4	5	6	7	8	9

Question ID: 314

2.49. * Cost Effectiveness
The value for money of the weight management service in terms of long term economic benefits to the NHS.

Median rating for the panel: 7.5

Your answer last time: #demo

Not at all important / Very important

1	2	3	4	5	6	7	8	9

Question ID: 315

2.50. * Sources of Referral
Where participants received their referral to the weight management service.

Median rating for the panel: 7

Your answer last time: #demo

Not at all important / Very important

1	2	3	4	5	6	7	8	9

Question ID: 316

2.51. * Repeat Referrals
The number of participants who were referred to the weight management service on more than one occasion.

Median rating for the panel: 7

Your answer last time: #demo

Not at all important / Very important

1	2	3	4	5	6	7	8	9

Question ID: 317

2.52. * Referral to Specialist Services
The number of participants referred to a specialist management service after failing to lose the required amount of weight via a lifestyle weight management programme or due to a condition needing specialist input.

Median rating for the panel: 7.5

Your answer last time: #demo

Not at all important | Very important

1	2	3	4	5	6	7	8	9

Question ID: 318

2.53. * Referral to Linked Services
The number of participants referred to services linked to weight management services e.g. smoking cessation services, psychiatric services etc.

Median rating for the panel: 7

Your answer last time: #demo

Not at all important | Very important

1	2	3	4	5	6	7	8	9

Question ID: 319

2.54. * Reason for Dropout
Why those participants who did not complete the programme failed to do so.

Median rating for the panel: 8

Your answer last time: #demo

Not at all important | Very important

1	2	3	4	5	6	7	8	9

Question ID: 320

2.55. * Participant Satisfaction
How happy/satisfied participants were with the weight loss service.

Median rating for the panel: 8

Your answer last time: #demo

Not at all important | Very important

1	2	3	4	5	6	7	8	9

Question ID: 321

2.56. * Adverse Events/Unintended Consequences
Whether participants suffered any unfortunate side effects as a result of attending the weight loss service.

Median rating for the panel: 8

Your answer last time: #demo

Not at all important | Very important

1	2	3	4	5	6	7	8	9

Question ID: 322

2.57. * Prescription of Anti-obesity Medication
The number of participants taking drugs to help reduce or control their weight.

Median rating for the panel: 7

Your answer last time: #demo

Not at all important | Very important

1	2	3	4	5	6	7	8	9

Question ID: 323

2.58. You may use this space to elaborate on your views in relation to any of the above outcomes. Please note to which of the above outcomes your comment(s) relates.Your answer last time: #demo

Maximum length: 5000 characters. Characters left: 5000

Question ID: 326

3.

Length of Follow Up

Please rate how important you think it is to report outcomes at the following time-points post completion of the weight loss programme.

For each item, you will be shown the median (the point where 50% (or half) of the panel's responses were above and half were below). You will also be shown your answer from the previous round.

Your answer last time: #demo

Reminder: For each item please rate how important you think it is for weight management services to measure and report a given outcome. For each outcome listed below, please use the 9-point scale to rate importance.

Question ID: 328

3.1. * 3 months

Median rating for the panel: 7

Your answer last time: #demo

Not at all important — Very important

1	2	3	4	5	6	7	8	9

Question ID: 329

3.2. * 6 months

Median rating for the panel: 8

Your answer last time: #demo

Not at all important — Very important

1	2	3	4	5	6	7	8	9

Question ID: 330

3.3. * 12 months

Median rating for the panel: 9

Your answer last time: #demo

Not at all important — Very important

1	2	3	4	5	6	7	8	9

Question ID: 331

3.4. * 18 months

Median rating for the panel: 7

Your answer last time: #demo

Not at all important — Very important

1	2	3	4	5	6	7	8	9

Question ID: 332

3.5. * 24 months

Median rating for the panel: 8

Your answer last time: #demo

Not at all important — Very important

1	2	3	4	5	6	7	8	9

Question ID: 334

3.6. You may use this space to elaborate on your views in relation to any of the above outcomes. Please note to which of the above outcomes your comment(s) relates.Your answer last time: #demo

Maximum length: 5000 characters. Characters left: 5000

Thank you, please click Save to finish.

Save

Save to complete later

Next Page >

Supporting Information 6. Stage 1 (outcome selection), round 2 Delphi qualitative analysis.

Ideas on first visit data collection

Areas still showing disagreement

There was still a degree of disagreement on all areas within this section. Qualitative feedback was provided to explain some, but not all, of this disparity. Family and weight loss history were seen as nice but not essential to measure a programme's performance by one academic (18738). Likewise, a member of the public (ddcea) wrote the same about ethnicity and socio-economic status. In contrast, academic 49a6b stated that a weight management programme's effectiveness is likely to differ by individuals' age, sex and level of deprivation and, therefore, needed to be taken into account. Discord on deprivation was evident between another two participants:

> *"I continue to think that a measure of deprivation is very important here; it would seem to be connected to educational attainment, and to personal, social, and financial resources to effect change" (8ba96, primary care staff).*

> *"Still feel that deprivation category not too relevant. Just because you live in an area of deprivation does not mean you cannot manage the resources or intellect to participate in a weight management programme" (3d9b3, public).*

One person (4ec71) suggested merging learning and physical disabilities into a single item if looking to reduce the number of areas measured.

Physical measurements (first visit)

In general, there was agreement about use of weight and body mass index (BMI) at baseline. In terms of other measures in this section, concern was expressed about their validity depending on how they are gathered and who gathers them as there may be inconsistency across different services and professionals. There was quite a bit of discord in terms of neck circumference. Some people felt that it was easy to measure and could be helpful in terms of anaesthetic and sleep apnoea. Others noted that it was not something generally taken in general practice with an academic (c1ffc) adding, *"I think its applicability to the general population is limited till we have more epidemiological data."* On this subject, another academic (18738) remarked that:

> *"Neck circumference may be more meaningful at the higher tier services. I think it would be onerous to ask tier 2 services to provide this data."*

This person also mentioned the need for services to have access to body composition scales to measure fat mass.

Physical activity (first visit)

There was a sense within the qualitative data that this may be interesting but not essential information to collect. Furthermore, it was noted that it relied upon individuals being honest or

accurate in recalling their activity levels. One academic (18738) queried whether increasing physical activity was expected or part of the remit of weight loss services:

"I suppose the question is, when assessing a weight management service, would you consider it not to be performing if someone had low physical activity but was losing weight? In truth, many effective weight management services don't have a huge impact on PA levels, but do achieve weight loss."

Diet (first visit)

In general, those working for weight management services tended to rate these areas lower than other groups. This seemed to relate to the complexity they associated with measuring these outcomes and the reliability of self-reports to do this. A focus on calories was seen as not applicable by a couple of participants:

"Slimming World does not refer to calories." (92f98, public)

"At the end of the day the vast majority of patients with obesity consume more calories than they spend so it's more important to focus on the triggers rather than the exact calorie intake." (c1ffc, academic)

In addition, some public respondents were not sure about measuring alcohol intake, a) because it might be seen as a chore for patients and b) it might not be related to body size:

"Many people do not drink and still have a weight problem and many people who drink a lot do not have a problem with weight control. The people I have met at weight management programs seem to already have an agenda they wish people to fit into instead of dealing with the people as individuals." (0e844, public)

Comorbidities (at baseline)

A couple of academics rated all comorbidities listed as important:

"These factors will either have an effect on the treatment strategy or are important outcomes of the intervention and hence are needed. The aim of the service is to improve health." (c1ffc)

"These clinical outcomes are relatively simple for services to measure and can provide important information on the health impact of a service. Some conditions may be more important at some levels of service than others - eg tier 2 vs tier 3." (18738)

A primary care staff respondent (8ba96) thought an overall measure of comorbidity sounded too subjective. Yet several weight management staff liked this as an easier way of collecting data and allowing for comparison across services. A focus on function rather than mobility was mentioned by 4ec71 who worked in primary care:

"Rather than mobility issues perhaps a measure of functional status might be a better option. Osteoarthritis I have scored lower as it's really how it affects mobility/function that I think is of more interest."

Lifestyle behaviours (at baseline)

There was variation within the ratings given for this section. However, few qualitative comments were written to unpick this diversity. In general, weight management staff seemed less positive about assessing these areas. This could be due to not seeing these as important compared to other outcomes, not collecting this information as standard and/or negative views about the accuracy of self-reports provided by patients.

Psychological factors (at baseline)

Public respondents tended to rate outcomes in this section relatively high, although one person in this group did not, writing:

> *"All answers can only be subjective and not absolute unless the patient has been pre-diagnosed with a particular disorder - ie autism. Many of the answers to these questions can change from day to day." (ddcea)*

This contrasted with lower ratings given by weight management staff who felt that services were not designed to tackle mental health problems. Some doubt was also expressed about the reliability of measures for assessing eating behaviours. One primary care worker had suggestions for simplifying this section:

> *"I think disordered eating is very important to include - could include binge eating - not sure needed separately?…Could suicidal thoughts be incorporated into depression score?...I think*

confidence to lose weight will be covered by self-confidence…Possibly consider self-efficacy rather than self-confidence. QoL must be included to allow cost effectiveness calculations." (4ec71)

Physical measurements (at follow-up/end of programme)

There was a sense of agreement about use of weight and BMI at this time point. One person who did not agree with this felt that pictures or a series of graphs showing a change in body shape might be preferable, especially for people who had gained muscle mass through exercising.

Self-reporting of clothes size received mixed ratings; the following comments give an idea of the difference people held in this respect:

"I feel that self-reporting on clothes size would be very inaccurate as there are no standard sizes in clothing." (f6c44, weight management staff)

"Self-reported clothes size is easy to measure and useful to show body shape changes." (25d91, weight management staff)

Fat mass and body composition were not highly rated, especially by weight management staff due to difficulties in measuring this accurately. Similarly, waist circumference and waist to hip ratio received mixed ratings. Again, weight management staff reported that it was difficult to measure and

depended on who performed this task. It was also noted that patients *"actually dislike having it measured more than they dislike weight/body fat or blood tests"* (18738, academic)

Physical activity (at follow-up/end of programme)

Weight management staff, in particular, rated these outcomes low. They wanted simple measures, perhaps asking about overall physical activity, and were concerned that reports provided by patients might be inaccurate. Likewise, a public respondent (ddcea) commented on the subjective nature of these outcomes. However, another public respondent (3d9b3) said that physical activity was an essential part of weight management programmes.

Diet (at follow-up/end of programme)

One participant (4ec71) stated that because not all these measures were listed at baseline, it was not necessarily correct to measure them at follow-up. Another person (c1ffc) felt that these outcomes were *"intermediary"*, reflecting changes in health and, therefore, gave them a low rating. Concern was also expressed about the subjective nature of responses:

> *"Participants are likely to misreport more at the onset of a programme and be more honest after participating in a programme and as such it is difficult measure any real dietary change." (f9a2d, weight management staff)*

Some people believed that alcohol intake was not relevant to all patients, and it was noted that weight management services were not alcohol reduction programmes. Conversely, one primary care

worker felt it was important, providing the following justification: *“Alcohol is more important due to issues with addiction transfer with some patients.”* (4ec71)

Comorbidities (at follow-up/end of programme)

Again, weight management staff tended to rate these outcomes relatively low; a lack of qualitative comments made it hard to decipher why this was the case, although the following remark gives some insight:

> *“We do not measure any of these on exiting - but they are probably quite useful. Also it will depend on how long the person has been in the programme - if it's only been a short time then there shouldn't be that much change.” (f6c44, weight management staff)*

The following participant felt that concern about illness could be a driver for change, so rated these outcomes highly:

> *“There's nothing like fear of disease to bring home the need for change and if participants can see in facts and figures that their own risk scores have gone down then this goes a long way to ensuring continued positive outcomes.” (ddcea, public)*

However, another public respondent was worried about the skills of those working in weight management services to address these outcomes:

> *“I believe a person more expert in the issues would be needed to decipher the findings and set a course of action.” (0e844, public)*

Lifestyle behaviours (at follow-up/end of programme)

This section asked about other addictive behaviours. The few comments that were made related to the potential for inaccuracy because it relied on patient feedback; another person (c1ffc) felt it was interesting but not essential.

Psychological factors (at follow-up/end of programme)

There was a mixture of responses given to several of these outcomes. The limited qualitative comments made by participants touched on the following. Firstly, one public respondent (ddcea) argued that these areas were important for achieving progress in terms of weight loss; yet another (0e844) stated that too often service providers wrongly correlate weight and psychological issues, adding, *"they need to ask more open ended questions and not cut off a response if they cannot put a tick in a box."*

Weight management staff were concerned that exploring all areas listed in this section would be onerous for them. Focusing on quality of life was proposed as a compromise. For example, 52291 (a policy respondent) wrote:

> *"Self-reported improvements to quality of life are crucial - has confidence to re-engage with friends, hobbies, work increased. Has any weight loss led to changes in their quality of life/have they set personal goals associated with weight loss."*

Programme specific outcomes

There was agreement about certain outcomes in this section (e.g. attendance, completion rates). However, areas of disagreement existed. Some people rated cost effectiveness highly, because this would be of interest to the public in terms of diligent spending of NHS funds. However, one public respondent questioned this, stating, *"Slightly confused. Weight loss clubs or groups have no tie-in to the NHS."* (92f98). Another public respondent queried the inclusion of medication (although still rating it 8):

> *"I don't think anti-obesity medication will work for most people - if the mindset does not change, they will continue to eat unhealthy food." (d5efb)*

Some participants questioned where data for these outcomes would come from as they are not collected routinely by weight management services. Reasons for dropout was mentioned as not necessarily easy to collect, but an outcome that could provide useful insights. In terms of reach, one policy maker respondent made the following observation:

> *"Given the vast number of people in the overweight category I am unclear reach tells us much other than that a weight management service can only ever reach a small proportion of those who might benefit - unless this is being used for wider population based approaches." (52291)*

Length of follow-up

Discrepancies were clear among participants in their views of time points for measuring outcomes. It was observed by a primary care worker that 3 and 12 months were standard in weight management services, but that 5 years might prove informative. However, it was noted by those running such services that longer follow-up periods would be difficult when patients were no longer attending:

> *"Provision of follow up data from a weight management programme provider is easily achievable if the participant is still engaged with the service, however experience shows participants are reluctant to provide follow up data to the service provider when no longer engaged and may be more likely to provide this information to the original health service referrer…" (f9a2d, weight management staff)*

One public respondent (ddcea) felt that taking measures earlier on might help with reinforcing positive patient behaviours, whereas 12 and 24 months may be less important in this respect. Another person added:

> *"On reflection the importance decreases as time passes, as too many other factors will influence the person's lifestyle, etc as time passes." (3d9b3, public)*

Supporting Information 7. Stage 1 (outcome selection), round 3 Delphi questionnaire as it appeared to participants.

CORE

Following two rounds of Delphi questionnaires, the outcomes below have been rated by the expert group as being most important with a mean (average) rating >7 *. As such, these outcomes will be considered **core** for measurement by weight management services.

* The average ratings for the highlighted outcomes marked with a * were not >7 but these outcomes are considered 'protected characteristics' and, in keeping with government guidelines, must be reported.

Time Point	Outcome	Importance	Disagreement	Mean	Median
At Baseline	Age	Important	No	7.2	8
At Baseline	*Gender	Important	No	6.8	8
At Baseline	*Ethnicity	Important	No	6.1	7
At Baseline	*Deprivation Category	Important	No	6.7	7
At Baseline	*Learning Disability	Important	No	6.2	7
At Baseline	*Physical Disability	Important	No	6.3	7
At Baseline	*Formally diagnosed with a mental health condition	New protected characteristic with no previous rating			
Baseline & Change In	Weight	Important	No	8.7	9
Baseline & Change In	Body Mass Index (BMI)	Important	No	8.3	9
Baseline & Change In	Diabetes Status	Important	No	7.5	8
Baseline & Change In	Quality of Life (QoL) Score	Important	No	7.2	8
At Follow-up	Referral to Specialist Services	Important	No	7.1	8
At Follow-up	Adverse Events/Unintended Consequences	Important	No	7.1	8
At Follow-up	Attendance	Important	No	8.3	9
At Follow-up	Completion	Important	No	8.5	9
At Follow-up	Reason for Dropout	Important	No	7.2	8
At Follow-up	Participant Satisfaction	Important	No	7.5	8
At Follow-up	Cost Effectiveness	Important	No	7.3	8
Follow-up Time Point	12 months	Important	No	8	9
Follow-up Time Point	24 months	Important	No	7.5	8

OPTIONAL

Following two rounds of Delphi questionnaires, the outcomes below have been rated by the expert group as being reasonably important with a mean (average) rating between 6.5 and 7.1. As such, these outcomes will be considered **optional** for measurement by weight management services.

Time Point	Outcome	Importance	Disagreement	Mean	Median
At Follow-up	Repeat Referrals	Important	No	7.1	7
Baseline & Change In	High Blood Pressure	Important	No	7	7
Baseline & Change In	Depression	Important	No	6.9	8
Baseline & Change In	High Future Risk of Diabetes (Impaired Fasting Glucose, Impaired Glucose Tolerance, Raised HbA1c Levels)	Important	No	6.8	7
Baseline & Change In	Overall Measure of Comorbidity	Important	No	6.8	7
Baseline & Change In	Binge Eating Disorder	Important	No	6.8	7
At Follow-up	Representativeness	Important	No	6.8	7
At Follow-up	Referral to Linked Services	Important	No	6.8	7
Follow-up Time Point	6 months	Important	No	6.8	7
At Baseline	Mobility Issues	Important	No	6.7	7
Baseline & Change In	Cardiovascular Risk	Important	No	6.6	7
Baseline & Change In	Self Confidence	Important	No	6.6	7
At Follow-up	Sources of Referral	Important	No	6.6	7
At Follow-up	Prescription of Anti-obesity Medication	Important	No	6.6	7
Follow-up Time Point	18 months	Important	No	6.6	7
At Baseline	High Cholesterol/ Lipids	Important	No	6.5	7

At Baseline	Importance of Weight Loss	Important	No	6.5	7
At Baseline	Disordered Eating	Important	No	6.5	7
Baseline & Change In	Blood Pressure	Important	No	6.5	7
Baseline & Change In	Self Esteem	Important	No	6.5	7
At Follow-up	Reach	Important	No	6.5	7
Follow-up Time Point	3 months	Important	No	6.5	7

1. * Do you accept these findings of the expert group?

- **Yes**
- **No**

2. * If you **do not accept** the findings of the expert group and feel very strongly that a particular outcome(s) should be excluded from either the core list or the optional list, or moved from the core list to the optional list or vice versa, please provide an explanation as to why in the box below. All suggestions will be given due consideration.
Please **do not suggest additional outcomes** for inclusion in the core or optional lists in this box. You will be given the opportunity to suggest additional outcomes for inclusion in the optional list in a subsequent box.

Maximum length: 5000 characters. Characters left: 5000

EXCLUSION

Following two rounds of Delphi questionnaires, the outcomes below have been rated by the expert group as being least important with a mean (average) rating <6.5. As such, these outcomes will be **excluded** which means we won't recommend they be measured by weight management services. (This doesn't mean that a weight management service cannot measure these outcomes should they wish to, it is just that measuring and reporting the other outcomes should be considered a higher priority.)

Time Point	Outcome	Importance	Disagreement	Mean	Median
Baseline & Change In	Confidence in Ability to Lose Weight	Important	No	6.4	7
Baseline & Change In	Sedentary Time	Important	No	6.4	7
At Follow-up	Importance of Weight Loss	Important	No	6.4	7
Baseline & Change In	Daily Fruit & Vegetable Intake	Important	No	6.3	7
Baseline & Change In	Fitness	Important	No	6.3	7
At Follow-up	Mobility Issues	Important	No	6.3	7
Baseline & Change In	Anxiety	Important	No	6.3	7
At Follow-up	Disordered Eating	Important	No	6.3	7
Baseline & Change In	Waist Circumference	Important	No	6.2	7
Baseline & Change In	Leisure Time Physical Activity	Important	No	6.2	7
Baseline & Change In	Body Image	Important	No	6.2	7
Baseline & Change In	Non Leisure Time Physical Activity	Important	No	6.1	7
At Follow-up	High Cholesterol/ Lipids	Unsure	No	6.1	6
At Baseline	Family History of Obesity	Important	No	6	7
At Baseline	Smoking Status	Important	No	6	7
Baseline & Change In	Suicidal Thoughts	Important	No	6	7
At Baseline	Advised To Lose Weight Prior To Routine Surgery	Unsure	No	6	6
At Baseline	Weight Loss History	Important	No	5.9	7
Baseline & Change In	Daily Alcohol Consumption	Important	No	5.9	7
At Baseline	Asthma	Important	No	5.9	7
Baseline & Change In	Other Addictive Behaviour	Important	No	5.9	7
Baseline & Change In	Fat Mass/Body Composition	Important	No	5.9	7
Baseline & Change In	Daily Calorie Consumption	Important	No	5.9	7
At Baseline	Osteoarthritis	Unsure	No	5.9	6
At Baseline	Non Alcoholic Fatty Liver Disease (NAFLD)	Unsure	No	5.9	6
Baseline & Change In	Overall Quality of Sleep	Unsure	No	5.9	6
Baseline & Change In	Obstructive Sleep Apnoea	Unsure	No	5.8	6
Baseline & Change In	Chronic Back Pain	Unsure	No	5.8	6
At Baseline	Other Health Conditions Requiring A Specialist Diet	Unsure	No	5.8	6

At Follow-up	Waist to Hip Ratio	Important	No	5.6	7
At Baseline	Chronic Kidney Disease	Unsure	No	5.6	6
At Baseline	Polycystic Ovary Syndrome (women only)	Unsure	No	5.6	6
At Baseline	Autism	Unsure	No	5.6	6
At Baseline	Personality Disorders	Unsure	No	5.6	6
At Follow-up	Daily Free Sugar Intake	Unsure	No	5.6	6
At Follow-up	Self Reported Reduction in Clothes Size	Unsure	No	5.5	6
Baseline & Change In	Neck Circumference	Unsure	No	4.9	5

3. * Do you accept the findings of the expert group and agree that the outcomes listed above should be **excluded**?

- **Yes**
- **No**

4. * If you **do not accept** the results of the expert group and feel very strongly that a particular outcome(s) should not be excluded and should, instead, be added to the list of optional outcomes, please provide an explanation as to why in the box below. All suggestions will be given due consideration.

Maximum length: 5000 characters. Characters left: 5000

Thank you, please click Save to finish.

Save

Prev 1 2 3 Next

Save to complete later

Next Page >

Supporting Information 8. Stage 1 (outcome selection), round 3 Delphi qualitative analysis.

Core and optional outcomes

Participants were shown the lists of proposed core and optional outcomes and asked whether or not they accepted the findings of the expert panel. Eight participants indicated that they were not in agreement and their comments are included below.

ddcea (public): *"Weight management can be a stressful experience and as such reinforcing success is of great importance. This can only be achieved if follow up times 3 months and 6 months are kept within the core outcomes. Not only will this benefit the patient, who is most important in the programme, but also lend assistance to the clinician in imparting praise. The same could also be said for hypertension, diabetes, self esteem, cardiovascular risk, all these help the patient by showing the benefits of weight management."*

18738 (academic): *"I wanted to put yes, and just a caveat, but it wouldn't let me add the caveat unless I ticked 'no'! On the whole, I agree with these. Seeing them here, I wonder how 1 year and 2 year follow up will work in practice, if treatment is <1 year. Will services have the responsibility to recall patients for follow up? Would end of treatment be more 'core'?"*

c1ffc (academic): *"I am surprised with the above lists. Unfortunately the "Core" list reflects a "weight centered" approach rather than focusing on health (which will include weight, I appreciate that QOL included in this core list). Focusing on weight related measurements as the main outcome measure will actually waste a lot of the efforts of Tier 3 WM teams without recognition. Also, tier 3 cost-effectiveness is really in doubt and not considering the impact of the intervention on the wider metabolic parameters and other health related issues seem illogical. As clinicians, we treat the patient holistically, and not just weight. The aims of of weight management are far reaching beyond just weight loss."*

52291 (policy maker): *"In taking a person centred and asset based approach to weight management, I feel it is important to understand the level of self efficacy of the person- the assets they feel they have personally and have wider access to in achieving their weight loss goal. As such, I would welcome consideration of self confidence/self esteem and importance of weight loss in the core outcomes at baseline and follow up."*

f9a2d (weight management staff): *"The feasibility of providing some of the core outcome measurements (mainly protected characteristics) will depend on who will be required to obtain this measure. The majority of these do not affect the ability to provide weight management support, or alter the support given and therefore an individual may not be wish to provide this information to a weight management provider. For some of the measures, it would not be appropriate for weight management providers to assess this measure but would instead need to be done by a health care professional. From previous experience within weight management referral services it has proved difficult to obtain these measures at point of referral."*

8b0d4 (weight management staff): *"I believe that including diabetes status, QOL score and cost effectiveness in the core list of outcomes is not required. Weight management programmes should be able to demonstrate that they impact on QOL and are cost effective in order to be commissioned - but collecting this data on an individual level outside formal research or indeed for a subset of participants, would add complexity and cost. Demonstrating diabetes status change would require either self reported status (and thus questionable validity) or blood measurements (adding significant cost and complexity to services) or linkages with GP systems (which again would add significant complexity and cost). Perhaps the positioning of the core requirements needs to be reviewed, as these state that; "these outcomes will be considered core for measurement by weight management services". This implies that it would be the responsibility of the services to measure and report on the core outcomes. If it were more of a systems requirement, that monitoring of locality diabetes rates were part of areas that had weight management services in place, this may be more achievable.*

Adding in diabetes status, QOL score and some form of cost effectiveness analytics will mean that valuable investments will need to go into these measurements, reducing the number of people services are able to support and thus the overall health impact on the nation. It is suggested that demonstrating impact on these core measures should be done on a programme basis through evidence and research and not on an individual participant level.

Being specific about what 'at follow up' will be important - as many of the items lists for follow up, should be reported at programme end, and some at follow up at the selected time points of 12 and 24 months. For example, attendance, completion, satisfaction and reason for drop out should be reported at programme end (whenever that is), yet weight and BMI change should be both programme end and at the specified follow up time points."

5035d (weight management staff): *"I agree with all but the point on 3 month follow up time being optional. This should be done as there is evidence to show that people who lose more weight in earlier stages are more likely to sustain a clinically beneficial weight loss at 12 months. This is a crucial time point to change the approach being used with patients which may be required to help them achieve clinically beneficial weight loss in the next 3 months."*

4ec71 (primary care staff): *"I feel that cardiovascular risk, mobility, depression and overall co-morbidity should be included as important measures of overall health and potential benefit of weight management services. The emphasis should be more on change in health outcomes rather than just weight change. Mobility and depression are important co-morbidities for this cohort of patients."*

Outcomes for exclusion

Participants were shown the list of proposed outcomes for exclusion and asked whether or not they accepted the findings of the expert panel. Eleven participants indicated that they were not in agreement and their comments are included below.

d5efb (public): *"Lipids should be checked - particularly if diabetic, as indicates poor blood sugar control. Some excess weight should be lost before surgery - to instill some personal responsibility for losing weight/improving health. Obese people tend to have fatty livers, and they need to be reduced prior to surgery, for safety. Non alcoholic fatty liver disease is an important measure. Chronic back pain is also important - it restricts mobility and exercise tolerance. Obstructive sleep apnoea can be vastly improved with weight loss - if not put into remission - so I feel it is an important outcome to measure. PCOS affects many women and can be the cause of large weight gains - important to include this, as it is a physical reason why weight loss may be be very slow. Chronic kidney disease is also important - a complication of Type 2 Diabetes, which is considered an important measure."*

ddcea (public): *"It all depends upon how this prorgamme is to be used or what the targets are. Is it aimed at patient success or researcher. If the former then several of these parameters rejected by the experts are in fact important to the patient - confidence, fruit & vegetable, waist to hip, reduction in cloth size in fact every thing that reinforces the benefits accruing from correct weight management. Obviously if this programme is solely for the researcher's work then most of these would be less important as they could be subjective rather than absolute."*

92f98 (public): *"Self reported reduction in clothes size should be considered as important as this is a reinforcing positive for the person losing weight."*

c1ffc (academic): *"BMI is included and WC is excluded. One of the main arguments about obesity not being a disease is the misleading nature of BMI. Waist circumference is more associated with health related outcomes that BMI; I find it strange not to include waist circumference. Not recording PCOS could open all sort of potential risky situations, not to mention that weight loss might cause ovulation and pregnancy while the patient is using Saxenda for example or have just had bariatric surgery. Knowing about PCOS and informing the women about the consequences and factoring the PCOS in the choice of management plans is important. Not reporting sleep apnoea, has the panel considered the potential consequences on driving and the DVLA related issues about this? Not reporting CKD at*

baseline is strange, this will affect the choice of treatment. How can smoking status be counted not important considering its impact on health, weight, and surgical risks. Sedentary behaviour and physical activity are useful as they reduce CVD and mortality (which is the ultimate aim of any treatment)."

49a6b (academic): *"Waist circumference is an important additional measure that must be included."*

52291 (policy maker): *"As per previous comment, confidence in ability to lose weight I feel is core."*

d7ff7 (commissioner): *"I think understanding sedentary time and physical activity levels and fruit and vegetables as a measure of healthy eating should be included in the optional list, to understand baseline and changes in lifestyle as a result of an intervention. For physical activity particularly as being more active helps clients to maintain the weight they have lost."*

3bd28 (weight management staff): *"I would add waist circumference and fruit and veg consumption to the optional list please."*

5035d (weight management staff): *"First three in this box and waist circumference I think should be recommended as optional measurements, not exclusions."*

4ec71 (primary care staff): *"Given the known importance of sedentary time and low physical activity as risk factors for morbidity and mortality, both sedentary time and physical activity measures should be included. Mobility issues should be included at follow up as per previous section this is an important co-morbidity for this cohort that has a significant impact on quality of life and functionality."*

62c76 (primary care staff): *"From a coaching perspective importance of weight loss to the person is vital as of it isn't important to them they won't be committed."*

Supporting Information 9. Stage 2 (instrument selection), round 1 Delphi questionnaire as it appeared to participants.

Question ID: 752

1. * I have read the Invite and Information Letter (v3.0 07/09/17) and I consent to participate in this Delphi process to select tools/instruments to measure core outcomes for lifestyle weight management. I know that my free text comments will be analysed and may be quoted in publications arising from this work. (You have to tick YES to participate in the rest of the questionnaire.)

- Yes
- No

Question ID: 753

2. * I consent to being named as a member of this development group in the acknowledgements of any publication arising from this work. (OPTIONAL)

- Yes
- No

Question ID: 754

CORE

Following the first three rounds of this Delphi process, the outcomes below have been identified as being core for measurement by weight management services. **Please rate the corresponding instrument(s)/measurement(s) in terms of their appropriateness for use on the 1-9 scale.**

Question ID: 755

3. Age

How old participants are/the age (in years) of participants

Question ID: 756

Baseline Instrument/Measurement/Presentation

Question ID: 757

3.1. * mean age of participants in years

Inappropriate | | | | | | | | Very Appropriate

1	2	3	4	5	6	7	8	9

Question ID: 758

3.2. * % of participants in age bands (16-24 years, 25-34 years, 35-44 years, 45-54 years, 55-64 years, 65-74 years, 75+ years)

Inappropriate | | | | | | | | Very Appropriate

1	2	3	4	5	6	7	8	9

Question ID: 759

4. Weight

The measurement of how heavy a participant is in kilograms (kg) or stones and pounds

Question ID: 760

Baseline Instrument/Measurement/Presentation

Question ID: 761

4.1. * mean weight of participants in kg

Inappropriate | | | | | | | | Very Appropriate

1	2	3	4	5	6	7	8	9

Question ID: 762

Follow Up Instrument/Measurement/Presentation

Question ID: 763

4.2. * mean change in participants' weight in kg

Inappropriate | | | | | | | | Very Appropriate

1	2	3	4	5	6	7	8	9

Question ID: 764

4.3. * mean % weight change of participants

Inappropriate								Very Appropriate
1	2	3	4	5	6	7	8	9

Question ID: 765

4.4. * % of participants achieving ≥ 3% weight loss

Inappropriate								Very Appropriate
1	2	3	4	5	6	7	8	9

Question ID: 766

4.5. * % of participants achieving ≥ 5% weight loss

Inappropriate								Very Appropriate
1	2	3	4	5	6	7	8	9

Question ID: 767

4.6. * % of participants achieving ≥ 10% weight loss

Inappropriate								Very Appropriate
1	2	3	4	5	6	7	8	9

Question ID: 768

4.7. * % of participants achieving ≥ 3kg weight loss

Inappropriate								Very Appropriate
1	2	3	4	5	6	7	8	9

Question ID: 769

4.8. * % of participants achieving ≥ 5kg weight loss

Inappropriate								Very Appropriate
1	2	3	4	5	6	7	8	9

Question ID: 770

4.9. * % of participants achieving ≥ 10kg weight loss

Inappropriate								Very Appropriate
1	2	3	4	5	6	7	8	9

Question ID: 771

5. Body Mass Index (BMI)

An approximate measure of whether a participant is overweight or underweight, calculated by dividing their weight in kilograms by the square of their height in metres

Question ID: 772

Baseline Instrument/Measurement/Presentation

Question ID: 773

5.1. * mean BMI of participants

Inappropriate								Very Appropriate
1	2	3	4	5	6	7	8	9

Question ID: 774

5.2. * % of participants in BMI categories <25, 25-29.9, 30-34.9, 35-39.9, 40-49.9, 50-59.9, ≥ 60

Inappropriate								Very Appropriate
1	2	3	4	5	6	7	8	9

Question ID: 775

Follow Up Instrument/Measurement/Presentation

Question ID: 776

5.3. * mean change in participants' BMI

Inappropriate								Very Appropriate
1	2	3	4	5	6	7	8	9

Question ID: 777

5.4. * % of participants achieving BMI <25

Inappropriate								Very Appropriate
1	2	3	4	5	6	7	8	9

Question ID: 778

5.5. * % of participants achieving BMI <30

Inappropriate								Very Appropriate
1	2	3	4	5	6	7	8	9

Question ID: 779

5.6. You may use this space to elaborate on your views in relation to any the above instruments/measurements. Please note to which of the above instruments/measurements your comment(s) relates.

Maximum length: 5000 characters. Characters left: 5000

Question ID: 780

5.7. Please add any additional instruments/measurement(s) in the box below.

Maximum length: 5000 characters. Characters left: 5000

Question ID: 781

6. Diabetes Status

Whether a participant has diabetes, a condition which occurs when the body doesn't produce enough insulin to function properly, or the body's cells don't react to insulin. This means glucose stays in the blood and isn't used as fuel for energy. Type 2 diabetes is often associated with obesity and an increased risk of developing cardiovascular disease.

Question ID: 782

Baseline Instrument/Measurement/Presentation

Question ID: 783

6.1. * % of participants with type 1 diabetes mellitus (based on self -report, case record or blood test)

Inappropriate								Very Appropriate
1	2	3	4	5	6	7	8	9

Question ID: 784

6.2. * % of participants with type 2 diabetes mellitus (based on self -report, case record or blood test)

Inappropriate								Very Appropriate
1	2	3	4	5	6	7	8	9

Question ID: 785

6.3. * mean HbA1c levels of those participants with type 2 diabetes mellitus (T2DM)

Inappropriate								Very Appropriate
1	2	3	4	5	6	7	8	9

Question ID: 786

6.4. * % of those participants with T2DM who are on insulin

Inappropriate Very Appropriate

1	2	3	4	5	6	7	8	9

Question ID: 787

6.5. * mean number of diabetes medications per participant with T2DM

Inappropriate Very Appropriate

1	2	3	4	5	6	7	8	9

Question ID: 788

Follow Up Instrument/Measurement/Presentation

Question ID: 789

6.6. * mean change in HbA1c levels of those participants with T2DM

Inappropriate Very Appropriate

1	2	3	4	5	6	7	8	9

Question ID: 790

6.7. * mean change in % of participants with T2DM who are on insulin

Inappropriate Very Appropriate

1	2	3	4	5	6	7	8	9

Question ID: 791

6.8. * mean change in number of diabetes medications per participant with T2DM

Inappropriate Very Appropriate

1	2	3	4	5	6	7	8	9

Question ID: 792

7. Quality of Life (QoL) Score

A measure of the general well-being of participants. Various questionnaires can be used to obtain a quality of life score. Information on each questionnaire and a comparison of some of the different questionnaires can be found in (document 1)

Question ID: 793

Baseline Instrument/Measurement/Presentation

Question ID: 794

7.1. * mean EQ-5D-5L scores of participants

Inappropriate Very Appropriate

1	2	3	4	5	6	7	8	9

Question ID: 795

7.2. * mean SF12 score of participants

Inappropriate Very Appropriate

1	2	3	4	5	6	7	8	9

Question ID: 796

7.3. * mean SF36 scores of participants

Inappropriate Very Appropriate

1	2	3	4	5	6	7	8	9

Question ID: 797

7.4. * mean IWQOL-Lite score of participants

Inappropriate Very Appropriate

1	2	3	4	5	6	7	8	9

Question ID: 798

7.5. * mean OWLQOL scores of participants

Inappropriate								Very Appropriate
1	2	3	4	5	6	7	8	9

Question ID: 799

Follow Up Instrument/Measurement/Presentation

Question ID: 800

7.6. * mean EQ-5D-5L scores of participants

Inappropriate								Very Appropriate
1	2	3	4	5	6	7	8	9

Question ID: 801

7.7. * mean SF12 score of participants

Inappropriate								Very Appropriate
1	2	3	4	5	6	7	8	9

Question ID: 802

7.8. * mean SF36 scores of participants

Inappropriate								Very Appropriate
1	2	3	4	5	6	7	8	9

Question ID: 803

7.9. * mean IWQOL-Lite score of participants

Inappropriate								Very Appropriate
1	2	3	4	5	6	7	8	9

Question ID: 804

7.10. * mean OWLQOL scores of participants

Inappropriate								Very Appropriate
1	2	3	4	5	6	7	8	9

Question ID: 805

8. Learning Disability QoL Score

A measure of the general well-being of participants with a learning disability. Various questionnaires can be used to obtain a quality of life score. Information and a comparison of some of the different questionnaires can be found in (document 2)

Question ID: 806

Baseline Instrument/Measurement/Presentation

Question ID: 807

8.1. * mean PWI-ID score(s) of participants

Inappropriate								Very Appropriate
1	2	3	4	5	6	7	8	9

Question ID: 808

8.2. * mean score obtained using another suitable instrument (Please make suggestions in the box below.)

Inappropriate								Very Appropriate
1	2	3	4	5	6	7	8	9

Question ID: 809

8.3. You may use this space to elaborate on your views in relation to any the above instruments/measurements. Please note to which of the above instruments/measurements your comment(s) relates.

Maximum length: 5000 characters. Characters left: 5000

Question ID: 810

8.4. Please add any additional instruments/measurement(s) in the box below.

Maximum length: 5000 characters. Characters left: 5000

Question ID: 811

9. Adverse Events/Unintended Consequences

Whether participants suffered any unfortunate side effects as a result of attending the weight loss service.

Question ID: 812

Follow Up Instrument/Measurement/Presentation

Question ID: 813

9.1. * number of participants experiencing a worsening of a pre-existing medical condition, such as an undiagnosed eating disorder, other pre-existing medical conditions (Please make suggestions in the box below.)

Inappropriate — Very Appropriate

1	2	3	4	5	6	7	8	9

Question ID: 814

9.2. * number of participants suffering severe hypoglycaemia

Inappropriate — Very Appropriate

1	2	3	4	5	6	7	8	9

Question ID: 815

9.3. * number of participants sustaining an injury during a physical activity session run by the weight management service

Inappropriate — Very Appropriate

1	2	3	4	5	6	7	8	9

Question ID: 816

9.4. * number of participants experiencing other side effects (Please make suggestions in the box below.)

Inappropriate — Very Appropriate

1	2	3	4	5	6	7	8	9

Question ID: 817

10. Repeat Referrals

Whether a participant has been referred to the weight management service on more than one occasion.

Question ID: 818

Follow Up Instrument/Measurement/Presentation

Question ID: 819

10.1. * % of participants previously referred to the service, not necessarily having attended any sessions

Inappropriate — Very Appropriate

1	2	3	4	5	6	7	8	9

Question ID: 820

10.2. * % of participants answering yes, having previously attended at least 1 weight management session

Inappropriate — Very Appropriate

1	2	3	4	5	6	7	8	9

Question ID: 821

10.3. You may use this space to elaborate on your views in relation to any the above instruments/measurements. Please note to which of the above instruments/measurements your comment(s) relates.

Maximum length: 5000 characters. Characters left: 5000

Question ID: 822

10.4. Please add any additional instruments/measurement(s) in the box below.

Maximum length: 5000 characters. Characters left: 5000

Question ID: 823

11. Attendance

How many people attended the weight management service

Question ID: 824

Follow Up Instrument/Measurement/Presentation

Question ID: 825

11.1. * mean % of core/mandatory sessions attended by participants

Inappropriate								Very Appropriate
1	2	3	4	5	6	7	8	9

Question ID: 826

11.2. * % of participants attending 100% of core/mandatory sessions

Inappropriate								Very Appropriate
1	2	3	4	5	6	7	8	9

Question ID: 1016

11.3. * % of participants attending ≥80% of core/mandatory sessions

Inappropriate								Very Appropriate
1	2	3	4	5	6	7	8	9

Question ID: 1017

11.4. * % of participants attending ≥70% core/mandatory sessions

Inappropriate								Very Appropriate
1	2	3	4	5	6	7	8	9

Question ID: 1018

11.5. * % of participants attending ≥50% core/mandatory sessions

Inappropriate								Very Appropriate
1	2	3	4	5	6	7	8	9

Question ID: 827

12. Completion

How many people finished the weight management programme

Question ID: 828

Follow Up Instrument/Measurement/Presentation

Question ID: 829

12.1. * % of participants who attended 100% of possible/core/mandatory sessions

Inappropriate								Very Appropriate
1	2	3	4	5	6	7	8	9

Question ID: 830

12.2. * % of participants who attended 80% of possible/core/mandatory sessions

Inappropriate								Very Appropriate
1	2	3	4	5	6	7	8	9

Question ID: 831

12.3. * % of participants who attended 70% of possible/core/mandatory sessions

Inappropriate								Very Appropriate
1	2	3	4	5	6	7	8	9

Question ID: 832

12.4. * % of participants who attended 50% of possible/core/mandatory sessions

Inappropriate								Very Appropriate
1	2	3	4	5	6	7	8	9

Question ID: 833

12.5. You may use this space to elaborate on your views in relation to any the above instruments/measurements. Please note to which of the above instruments/measurements your comment(s) relates.

Maximum length: 5000 characters. Characters left: 5000

Question ID: 834

12.6. Please add any additional instruments/measurement(s) in the box below.

Maximum length: 5000 characters. Characters left: 5000

Question ID: 835

13. Reason for Dropout

Why those participants who did not complete the programme failed to do so.

Question ID: 836

Follow Up Instrument/Measurement/Presentation

Question ID: 837

13.1. * % of participants who dropped out due to dissatisfaction with the intervention (unrelated to weight loss)

Inappropriate								Very Appropriate
1	2	3	4	5	6	7	8	9

Question ID: 838

13.2. * % of participants who dropped out due to poor weight loss

Inappropriate								Very Appropriate
1	2	3	4	5	6	7	8	9

Question ID: 839

13.3. * % of participants who dropped out due to illness/ hospitalisation

Inappropriate								Very Appropriate
1	2	3	4	5	6	7	8	9

Question ID: 840

13.4. * % of participants who dropped out due to pregnancy

Inappropriate								Very Appropriate
1	2	3	4	5	6	7	8	9

Question ID: 841

13.5. * % of participants who dropped out due to change in personal circumstances/social reason

Inappropriate								Very Appropriate
1	2	3	4	5	6	7	8	9

Question ID: 842

13.6. * % of participants who dropped out due to moving from the geographical area

Inappropriate								Very Appropriate
1	2	3	4	5	6	7	8	9

Question ID: 843

13.7. * % of participants who dropped out due to any other reason

Inappropriate								Very Appropriate
1	2	3	4	5	6	7	8	9

Question ID: 844

14. Participant Satisfaction

How happy/satisfied participants were with the weight loss service.
Various questionnaires can be used to obtain a participant satisfaction score. Information on each questionnaire can be found in (document 3).

Question ID: 845

Follow Up Instrument/Measurement/Presentation

Question ID: 846

14.1. * mean Outcomes and Experiences Questionnaire (OEQ) score adapted to suit weight management services

Inappropriate								Very Appropriate
1	2	3	4	5	6	7	8	9

Question ID: 847

14.2. * mean NHS Friends and Family Test (FFT) score

Inappropriate								Very Appropriate
1	2	3	4	5	6	7	8	9

Question ID: 848

15. Cost Effectiveness

The value for money of the weight management service in terms of long term economic benefits to the NHS.

Question ID: 849

Follow Up Instrument/Measurement/Presentation

Question ID: 850

15.1. * The Public Health England Weight Management Economic Assessment Tool:
http://webarchive.nationalarchives.gov.uk/20170110165804/http://www.noo.org.uk/visualisation/economic_assessment_tool

Inappropriate								Very Appropriate
1	2	3	4	5	6	7	8	9

Question ID: 851

15.2. * cost / kg (based on mean weight loss)

Inappropriate								Very Appropriate
1	2	3	4	5	6	7	8	9

Question ID: 852

15.3. * cost per ‘success’ with success being 5% weight loss

Inappropriate								Very Appropriate
1	2	3	4	5	6	7	8	9

Question ID: 853

15.4. * cost per ‘success’ with success being 5kg weight loss

Inappropriate								Very Appropriate
1	2	3	4	5	6	7	8	9

Question ID: 854

15.5. * cost per 'success' with success being 3% weight loss

Inappropriate								Very Appropriate
1	2	3	4	5	6	7	8	9

Question ID: 855

15.6. * cost per kg based on any participant with a change in weight data

Inappropriate								Very Appropriate
1	2	3	4	5	6	7	8	9

Question ID: 856

15.7. You may use this space to elaborate on your views in relation to any the above instruments/measurements. Please note to which of the above instruments/measurements your comment(s) relates.

Maximum length: 5000 characters. Characters left: 5000

Question ID: 857

15.8. Please add any additional instruments/measurement(s) in the box below.

Maximum length: 5000 characters. Characters left: 5000

Question ID: 858

16. Presentation of Results

Which participants' outcomes to include in reporting

Question ID: 859

Follow Up Instrument/Measurement/Presentation

Question ID: 860

16.1. * report outcomes for all participants attending ≥1 active weight loss sessions (does not include introductory sessions/information sessions about the service)

Inappropriate								Very Appropriate
1	2	3	4	5	6	7	8	9

Question ID: 861

16.2. * report outcomes for all participants attending >1 active weight loss session(s) and therefore having weight change data (does not include introductory sessions/information sessions about the service)

Inappropriate								Very Appropriate
1	2	3	4	5	6	7	8	9

Question ID: 862

16.3. * report outcomes for all participants completing the programme

Inappropriate								Very Appropriate
1	2	3	4	5	6	7	8	9

Question ID: 863

16.4. You may use this space to elaborate on your views in relation to any the above instruments/measurements. Please note to which of the above instruments/measurements your comment(s) relates.

Maximum length: 5000 characters. Characters left: 5000

Question ID: 864

16.5. Please add any additional instruments/measurement(s) in the box below.

Maximum length: 5000 characters. Characters left: 5000

Question ID: 865

OPTIONAL

Following the first three rounds of this Delphi process, the outcomes below have been identified as being optional for measurement by weight management services. Please rate the corresponding instrument(s)/measurement(s) in terms of their appropriateness for use on the 1-9 scale.

Question ID: 866

17. High Blood Pressure

Whether a participant has high blood pressure. High blood pressure increases the risk of developing cardiovascular disease.

Question ID: 867

Baseline Instrument/Measurement/Presentation

Question ID: 868

17.1. * % of participants with high blood pressure based on patient report/medication/case notes

Inappropriate								Very Appropriate
1	2	3	4	5	6	7	8	9

Question ID: 869

17.2. * % of participants with high blood pressure based on blood pressure readings

Inappropriate								Very Appropriate
1	2	3	4	5	6	7	8	9

Question ID: 870

17.3. * mean number of blood pressure medications per participant with high blood pressure

Inappropriate								Very Appropriate
1	2	3	4	5	6	7	8	9

Question ID: 871

Follow Up Instrument/Measurement/Presentation

Question ID: 872

17.4. * change in mean blood pressure (systolic/diastolic, mmHg)

Inappropriate								Very Appropriate
1	2	3	4	5	6	7	8	9

Question ID: 873

17.5. * change in mean number of blood pressure medications per participant with high blood pressure

Inappropriate								Very Appropriate
1	2	3	4	5	6	7	8	9

Question ID: 874

18. Blood Pressure

The pressure of blood in the arteries, the vessels that carry blood from the heart to the rest of the body. A certain amount of pressure is required to get the blood around the body but consistently high blood pressure increases the risk of cardiovascular disease.

Question ID: 875

Baseline Instrument/Measurement/Presentation

Question ID: 876

18.1. * mean systolic and diastolic blood pressure of participants

Inappropriate								Very Appropriate
1	2	3	4	5	6	7	8	9

Question ID: 877

18.2. * % of participants with blood pressure >140/80 mmHg

Inappropriate Very Appropriate

1	2	3	4	5	6	7	8	9

Question ID: 878

18.3. * % of participants on blood pressure medication based on self-report/case records

Inappropriate Very Appropriate

1	2	3	4	5	6	7	8	9

Question ID: 879

Follow Up Instrument/Measurement/Presentation

Question ID: 880

18.4. * change in mean systolic and diastolic blood pressure of participants

Inappropriate Very Appropriate

1	2	3	4	5	6	7	8	9

Question ID: 881

18.5. * change in % of participants with blood pressure >140/80 mmHg

Inappropriate Very Appropriate

1	2	3	4	5	6	7	8	9

Question ID: 882

18.6. * change in % of participants on blood pressure medication based on self-report/case records

Inappropriate Very Appropriate

1	2	3	4	5	6	7	8	9

Question ID: 883

19. Cardiovascular Risk

A measure of how likely participants are to develop cardiovascular disease, including heart disease and stroke

Question ID: 884

Baseline Instrument/Measurement/Presentation

Question ID: 885

19.1. * % of participants with previous cardiovascular disease (CVD), including myocardial infarction, stroke, transient ischaemic attack (TIA), angina and peripheral vascular disease

Inappropriate Very Appropriate

1	2	3	4	5	6	7	8	9

Question ID: 886

19.2. * % of participants with high CVD risk (previous CVD or a high cardiovascular risk score - N.B. information on blood pressure and lipids would be required to calculate the risk score)

Inappropriate Very Appropriate

1	2	3	4	5	6	7	8	9

Question ID: 887

19.3. * % of participants with a high cardiovascular risk score (primary prevention/not those with previous cardiovascular disease)

Inappropriate Very Appropriate

1	2	3	4	5	6	7	8	9

Question ID: 888

19.4. * mean CVD risk score of participants

Inappropriate Very Appropriate

1	2	3	4	5	6	7	8	9

Question ID: 889

19.5. * % of participants on cardiovascular medication(s)

Inappropriate Very Appropriate

1	2	3	4	5	6	7	8	9

Question ID: 890

19.6. * mean number of cardiovascular medications per participant on cardiovascular medication(s)

Inappropriate Very Appropriate

1	2	3	4	5	6	7	8	9

Question ID: 891

Follow Up Instrument/Measurement/Presentation

Question ID: 892

19.7. * % of participants with a high cardiovascular risk score

Inappropriate Very Appropriate

1	2	3	4	5	6	7	8	9

Question ID: 893

19.8. * change in mean cardiovascular risk score of participants

Inappropriate Very Appropriate

1	2	3	4	5	6	7	8	9

Question ID: 894

19.9. * change in % of participants on cardiovascular medication(s)

Inappropriate Very Appropriate

1	2	3	4	5	6	7	8	9

Question ID: 895

19.10. * change in mean number of cardiovascular medications per participant on cardiovascular medication(s)

Inappropriate Very Appropriate

1	2	3	4	5	6	7	8	9

Question ID: 896

20. High Cholesterol/ Lipids

A measure of whether a participant has an abnormal amount of fat and/or cholesterol, known as lipids, in their blood (also called dyslipidaemia). Being overweight can increase the likelihood of developing dyslipidaemia. Dyslipidaemia is associated with increased risk of developing cardiovascular disease.

Question ID: 897

Baseline Instrument/Measurement/Presentation

Question ID: 898

20.1. * % of participants with high cholesterol/lipids based on self-report /case records

Inappropriate Very Appropriate

1	2	3	4	5	6	7	8	9

Question ID: 899

20.2. * % of participants on statin/ lipid lowering medication based on self-report/case records

Inappropriate Very Appropriate

1	2	3	4	5	6	7	8	9

Question ID: 900

20.3. * mean total cholesterol/ high density lipoprotein/ triglycerides of participants as obtained via blood test

Inappropriate / Very Appropriate

1	2	3	4	5	6	7	8	9

Question ID: 901

Follow Up Instrument/Measurement/Presentation

Question ID: 902

20.4. * % of participants with high cholesterol/lipids based on self-report /case records

Inappropriate / Very Appropriate

1	2	3	4	5	6	7	8	9

Question ID: 903

20.5. * % of participants on statin/ lipid lowering medication – based on self-report/case records

Inappropriate / Very Appropriate

1	2	3	4	5	6	7	8	9

Question ID: 904

20.6. * mean total cholesterol/ high density lipoprotein/ triglycerides of participants as obtained via blood test

Inappropriate / Very Appropriate

1	2	3	4	5	6	7	8	9

Question ID: 905

21. High Future Risk of Diabetes (Impaired Fasting Glucose, Impaired Glucose Tolerance, Raised HbA1c Levels, Previous Gestational Diabetes)

Whether measures of the amount of glucose in a participant's blood suggests he/she is likely to develop type 2 diabetes in the future.

Question ID: 906

Baseline Instrument/Measurement/Presentation

Question ID: 907

21.1. * % of participants with a medical record of high diabetes risk (HDR)

Inappropriate / Very Appropriate

1	2	3	4	5	6	7	8	9

Question ID: 908

21.2. * % of participants with HDR as determined by an oral glucose tolerance test (OGTT)

Inappropriate / Very Appropriate

1	2	3	4	5	6	7	8	9

Question ID: 909

21.3. * % of participants with HDR as determined by measuring HbA1c levels

Inappropriate / Very Appropriate

1	2	3	4	5	6	7	8	9

Question ID: 910

Follow Up Instrument/Measurement/Presentation

Question ID: 911

21.4. * % of participants with a medical record of HDR (HDR is followed-up annually in primary care)

Inappropriate / Very Appropriate

1	2	3	4	5	6	7	8	9

Question ID: 912

21.5. * % of all participants with HDR as determined by OGTT

Inappropriate Very Appropriate

1	2	3	4	5	6	7	8	9

Question ID: 913

21.6. * % of those participants identified as having HDR at baseline who still have HDR as determined by OGTT

Inappropriate Very Appropriate

1	2	3	4	5	6	7	8	9

Question ID: 914

21.7. * % of all participants with HDR as determined by measuring HbA1c levels

Inappropriate Very Appropriate

1	2	3	4	5	6	7	8	9

Question ID: 915

21.8. * % of those participants identified as having HDR at baseline who still have HDR as determined by HbA1c levels

Inappropriate Very Appropriate

1	2	3	4	5	6	7	8	9

Question ID: 916

21.9. You may use this space to elaborate on your views in relation to any the above instruments/measurements. Please note to which of the above instruments/measurements your comment(s) relates.

Maximum length: 5000 characters. Characters left: 5000

Question ID: 917

21.10. Please add any additional instruments/measurement(s) in the box below.

Maximum length: 5000 characters. Characters left: 5000

Question ID: 918

22. Overall Measure of Comorbidity

Measure of the presence of additional diseases or disorders co-occurring with obesity/being overweight
Various indexes or scoring systems can be used to obtain a measure of comorbidity. Information on each can be found in (document 4).

Question ID: 919

Baseline Instrument/Measurement/Presentation

Question ID: 920

22.1. * mean CCI score

Inappropriate Very Appropriate

1	2	3	4	5	6	7	8	9

Question ID: 921

22.2. * mean EOSS score

Inappropriate Very Appropriate

1	2	3	4	5	6	7	8	9

Question ID: 922

22.3. * mean Chronic Disease Score

Inappropriate Very Appropriate

1	2	3	4	5	6	7	8	9

Question ID: 923

22.4. * mean number of dispensed medications per participant

Inappropriate								Very Appropriate
1	2	3	4	5	6	7	8	9

Question ID: 924

Follow Up Instrument/Measurement/Presentation

Question ID: 925

22.5. * mean CCI score

Inappropriate								Very Appropriate
1	2	3	4	5	6	7	8	9

Question ID: 926

22.6. * mean EOSS score

Inappropriate								Very Appropriate
1	2	3	4	5	6	7	8	9

Question ID: 927

22.7. * mean Chronic Disease Score

Inappropriate								Very Appropriate
1	2	3	4	5	6	7	8	9

Question ID: 928

22.8. * mean number of dispensed medications per participant

Inappropriate								Very Appropriate
1	2	3	4	5	6	7	8	9

Question ID: 929

23. Depression

Whether a participant suffers from a mental illness characterised by a profound and persistent feeling of sadness or despair and/or a loss of interest in things that once were pleasurable.
Various questionnaires can be used to obtain a depression score. Information on each questionnaire can be in (document 5).

Question ID: 930

Baseline Instrument/Measurement/Presentation

Question ID: 931

23.1. * % of participants with depression based on patient report/medication/case notes

Inappropriate								Very Appropriate
1	2	3	4	5	6	7	8	9

Question ID: 932

23.2. * % of participants on medication for depression

Inappropriate								Very Appropriate
1	2	3	4	5	6	7	8	9

Question ID: 933

23.3. * mean HADS questionnaire score of participants

Inappropriate								Very Appropriate
1	2	3	4	5	6	7	8	9

Question ID: 934

23.4. * mean PHQ9 questionnaire score of participants

Inappropriate								Very Appropriate
1	2	3	4	5	6	7	8	9

Question ID: 935

23.5. * mean Beck Depression Inventory score of participants

Inappropriate								Very Appropriate
1	2	3	4	5	6	7	8	9

Question ID: 936

Follow Up Instrument/Measurement/Presentation

Question ID: 937

23.6. * % of all participants on medication for depression

Inappropriate								Very Appropriate
1	2	3	4	5	6	7	8	9

Question ID: 938

23.7. * % of those patients identified as having depression at baseline on medication for depression

Inappropriate								Very Appropriate
1	2	3	4	5	6	7	8	9

Question ID: 939

23.8. * mean HADS questionnaire score of participants

Inappropriate								Very Appropriate
1	2	3	4	5	6	7	8	9

Question ID: 940

23.9. * mean PHQ9 questionnaire score of participants

Inappropriate								Very Appropriate
1	2	3	4	5	6	7	8	9

Question ID: 941

23.10. * mean Beck Depression Inventory score of participants

Inappropriate								Very Appropriate
1	2	3	4	5	6	7	8	9

Question ID: 942

23.11. You may use this space to elaborate on your views in relation to any the above instruments/measurements. Please note to which of the above instruments/measurements your comment(s) relates.

Maximum length: 5000 characters. Characters left: 5000

Question ID: 943

23.12. Please add any additional instruments/measurement(s) in the box below.

Maximum length: 5000 characters. Characters left: 5000

Question ID: 944

24. Self-confidence & Self-esteem

How participants feel about their own abilities and worth
Various questionnaires can be used to obtain a self-confidence/self-esteem score and a measure of general well-being. Information on each questionnaire can be found in (document 6).

Question ID: 945

Baseline Instrument/Measurement/Presentation

Question ID: 946

24.1. * mean Tennesse Self-concept Scale score

Inappropriate								Very Appropriate
1	2	3	4	5	6	7	8	9

Question ID: 947

24.2. * mean Rosenberg Self-esteem Scale score

Inappropriate								Very Appropriate
1	2	3	4	5	6	7	8	9

Question ID: 948

24.3. * mean General Well-being Schedule score

Inappropriate								Very Appropriate
1	2	3	4	5	6	7	8	9

Question ID: 949

24.4. * mean ICECAP-A score

Inappropriate								Very Appropriate
1	2	3	4	5	6	7	8	9

Question ID: 950

24.5. * mean WEMWBS score

Inappropriate								Very Appropriate
1	2	3	4	5	6	7	8	9

Question ID: 951

Follow Up Instrument/Measurement/Presentation

Question ID: 952

24.6. * mean Tennesse Self-concept Scale score

Inappropriate								Very Appropriate
1	2	3	4	5	6	7	8	9

Question ID: 953

24.7. * mean Rosenberg Self-esteem Scale score

Inappropriate								Very Appropriate
1	2	3	4	5	6	7	8	9

Question ID: 954

24.8. * mean General Well-being Schedule score

Inappropriate								Very Appropriate
1	2	3	4	5	6	7	8	9

Question ID: 955

24.9. * mean ICECAP-A score

Inappropriate								Very Appropriate
1	2	3	4	5	6	7	8	9

Question ID: 956

24.10. * mean WEMWBS score

Inappropriate								Very Appropriate
1	2	3	4	5	6	7	8	9

Question ID: 957

25. Importance of Weight Loss

How important participants feel it is for them to lose weight
Various scales can be used to obtain an importance of weight loss/dieting readiness score. Information on each scale can be found in (document 7).

Question ID: 958

Baseline Instrument/Measurement/Presentation

Question ID: 959

25.1. * mean Dieting Readiness Scale score(s)

Inappropriate Very Appropriate

1	2	3	4	5	6	7	8	9

Question ID: 960

25.2. * mean DIET score(s)

Inappropriate Very Appropriate

1	2	3	4	5	6	7	8	9

Question ID: 961

25.3. * mean Self-Efficacy for Eating Behaviours Scale score(s)

Inappropriate Very Appropriate

1	2	3	4	5	6	7	8	9

Question ID: 962

Follow Up Instrument/Measurement/Presentation

Question ID: 963

25.4. * mean Dieting Readiness Scale score(s)

Inappropriate Very Appropriate

1	2	3	4	5	6	7	8	9

Question ID: 964

25.5. * mean DIET score(s)

Inappropriate Very Appropriate

1	2	3	4	5	6	7	8	9

Question ID: 965

25.6. * mean Self-Efficacy for Eating Behaviours Scale score(s)

Inappropriate Very Appropriate

1	2	3	4	5	6	7	8	9

Question ID: 966

26. Disordered Eating

Whether participants have disturbed and unhealthy eating patterns that can include restrictive dieting, compulsive eating or skipping meals. Disordered eating can include behaviours which reflect many but not all of the symptoms of feeding and eating disorders such as anorexia nervosa, bulimia nervosa and binge eating disorder.
Various questionnaires can be used to obtain a disordered eating score. Information on each questionnaire can be found in (document 8).

Question ID: 967

Baseline Instrument/Measurement/Presentation

Question ID: 968

26.1. * % of participants with disordered eating (defined as per service)

Inappropriate Very Appropriate

1	2	3	4	5	6	7	8	9

Question ID: 969

26.2. * mean TFEQ score

Inappropriate Very Appropriate

1	2	3	4	5	6	7	8	9

Question ID: 970

26.3. * mean EDEQ score

Inappropriate Very Appropriate

1	2	3	4	5	6	7	8	9

Question ID: 971

26.4. * mean BES score

Inappropriate Very Appropriate

1	2	3	4	5	6	7	8	9

Question ID: 972

26.5. * mean QEWP score

Inappropriate Very Appropriate

1	2	3	4	5	6	7	8	9

Question ID: 973

Follow Up Instrument/Measurement/Presentation

Question ID: 974

26.6. * % of participants with disordered eating (defined as per service)

Inappropriate Very Appropriate

1	2	3	4	5	6	7	8	9

Question ID: 975

26.7. * mean TFEQ score

Inappropriate Very Appropriate

1	2	3	4	5	6	7	8	9

Question ID: 976

26.8. * mean EDEQ score

Inappropriate Very Appropriate

1	2	3	4	5	6	7	8	9

Question ID: 977

26.9. * mean BES score

Inappropriate Very Appropriate

1	2	3	4	5	6	7	8	9

Question ID: 978

26.10. * mean QEWP score

Inappropriate Very Appropriate

1	2	3	4	5	6	7	8	9

Question ID: 979

26.11. You may use this space to elaborate on your views in relation to any the above instruments/measurements. Please note to which of the above instruments/measurements your comment(s) relates.

Maximum length: 5000 characters. Characters left: 5000

Question ID: 980

26.12. Please add any additional instruments/measurement(s) in the box below.

Maximum length: 5000 characters. Characters left: 5000

Question ID: 981

27. Reach (% eligible population who are referred to/take up weight management service)

The percentage of the eligible population (people who are overweight or obese within that particular geographical area) referred to the weight management service.

Question ID: 982

Follow Up Instrument/Measurement/Presentation

Question ID: 1015

For a specific population subgroup of concern, what % of that population has been referred to/ attended the weight management service. Local data (e.g. Quality and Outcomes Framework) can be used to obtain prevalence rates. Population subgroups of interest:

Question ID: 983

27.1. * age <30

Inappropriate								Very Appropriate
1	2	3	4	5	6	7	8	9

Question ID: 1012

27.2. * male

Inappropriate								Very Appropriate
1	2	3	4	5	6	7	8	9

Question ID: 1013

27.3. * people with T2DM

Inappropriate								Very Appropriate
1	2	3	4	5	6	7	8	9

Question ID: 1014

27.4. * other subgroups (Please make suggestions in the box provided.)

Inappropriate								Very Appropriate
1	2	3	4	5	6	7	8	9

Question ID: 984

28. Representativeness (how similar the people attending the service are to the local eligible population)

How representative of the entire eligible population (people with body mass in the overweight or obese range within that particular geographical area) the people attending the weight management service are.

Question ID: 985

Follow Up Instrument/Measurement/Presentation

Question ID: 986

28.1. * based on age of participants

Inappropriate								Very Appropriate
1	2	3	4	5	6	7	8	9

Question ID: 987

28.2. * based on sex of participants

Inappropriate								Very Appropriate
1	2	3	4	5	6	7	8	9

Question ID: 988

28.3. * based on BMI of participants

Inappropriate								Very Appropriate
1	2	3	4	5	6	7	8	9

Question ID: 989

28.4. * based on deprivation category of participants

Inappropriate								Very Appropriate
1	2	3	4	5	6	7	8	9

Question ID: 990

28.5. * based on ethnicity of participants

Inappropriate								Very Appropriate
1	2	3	4	5	6	7	8	9

Question ID: 991

28.6. * based on diabetes status of participants

Inappropriate								Very Appropriate
1	2	3	4	5	6	7	8	9

Question ID: 992

28.7. * based on other criteria (Please make suggestions in the box below)

Inappropriate								Very Appropriate
1	2	3	4	5	6	7	8	9

Question ID: 993

29. Prescription of Anti-obesity Medication

The number of participants taking drugs to help reduce or control their weight

Question ID: 994

Baseline Instrument/Measurement/Presentation

Question ID: 995

29.1. * % of participants on any anti-obesity medication

Inappropriate								Very Appropriate
1	2	3	4	5	6	7	8	9

Question ID: 996

29.2. * % of participants on specific anti-obesity medications

Inappropriate								Very Appropriate
1	2	3	4	5	6	7	8	9

Question ID: 997

Follow Up Instrument/Measurement/Presentation

Question ID: 998

29.3. * % of participants on anti-obesity medication

Inappropriate								Very Appropriate
1	2	3	4	5	6	7	8	9

Question ID: 999

29.4. * % of participants on specific anti-obesity medications

Inappropriate								Very Appropriate
1	2	3	4	5	6	7	8	9

Question ID: 1000

29.5. You may use this space to elaborate on your views in relation to any the above instruments/measurements. Please note to which of the above instruments/measurements your comment(s) relates.

Maximum length: 5000 characters. Characters left: 5000

Question ID: 1001

29.6. Please add any additional instruments/measurement(s) in the box below.

Maximum length: 5000 characters. Characters left: 5000

Thank you, please click Save to finish.

Save

Save to complete later

Next Page >

Supporting Information 10. Stage 2 (instrument selection), round 1 Delphi qualitative analysis.

Weight-related measurement/instruments

A couple of participants commented that all items listed were easy to calculate and capture in a database, although wminstf5666 singled out % of weight change as more indicative than actual weight lost. This person added:

"I have rated the 10% weight loss slightly less as it would depend on the length of the service as to the appropriateness of this i.e. how feasible is it that someone could achieve this level of weight loss in the length of time of the programme? I would suggest this is only included for longer term programmes."

Conversely, wminstd507f felt that all measures listed were meaningless unless information about loss to follow-up was provided when comparing services.

Six participants, mainly those working in weight management services (WMS), were unconvinced about measuring BMI < 25 or 30; they felt that, given the starting weight of most patients, this was unlikely to be achieved (unless they had undergone bariatric surgery). Furthermore, mean BMI was critiqued by two respondents due to the wide spread of weight among patients. Another person working for a WMS felt that BMI was no indication of functional impairment. Likewise, a sense of overlooking small improvements by focusing on specific BMI targets was highlighted:

"% participants achieving BMI<25 or <30 may underestimate the importance of smaller changes in weight and BMI." (wminst49c41)

One WMS staff member (wminst62c76) suggested that % of participants who had no change or gain in weight could be useful. Other additional areas for measurement mentioned were weight circumference (wminst17aad) and spread of participants across BMI bands pre and post intervention (wminstddcea).

Diabetes-related measurement/instruments

HbA1c was not considered by all as a fitting measure of outcome; it was seen as hard for WMS to perform this test. For some, it was regarded as an inappropriate assessment of improvements:

"HbA1c does not tell you retinopathy or neuropathy. In fact a sudden improvement in HbA1c levels can lead to blindness." (wminst62c76)

Conversely, one person felt that this was an important measure to capture for all patients, not just those with diabetes:

"Lowering glucose could be important in the general population of people with overweight and obesity - e.g. could move people from non-diabetic hyperglycaemia to normo glycaemia." (wminst49c41)

Alternatives to HbA1c were proposed by wminst62c76:

"Proteinuria (ACR) is better than HbA1c as if it improves then it suggests improved diabetic renal disease. It is a far better marker of improvement than HbA1c or other markers. Another easy marker would be HOMA2 score using c-peptide."

Collecting data on type 1 diabetes was not considered relevant by wminst0764f who stated it was not likely to be correlated with weight. Likewise, wminstff8ee commented that the majority of individuals attending a WMS were unlikely to have diabetes.

Differing views were expressed about gathering information on medication for diabetes:

"Medication changes only matter if there is a cost saving. So, far better to total up the cost of medication and see the change…currently there is a tendency to use more expensive medication and reduce the tablet burden." (wminst62c76)

"It would be interesting to see if there is any difference in Hba1c and medication (for type 2 diabetes) but our unit would find this difficult to do." (wminst0764f)

Quality of life measurement/instruments

Brevity and ease of completion of any quality of life (QoL) measure, for all stakeholders, shaped some people's responses, as implied in the following comment:

"I have rated them all at the lower end of the scale, I think questionnaire data is valuable but consideration needs to be given for the feasibility of this in a real life setting." (wminstf5666)

The issue of licence fee was also raised as potentially significant. Concerns were expressed about self-report measures being filled out in practice, adding complexity for the provider, and being potentially influenced by when they were completed:

“If they are administered by the weight management programme when the participant first attends a group for example, you aren't really getting a true indication of their baseline levels, i.e. they may have already sat though session one before answering the questionnaire which could alter their scores.” (wminstf5666)

A preference for weight-related QoL measures was expressed by wminste4557, seen as more sensitive to the aims of the service.

A couple of people stated being unacquainted with the tools listed in this section; this affected the ratings they were able to give:

“Not familiar with tools therefore have given a neutral score.” (wminstd5cd3)

Comments made about specific QoL tools included:

- The EQ5D being valued for measuring cost effectiveness, but too blunt an instrument
- The SF36 being too long
- The SF-12 possibly being difficult for service users to complete
- The PWI-ID being long, which could be an issue if several other instruments were used
- The “PI-ED for children” was depicted as appropriate when assessing services for children

One person commented about the need for more data on one, potentially useful instrument:

"The IWQoL is a nice measure, and I wish there was more research showing how this might relate to economic evaluation. As a measure of quality of life it is better and more sensitive." (wminst49c41)

Measuring adverse events/unintended consequences

It was unclear how a WMS would collect many of the outcomes listed, although injury from physical activity sessions was something that services were said to record as part of health and safety assessments. There was concern about having too many outcomes and this being onerous for providers, with some participants giving neutral ratings of outcomes as a consequence:

"I have marked 10.1 and 10.2 as mid range as I worry about trying to collect too much and then getting poor data." (wminst17aad)

Therefore, wminst1e5f5 wrote that a single, general outcome on adverse events was required. However, wminst62c76 seemed to disagree with this suggestion:

"You have to define pre-existing conditions and worsening. You cannot clump them all together as that is meaningless."

Three people remarked on the inability to link directly deterioration in a condition with weight, which also required patients to record such episodes. It was noted that *"most common side effect is through weight loss medication not being appropriately reviewed" (wminst510b2).*

Previous participation attempts were seen as important, to contextualise data. Data on repeat referrals were also seen as useful, if only to identify potentially inappropriate referrals, or to isolate services that might help with re-engagement. Other comments on this issue included the following:

"[It] is important as I know a service that screens patients and refuses to take them if not motivated. Those that were referred and not attended as screened out were never reported to the commissioners." (wminst62c76)

"% participants who have previously attended the service (attending at least 1 session)
% participants who have previously completed a course of the service (eg. attended >2/3 sessions of a treatment course)." (wminst49c41)

Measuring attendance and completion

A recurring refrain from respondents was that 100% attendance was unrealistic. Instead, a range of 70-90% was suggested to represent completion, although wminst62c76 expressed concern that providers may manipulate responses about completion. It was also noted by wminstda600 that: *"Arbitrary percentages for attendance are not evidence based associated to any outcome."* Furthermore, some people commented that improvements may ensue even if attendance was low, depending on patients' behaviours at home:

"…engagement in a programme outside of sessions can still be high. It is suggested that the team consider a minimum completion benchmark of 50%; this is a level which demonstrates engagement further to initial attendance and 2nd session attendance." (wminstddcea)

It was proposed by wminst49c41 that percentage of referred patients who attended 1 or more sessions was important, to provide information on how many people were referred but did not attend. Finally, wminstd507f asked: *"12.1 to 12.4 duplicate prior questions?"*

Measuring reasons for dropout

Many comments in this section related to the difficulty of finding out why people dropped out of a WMS, with patients tending not to give an explanation. In general, it was said to be impractical to expect services to gather such data. A specific aspect of this section was seen as especially appropriate by one person:

"Pregnancy I think is very important as fertility can increase with weight loss." (wminst0764f)

Measuring patient satisfaction

Some respondents were unfamiliar with the questionnaire listed in this section so struggled to provide a rating. It was noted by wminst510b2 that measures in this section were not appropriate for a WMS because few of these take place in hospital.

Measuring cost-effectiveness

Respondents were clear that providers should not be responsible for collecting data on cost-effectiveness, which was depicted as the role of commissioners. Accounting for complexity of patients attending a WMS was described as important when assessing cost-effectiveness. It was also noted that when evaluating this outcome, a focus on weight was insufficient:

"Cost of 'success' only around weight is very limiting. The patient may have improved mood, increased activity/mobility. Often a person my reduce more weight once the programme is completed - ie once they have the tools and life circumstances have changed for them." (wminst0764f)

"Uneasy with cost effectiveness being solely focussed on weight loss. Many patients are gaining weight when they enter services, so stabilisation may be success. Success could also be reflected in a multitude of other changes such as returned to work, reduced binge eating etc." (wminstff8ee)

Respondent wminst97fd0 gave some ideas for other areas of improvement in which WMS have an interest:

"MYMOP /MYMOP2 (measure yourself medical outcomes proforma). Records 2 most troublesome symptoms and improves with weight loss. Measures of physical fitness. Changes in diet quality. QoL measures eg IWQoL lite, EQ5D may also be helpful."

Finally, a policy maker noted that: *"A cost-utility analysis expressing cost per QALY is vital to enable the interventions to be assessed against other competing uses of resources." (wminst275b8)*

Presentation of results

Several comments related to a need to report outcomes for all, to enable comparison between attendance and non-attendance. For wminst62c76, this involved taking *"all patients beginning and end weight in program. If do not attend more than once then 0kg weight loss."* The requirement to be clear about how things like attendance and completion were defined was raised, including some flexibility in what constituted 'completion':

"…this would require a specific definition of completion and need to take into account expected reasonable completion rate eg allowance for a percentage of missed sessions for holiday for example within a given time frame, bad weather effecting accessibility etc." (wminst1a8ca)

For wminst97fd0, reporting on an intention treat basis, but also for those who actually attended, was suggested.

Reflections on optional measures

Three respondents commented on use of the oral glucose tolerance test (OGTT), feeling HbA1c was preferable and the test that tended to be used in primary care. General concern was expressed about the feasibility of gathering data on these outcomes due to records not being up-to-date, staff in WMS not having time and skills to collect these measures, and a risk of putting patients off with too many assessments. This is illustrated in the following remark from wminstf5666:

"I have rated all of these outcomes as inappropriate based on the fact that it would not be feasible to collect this information in the weight management programme itself. If it was possible to collect this

at the point of referral (from the primary care referrer/patient records) then this might add valuable insight to the service outcomes - but wouldn't be feasible to collect within the weight management programme itself."

Wminst3aec6 did not regard cardiovascular medication as important because patients tended to remain on these regardless of weight loss. This relates to the following comment from wminst97fd0:

"Assessment of CV risk is important and appropriate use of BP medication and lipid lowering medication is important based on risk. However, lipids are relatively insensitive to changes in weight so it is unlikely that lipid lowering medication can be reduced as a result of weight loss. BP medication and diabetes risk are important and more sensitive to weight change."

Overall measure of comorbidity

Respondents were clear that WMS were not set up to address mental health problems and, therefore, changes in these areas would not necessarily follow. Similarly, it was noted that WMS, if short-term, should not be expected to result in patients coming off medication for mental health problems:

"Weight loss treatment appears to have very modest effects on depression and anxiety that are not really worth measuring routinely." (wminstd1b39)

Again, the point was raised about patients not being overwhelmed by the number of measures they had to complete. For wminst0764f, only one measure of mental health was needed. Others had

views on the suitability of specific instruments presented. The BDI was seen as having cost implications and being lengthy, and the PHQ was said to have the risk of suicide disclosure:

"Suggest PHQ8 rather than PHQ9. This avoids raising the issue of suicidal thinking and placing additional strain on providers who are not trained to deal with 'yes' responses." (wminstd507f)

"The PHQ asks specifically about risk in q9 - clinicians need to be trained to know how to deal with responses to this, and need to follow up any indications of risk - in a clinical setting this could be problematic and leave clinicians vulnerable if the process by which to do this is not in place." (wminste4557)

Respondent wminst62c76 argued that functional measures should be included:

"None of the co-morbidity scores adequately measure functional status. I suggest ECOG and how many flight of stairs they can manage."

Measures of self-esteem, self-confidence, disordered eating, importance of weight loss

A repeated message from respondents in this section was about ensuring measurement was not too cumbersome for patients or providers. Hence, comments were made about questionnaires having to be short and simple to complete. Furthermore, wminst97fd0 observed that many of the listed instruments were developed for research and may not be practical for use in services. Remarking on specific instruments, the Tennesse Self-concept Scale Score and the General Well-being Schedule Score were reported by wminst0764f as too lengthy. A couple of respondents gave more extended comments:

"These questionnaires all have different strengths and weaknesses. I feel the EDEQ is more targeted at people of a healthy weight with disordered eating, so many of the questions do not feel appropriate. Using a department's own measure would not have benefit as the way in which this is measured would vary so hugely. The QEWP also focused significantly on compensatory behaviours, which are not particularly common in our client group. The TFEQ seems better, although also has its weaknesses." (wminste4557)

This seemed to be contrary, in some respect, to what was written by wminst49c41:

"I rated the TFEQ as less appropriate as it doesn't measure disordered eating per se. Measuring restraint and disinhibition in studies is useful as it can help us to understand changes in eating behaviour and identify predictors of success. However, if we are thinking about core outcomes in weight management services, it would be more appropriate to have a measure of bingeing and/or emotional eating, as these would have more direct implications for treatment based on current knowledge of treatment success."

It was noted that WMS are not designed to address eating disorders, although improvements in behaviours like binge eating may occur. However, wminst0764f felt this information could be useful for highlighting additional needs in terms of eating disorder support. Two participants said they were not familiar with many of the measures in this section, so found it hard to make a judgment.

Reach and representativeness measures

In this section, people put forward additional areas to measure, including:

- Language (non-English)
- Internet access
- Income
- Chronic remitting diseases
- Mobility problems
- Location – geography
- Non-diabetic hyperglycaemia

Measurement of anti-obesity medication

This was not seen by some as important: a) because only one drug was licenced within this country, b) present use of such medication was low.

Supporting Information 11. Stage 2 (instrument selection), round 2 Delphi questionnaire as it appeared to participants.

Question ID: 1129

1. * I have read the Invite and Information Letter (v3.0 07/09/17) and I consent to participate in this Delphi process to select tools/instruments to measure core outcomes for lifestyle weight management. I know that my free text comments will be analysed and may be quoted in publications arising from this work. (You have to tick YES to participate in the rest of the questionnaire.)

- **Yes**
- **No**

Question ID: 1130

2. * I consent to being named as a member of this development group in the acknowledgements of any publication arising from this work. (OPTIONAL)

- **Yes**
- **No**

Question ID: 1131

Core

Following the first three rounds of this Delphi process, the outcomes below have been identified as being core for measurement by weight management services.

Please either rank the corresponding instruments/measurements in terms of their appropriateness for use or select the most appropriate instrument/measurement for use, as instructed.

Question ID: 1132

3. Weight

The measurement of how heavy a participant is in kilograms (kg) or stones and pounds

All four follow up options for this outcome scored very highly in round 1. It may be that a combination of them is the best approach. We have now included additional options to this effect.

Question ID: 1134

Please **rank** the 7 options provided (3.1-3.7) from 1-7 in terms of their appropriateness for use, where 1 is the most appropriate and 7 is the least appropriate

Question ID: 1135

3.1. * mean change in participants' weight in kg

Most Appropriate | Least Appropriate

1	2	3	4	5	6	7

Question ID: 1136

3.2. * mean % weight change of participants

Most Appropriate | Least Appropriate

1	2	3	4	5	6	7

Question ID: 1137

3.3. * % of participants achieving ≥ 5% weight loss

Most Appropriate | Least Appropriate

1	2	3	4	5	6	7

Question ID: 1138

3.4. * % of participants achieving ≥ 10% weight loss

Most Appropriate | Least Appropriate

1	2	3	4	5	6	7

Question ID: 1139

3.5. * all of the above measurements (3.1 + 3.2 + 3.3 + 3.4)

Most Appropriate | Least Appropriate

1	2	3	4	5	6	7

Question ID: 1140

3.6. * measurements 3.2 + 3.3 (mean % weight change + % achieving ≥ 5% weight loss)

Most Appropriate | Least Appropriate

1	2	3	4	5	6	7

Question ID: 1141

3.7. * measurements 3.3 + 3.4 (% achieving ≥ 5% weight loss + % achieving ≥ 10% weight loss)

Most Appropriate | Least Appropriate

1	2	3	4	5	6	7

Question ID: 1142

3.8. You may use this space to elaborate on your views in relation to any of the above instruments/measurements. Please note to which of the above instruments/measurements your comment(s) relates.

Maximum length: 5000 characters. Characters left: 5000

Question ID: 1143

4. Completion

How many people finished the weight management programme

All three follow up options for this outcome obtained the same score in round 1, despite free text comments suggesting that 100% was unrealistic and somewhere between 70-90% was more appropriate.

This completion definition is vital to allow intention-to-treat and completer-only analyses of weight loss results.

Question ID: 1145

4.1. * Please **select** the most appropriate option.

- **% of participants who attended 100% of possible/core/mandatory sessions**
- **% of participants who attended 80% of possible/core/mandatory sessions**
- **% of participants who attended 70% of possible/core/mandatory sessions**

Question ID: 1149

4.2. You may use this space to elaborate on your views in relation to any of the above instruments/measurements. Please note to which of the above instruments/measurements your comment(s) relates.

Maximum length: 5000 characters. Characters left: 5000

Question ID: 1150

5. Participant Satisfaction

How happy/satisfied participants were with the weight loss service.

Various questionnaires can be used to obtain a participant satisfaction score. Information on each questionnaire can be found in Document 1.

Both follow up options for this outcome obtained the same score in round 1 despite being quite different.

While the longer OEQ will give the service helpful information for service improvement, FFT is short and comparable across other services. The OEQ will have to be modified slightly to work for a weight management service. We have done this and attached the modified version for you to see.

Question ID: 1155

5.1. * Please **select** the most appropriate option.

- **mean Outcomes and Experiences Questionnaire (OEQ) score adapted to suit weight management services**
- **mean NHS Friends and Family Test (FFT) score**

Question ID: 1190

5.2. You may use this space to elaborate on your views in relation to any of the above instruments/measurements. Please note to which of the above instruments/measurements your comment(s) relates.

Maximum length: 5000 characters. Characters left: 5000

Question ID: 1154

6. Cost Effectiveness

The value for money of the weight management service in terms of long-term economic benefits to the NHS.

All three possible measures of this outcome received similar scores in round 1.

Free text comments suggested that cost effectiveness should not be based on weight alone. However, every single health economic tool for obesity interventions is driven by weight change and it is not at all feasible for us to develop a new health economic model for this purpose.

Also, do not let the issue of who would complete the analysis affect your decision at this point as all three measures are simple to calculate using core outcomes already covered.

Question ID: 1157

6.1. * Please **select** the most appropriate option.

- **The Public Health England Weight Management Economic Assessment Tool [1]**
- **cost / kg (based on mean weight loss)**
- **cost per 'success' with success being 5% weight loss**

Question ID: 1191

[1]The Public Health England Weight Management Economic Assessment Tool:
http://webarchive.nationalarchives.gov.uk/20170110165804/http://www.noo.org.uk/visualisation/economic_assessment_tool

Question ID: 1161

6.2. You may use this space to elaborate on your views in relation to any of the above instruments/measurements. Please note to which of the above instruments/measurements your comment(s) relates.

Maximum length: 5000 characters. Characters left: 5000

Question ID: 1162

7. Presentation of Results

Which participants' outcomes to include in reporting

The scores that the options for this outcome received in round 1 contradicted the free text comments given. Comments suggested that services should not only report results for those completing the programmes but for everyone who attends. It may be that what was intended by the scores in round 1 was that services should report weight change in completers PLUS in a larger group reflecting the total cohort. Grouped options have been provided to reflect this, in addition to a description of how the data could be handled for the various options.

Question ID: 1164

Please **rank** the 5 options provided (7.1-7.5) from 1-5 in terms of their appropriateness for use, where 1 is the most appropriate and 5 is the least appropriate.

Question ID: 1165

7.1. * Report outcomes for all participants attending ≥ 1 active weight loss session(s) (does not include introductory sessions/information sessions about the service). Those who were referred but did not attend and those who only attended once would be recorded as 0 kg weight change.

Most Appropriate | Least Appropriate

1	2	3	4	5

Question ID: 1166

7.2. * Report outcomes for all participants attending > 1 active weight loss session(s) and therefore having weight change data (does not include introductory sessions/information sessions about the service). All of those who were referred but did not attend, in addition to those who only attended once, would not be counted in the weight change data and would be reported as a separate group of 'referred but did not attend > 1 session' instead.

Most Appropriate | Least Appropriate

1	2	3	4	5

Question ID: 1167

7.3. * Report outcomes for all participants completing the programme.

Most Appropriate | Least Appropriate

1	2	3	4	5

Question ID: 1168

7.4. * Report 7.1 + 7.3

Most Appropriate | Least Appropriate

1	2	3	4	5

Question ID: 1169

7.5. * Report 7.2 + 7.3

Most Appropriate | Least Appropriate

1	2	3	4	5

Question ID: 1170

7.6. You may use this space to elaborate on your views in relation to any of the above instruments/measurements. Please note to which of the above instruments/measurements your comment(s) relates.

Maximum length: 5000 characters. Characters left: 5000

Question ID: 1171

OPTIONAL

Following the first three rounds of this Delphi process, the outcomes below have been identified as being optional for measurement by weight management services.

Please select the most appropriate instrument/measurement for each outcome.

Question ID: 1172

8. Overall Measure of Comorbidity

Measure of the presence of additional diseases or disorders co-occurring with obesity/being overweight

Various indexes or scoring systems can be used to obtain a measure of comorbidity. Information on each can be found in Document 2.

All possible options for this outcome received low ratings in round 1.

This is an optional outcome so service and participant burden is less of an issue. As it was selected as an optional outcome in the first phase of this project, please help us to decide which instrument/measurement we should recommend (for use at both baseline and follow up) when there is a desire to quantify comorbidity.

Question ID: 1174

8.1. * Please **select** the most appropriate option.

- **mean CCI score**
- **mean EOSS score**
- **mean Chronic Disease Score**
- **mean number of dispensed medications per participant**
- **I have insufficient knowledge of the instruments and am therefore unable to select one.**

Question ID: 1189

8.2. You may use this space to elaborate on your views in relation to any of the above instruments/measurements. Please note to which of the above instruments/measurements your comment(s) relates.

Maximum length: 5000 characters. Characters left: 5000

Question ID: 1175

9. Depression

Whether a participant suffers from a mental illness characterised by a profound and persistent feeling of sadness or despair and/or a loss of interest in things that once were pleasurable.

Various questionnaires can be used to obtain a depression score. Information on each questionnaire can be found in Document 3.

All possible options for this outcome received fairly modest scores in round 1.

This is an optional outcome so service and participant burden is less of an issue. As it was selected as an optional outcome in the first phase of this project, please help us to decide which instrument/measurement we should recommend (for use at both baseline and follow up) when there is a desire to quantify depression.

Question ID: 1177

9.1. * Please **select** the most appropriate option.

- **mean HADS questionnaire score of participants**
- **mean PHQ9 questionnaire score of participants**
- **mean Beck Depression Inventory score of participants**

Question ID: 1178

9.2. You may use this space to elaborate on your views in relation to any of the above instruments/measurements. Please note to which of the above instruments/measurements your comment(s) relates.

Maximum length: 5000 characters. Characters left: 5000

Question ID: 1179

10. Importance of Weight Loss

How important participants feel it is for them to lose weight

Various scales can be used to obtain an importance of weight loss/dieting readiness score. Information on each scale can be found in Document 4.

All possible options for this outcome received fairly low scores.

This is an optional outcome so service and participant burden is less of an issue. As it was selected as an optional outcome in the first phase of this project, please help us to decide which instrument/measurement we should recommend (for use at both baseline and follow up) when there is a desire to quantify readiness/ self-efficacy related to weight loss.

Question ID: 1181

10.1. * Please **select** the most appropriate option.

- **mean Dieting Readiness Scale score(s)**
- **mean DIET score(s)**
- **mean Self-Efficacy for Eating Behaviours Scale score(s)**

Question ID: 1182

10.2. You may use this space to elaborate on your views in relation to any of the above instruments/measurements. Please note to which of the above instruments/measurements your comment(s) relates.

Maximum length: 5000 characters. Characters left: 5000

Thank you, please click Save to finish.

Save

Prev 1 2 3 4 ... 7 8 Next

Save to complete later

Next Page >

Supporting Information 12. Stage 2 (instrument selection), round 2 Delphi qualitative analysis.

Weight-related outcomes

Mean % weight change was seen by some as helpful because it was proportional, although wminstd507f felt that its use resulted in a loss of data, especially if a significant proportion (e.g. 40%) of people were not followed up. It was also noted by wminst510b2 that mean % weight change could be skewed by outliers. Concern was expressed that use of 5% or 10% weight loss was not based on sound evidence; they were referred to as subjective or arbitrary thresholds:

“There's nothing special about 5% and 10% weight loss, they've become markers but with no rationale for them.” (wminstd1b39)

One person commented that the format of the questionnaire prevented them from expressing their view:

"PLEASE note I would rank all 7 but it wouldn’t let me rank more than 2 the same!!! As part of a health behaviour change we would not use weight as a measurement of improvement. Improvements in blood lipids and HBa1C are used instead as well as WEMBS mental well being…” (wminstd5efb)

Completion-related outcomes

Several people mentioned that 100% completion was unrealistic. Therefore, 70% or 75% were considered a good compromise. Respondent wminstda600 noted that number of sessions completed was not necessarily related to outcomes, an observation contradicted by another person:

"Across our weight management programmes we have successfully improved outcomes in those who have attended 70%+ of our sessions." (wminst510b2)

For others, completion rates were seen as arbitrary and not necessarily reflective of how programmes were set up (e.g. they might include weight loss and then a maintenance phase; they could have core and extra requested sessions).

Patient satisfaction outcomes

Comments made in this section related to selecting the measure that was quickest to complete. A positive remark about the Friends and Family Test was it is something that tends to be gathered as a routine part of care. However, wminstd1b39 stated it was too UK-centric for more general use, and wminsteb420 wrote that is was not appropriate for children (suggesting that the PI-ED may be better for this age group). One person was unhappy with either option:

"I feel like neither are ideal...is there a shortened version of the OEQ perhaps?" (wminst49c41)

Cost-effectiveness outcomes

Cost/kg was referred to by some respondents as a simple outcome to measure. One person gave a very clear rationale for selecting this option:

"5% is not a success, it's just an arbitrary point on a continuous scale. Internationally, studies are not going to use an English scale. Cost/kg is easy to calculate and highly practical." (wminstd1b39)

Others remarked that cost-effectiveness is a more long-term consideration:

"The 5% weight loss is clinically effective and the cost per success is met by continuing to achieve that weight loss maintenance over a longer period i.e. 12 months and beyond." (wminst510b2)

"…using the cut off of 5% can be a challenge when you have short term programmes and groups of people who have larger BMIs." (wminstddcea)

Presentation of results

This section focused on the need to treat those not attending, and those attending and not losing weight, as distinct groups when presenting results. It was suggested that for those not attending, information about inappropriate referrals could be garnered. It was also stated by wminstd507f that clarity was required about what was meant by 'completing the programme' - *"…is that all sessions?"*

Overall measure of comorbidity

Only two comments were made in this section, both by staff working in weight management programmes, which related to how these services are run and data that is used by practitioners:

"I would report numbers and percentages of people in each EOSS score group as well, this score is widely used and includes functional abilities." (wminst1e5f5)

"We do look at chronic disease and medications but this is done with our internal clinical medical team to ensure that it is operationally viable and meets local strategic needs." (wminst510b2)

Measuring depression

Brevity and simplicity of how questions are phrased informed respondents' decisions in the option they selected. However, this led some people to choose the HADS, whilst others picked the PHQ9 for this reason. One person (wminstd507f) wrote they would prefer the PHQ8 because it removed the question about suicide.

Importance of weight loss to service users

Several people commented on being unsure how to answer this section because they a) were unfamiliar with the measures but were forced to select at least one, and b) questioned why such information should be collected. Hence, some people said they would have preferred not to answer this question:

“I don’t feel I can answer this question as I only have the first option in front of me at this moment. I have chosen the middle one only because I was forced into a decision.” (wminst92f98)

The use of an 89-item self-efficacy scale was queried by a couple of respondents. Likewise, one individual proposed a simpler way of measuring the importance of weight loss to service users:

“Sometimes it is just easier to use a 7-point Likert Scale with the questions ‘How ready are you to make the changes to your diet & lifestyle to lose weight?’” (wminst62c76)

Supporting Information 13. Stage 2 (instrument selection), round 3 Delphi questionnaire as it appeared to participants.

1. * I have read the Invite and Information Letter (v3.0 07/09/17) and I consent to participate in this Delphi process to select tools/instruments to measure core outcomes for lifestyle weight management. I know that my free text comments will be analysed and may be quoted in publications arising from this work. (You have to tick YES to participate in the rest of the questionnaire.)

- Yes
- No

2. * I consent to being named as a member of this development group in the acknowledgements of any publication arising from this work. (OPTIONAL)

- Yes
- No

We will now present the findings for CORE and OPTIONAL outcomes. At the end of these lists you will be asked whether or not you accept the findings of the expert group.

CORE

Following the first two rounds of this instrument selection Delphi process, the instruments/measurements listed below have been identified by the expert group as being most appropriate for use. In some cases, there was no definitive choice of instrument/measurement so more than one has been identified as appropriate for use. All instruments included should be used and all measurements included should be reported, unless otherwise indicated.

3. Age

How old participants are/the age (in years) of participants

Instrument/Measurement/Presentation To Be Used/Reported At Baseline

- mean age of participants in years
- % of participants in age bands (16-24 years, 25-34 years, 35-44 years, 45-54 years, 55-64 years, 65-74 years, 75+ years)

4. Weight

The measurement of how heavy a participant is in kilograms (kg) or stones and pounds

Instrument/Measurement/Presentation To Be Used/Reported At Baseline

- mean weight of participants in kg

Instrument/Measurement/Presentation To Be Used/Reported At Follow Up

- mean change in participants' weight in kg
- mean % weight change of participants
- % of participants achieving ≥ 5% weight loss
- % of participants achieving ≥ 10% weight loss

5. Body Mass Index (BMI)

An approximate measure of whether a participant is overweight or underweight, calculated by dividing their weight in kilograms by the square of their height in metres

Instrument/Measurement/Presentation To Be Used/Reported At Baseline

- mean BMI of participants
- % of participants in BMI categories < 25, 25-29.9, 30-34.9, 35-39.9, 40-49.9, 50-59.9, ≥ 60

Instrument/Measurement/Presentation To Be Used/Reported At Follow Up

- mean change in participants' BMI

6. Diabetes Status

Whether a participant has diabetes, a condition which occurs when the body doesn't produce enough insulin to function properly, or the body's cells don't react to insulin. This means glucose stays in the blood and isn't used as fuel for energy. Type 2 diabetes is often associated with obesity and an increased risk of developing cardiovascular disease.

Instrument/Measurement/Presentation To Be Used/Reported At Baseline

- % of participants with type 2 diabetes mellitus (based on self -report, case record or blood test)

Instrument/Measurement/Presentation To Be Used/Reported At Follow Up

- mean change in HbA1c levels of those participants with T2DM

7. Quality of Life (QoL) Score

A measure of the general well-being of participants.
Various questionnaires can be used to obtain a quality of life score.

Instrument/Measurement/Presentation To Be Used/Reported At Baseline

- mean EQ-5D-5L scores of participants
- mean EQ-5D-5L scores of participants

8. Learning Disability QoL Score

A measure of the general well-being of participants with a learning disability.
Various questionnaires can be used to obtain a quality of life score.

Instrument/Measurement/Presentation To Be Used/Reported At Baseline

- mean PWI-ID score(s) of participants

Instrument/Measurement/Presentation To Be Used/Reported At Follow Up

- mean PWI-ID score(s) of participants

9. Adverse Events/Unintended Consequences

Whether participants suffered any unfortunate side effects as a result of attending the weight loss service.

Instrument/Measurement/Presentation To Be Used/Reported At Follow Up

number of participants experiencing a worsening of a pre-existing medical condition, such as

- an undiagnosed eating disorder
- other pre-existing medical conditions
- number of participants sustaining an injury during a physical activity session run by the weight management service

10. Repeat Referrals

Whether a participant has been referred to the weight management service on more than one occasion.

Instrument/Measurement/Presentation To Be Used/Reported At Follow Up

- % of participants previously referred to the service, not necessarily having attended any sessions)
- % of participants answering yes, having previously attended at least 1 weight management session

11. Attendance

How many people attended the weight management service

Instrument/Measurement/Presentation To Be Used/Reported At Follow Up

- mean % of core/mandatory sessions attended by participants

12. Completion

How many people finished the weight management programme

Instrument/Measurement/Presentation To Be Used/Reported At Follow Up

- % of participants who attended 80% of possible/core/mandatory sessions

13. Reason for Dropout

Why those participants who did not complete the programme failed to do so.

Instrument/Measurement/Presentation To Be Used/Reported At Follow Up

% of participants who dropped out due to:

- dissatisfaction with the intervention (unrelated to weight loss)
- poor weight loss
- illness/ hospitalisation
- pregnancy
- change in personal circumstances/social reason
- moving from the geographical area
- any other reason
- unknown reason

14. Participant Satisfaction

How happy/satisfied participants were with the weight loss service.
Various questionnaires can be used to obtain a participant satisfaction score.
In this instance, the weight management service should select the questionnaire/method they feel is most appropriate for their use.

Instrument/Measurement/Presentation To Be Used/Reported At Follow Up

Comments and scores indicate that neither of the suggested instruments for measuring patient satisfaction is ideal. Therefore, it is proposed that no instrument is selected. The two options below will be given as suggestions but other methods could be used.

- mean Outcomes and Experiences Questionnaire (OEQ) score adapted to suit weight management services
- mean NHS Friends and Family Test (FFT) score

15. Cost Effectiveness

The value for money of the weight management service in terms of long term economic benefits to the NHS.

Instrument/Measurement/Presentation To Be Used/Reported At Follow Up

- The Public Health England Weight Management Economic Assessment Tool
http://webarchive.nationalarchives.gov.uk/20170110165804/http://www.noo.org.uk/visualisation/economic_assessment_tool

16. Presentation of Results

Which participants' outcomes to include in reporting

Instrument/Measurement/Presentation To Be Used/Reported At Follow Up

- report outcomes for all participants attending >1 active weight loss session(s) and therefore having weight change data (does not include introductory sessions/information sessions about the service)
- report outcomes for all participants completing the programme

OPTIONAL

Following the first two rounds of this instrument selection Delphi process, the instruments/measurements listed below have been identified by the expert group as being most appropriate for use. In some cases, there was no definitive choice of instrument/measurement so more than one has been identified as appropriate for use. All instruments included should be used and all measurements included should be reported, unless otherwise indicated.

17. High Blood Pressure

Whether a participant has high blood pressure. High blood pressure increases the risk of developing cardiovascular disease.

Instrument/Measurement/Presentation To Be Used/Reported At Baseline

- % of participants with high blood pressure based on patient report/medication/case notes
- % of participants with high blood pressure based on blood pressure readings

Instrument/Measurement/Presentation To Be Used/Reported At Follow Up

- change in % of individuals with blood pressure above current recommended treatment thresholds (i.e. normotensive or adequately treated)

18. Blood Pressure

The pressure of blood in the arteries, the vessels that carry blood from the heart to the rest of the body. A certain amount of pressure is required to get the blood around the body but consistently high blood pressure increases the risk of cardiovascular disease.

Instrument/Measurement/Presentation To Be Used/Reported At Baseline

- mean systolic and diastolic blood pressure of participants

Instrument/Measurement/Presentation To Be Used/Reported At Follow Up

- change in mean systolic and diastolic blood pressure of participants

19. Cardiovascular Risk

A measure of how likely participants are to develop cardiovascular disease, including heart disease and stroke

Instrument/Measurement/Presentation To Be Used/Reported At Baseline

- % of participants with previous cardiovascular disease (CVD), including myocardial infarction, stroke, transient ischaemic attack (TIA), angina and peripheral vascular disease
- % of participants with high CVD risk (previous CVD or a high cardiovascular risk score - N.B. information on blood pressure and lipids would be required to calculate the risk score)
- % of participants with a high cardiovascular risk score (primary prevention/not those with previous cardiovascular disease)
- mean CVD risk score of participants (primary prevention/not those with previous cardiovascular disease)

Instrument/Measurement/Presentation To Be Used/Reported At Follow Up

- % of participants with a high cardiovascular risk score (primary prevention/not those with previous cardiovascular disease)
- change in mean cardiovascular risk score of participants (primary prevention/not those with previous cardiovascular disease)

20. High Cholesterol/ Lipids

A measure of whether a participant has an abnormal amount of fat and/or cholesterol, known as lipids, in their blood (also called dyslipidaemia). Being overweight can increase the likelihood of developing dyslipidaemia. Dyslipidaemia is associated with increased risk of developing cardiovascular disease.

Instrument/Measurement/Presentation To Be Used/Reported At Baseline

- % of participants with high cholesterol/lipids based on self-report /case records
- mean total cholesterol/ high density lipoprotein/ triglycerides of participants as obtained via blood test

Instrument/Measurement/Presentation To Be Used/Reported At Follow Up

- mean total cholesterol/ high density lipoprotein/ triglycerides of participants as obtained via blood test

21. High Future Risk of Diabetes (Impaired Fasting Glucose, Impaired Glucose Tolerance, Raised HbA1c Levels, Previous Gestational Diabetes)

Whether measures of the amount of glucose in a participant's blood suggests he/she is likely to develop type 2 diabetes in the future.

Instrument/Measurement/Presentation To Be Used/Reported At Baseline

- % of participants with a medical record of high diabetes risk (HDR) as determined by measuring HbA1c/fasting glucose/Oral Glucose Tolerance Test (OGTT) (either measured during intervention or in medical records)

Instrument/Measurement/Presentation To Be Used/Reported At Follow Up

- % of all participants with HDR as determined by measuring HbA1c/fasting glucose/OGTT (either measured during intervention or in medical records)
- % of those participants identified as having HDR at baseline who still have HDR (as determined by measuring HbA1c/fasting glucose/OGTT) , normoglycemia or type 2 diabetes

22. Overall Measure of Comorbidity

Measure of the presence of additional diseases or disorders co-occurring with obesity/being overweight
Various indexes or scoring systems can be used to obtain a measure of comorbidity.

Instrument/Measurement/Presentation To Be Used/Reported At Baseline

- mean EOSS score

Instrument/Measurement/Presentation To Be Used/Reported At Follow Up

- mean EOSS score

23. Depression

Whether a participant suffers from a mental illness characterised by a profound and persistent feeling of sadness or despair and/or a loss of interest in things that once were pleasurable.
Various questionnaires can be used to obtain a depression score.

Instrument/Measurement/Presentation To Be Used/Reported At Baseline

- % of participants with depression based on patient report/medication/case notes
- % of participants on medication for depression
- mean PHQ9 questionnaire score of participants

Instrument/Measurement/Presentation To Be Used/Reported At Follow Up

- % of all participants on medication for depression
- % of those patients identified as having depression at baseline on medication for depression
- mean PHQ9 questionnaire score of participants

24. Self-confidence & Self-esteem

How participants feel about their own abilities and worth
Various questionnaires can be used to obtain a self-confidence/self-esteem score and a measure of general well-being

Instrument/Measurement/Presentation To Be Used/Reported At Baseline

- mean WEMWBS score

Instrument/Measurement/Presentation To Be Used/Reported At Follow Up

- mean WEMWBS score

25. Importance of Weight Loss

How important participants feel it is for them to lose weight
Various scales can be used to obtain an importance of weight loss/dieting readiness score.

Instrument/Measurement/Presentation To Be Used/Reported At Baseline

- mean Dieting Readiness Scale score(s)

Instrument/Measurement/Presentation To Be Used/Reported At Follow Up

- mean Dieting Readiness Scale score(s)

26. Disordered Eating

Whether participants have disturbed and unhealthy eating patterns that can include restrictive dieting, compulsive eating or skipping meals. Disordered eating can include behaviours which reflect many but not all of the symptoms of feeding and eating disorders such as anorexia nervosa, bulimia nervosa and binge eating disorder.

Instrument/Measurement/Presentation To Be Used/Reported At Baseline

- % of participants with disordered eating (defined as per service)

Instrument/Measurement/Presentation To Be Used/Reported At Follow Up

- % of participants with disordered eating (defined as per service)

27. Reach (% eligible population who are referred to/take up weight management service)

The percentage of the eligible population (people who are overweight or obese within that particular geographical area) referred to the weight management service.

Instrument/Measurement/Presentation To Be Used/Reported At Follow Up

For a specific population subgroup of concern, what % of that population has been referred to/ attended the weight management service. Local data (e.g. Quality and Outcomes Framework) can be used to obtain prevalence rates. Population subgroups of interest:

- age <30
- male
- people with T2DM
- other subgroups

28. Representativeness (how similar the people attending the service are to the local eligible population)

How representative of the entire eligible population (people with body mass in the overweight or obese range within that particular geographical area) the people attending the weight management service are.

Instrument/Measurement/Presentation To Be Used/Reported At Follow Up

- based on age of participants
- based on sex of participants
- based on BMI of participants
- based on deprivation category of participants
- based on ethnicity of participants
- based on diabetes status of participants
- based on the geographical spread of the home addresses of participants

29. Prescription of Anti-obesity Medication

The number of participants taking drugs to help reduce or control their weight

Instrument/Measurement/Presentation To Be Used/Reported At Baseline

- % of participants on any anti-obesity medication (total and by class/medication)

Instrument/Measurement/Presentation To Be Used/Reported At Follow Up

- % of participants on anti-obesity medication (total and by class/medication)

30. * Do you accept these findings of the expert group?

- Yes
- No

31. If you do not accept the findings of the expert group and feel very strongly that a particular instrument(s)/measurement(s) should be excluded from either the core list or the optional list, please provide an explanation as to why in the box below. All suggestions will be given due consideration.

Maximum length: 5000 characters. Characters left: 5000

Thank you, please click Save to finish.

Save

Save to complete later

Next Page >

Supporting Information 14. Stage 2 (instrument selection), round 3 Delphi qualitative analysis.

Most open comments came from WMS staff. They tended to couch their response by stating that they partially accepted the results (rather than rejecting them wholesale). The key issue that seemed to concern these participants related to measures of diabetes status. They questioned whether there was capacity in services to perform necessary medical tests, who would fund these, and potential burden that may be placed on general practice as a consequence:

> *"…how will a follow up HBA1c be collected - this would have to be done within the health service but how will the patient be called into primary care to have this measured and would primary care be happy to take on this resource and fund the blood test?" (wminst1a8ca)*

Lack of consistency in measuring this outcome was also raised:

> *"If type 2 diabetes status (Yes or No), from self-report, case record or blood test are indicated for baseline, it is not recommended that HbA1c levels for those with T2DM are used at follow up. It does not allow for pre/post comparisons..." (wminstddcea)*

> *"If there is no baseline measure of HBA1c to compare against, what will follow up measures mean in terms of outcomes and impact of the programme?" (wminst1a8ca)*

For wminst62c76, diabetes or high risk of the condition at follow-up should be assessed by measuring 5% or 10% of weight loss. In relation to weight, wminst1a8ca asked why % of participants in each BMI range was only recorded at baseline:

"…recording % participants in each BMI range at follow up would provide insight into the number of patients reducing health risk which could be informative if, for example, the mean change is skewed by some large individual changes in BMI." (wminst1a8ca)

Certain WMS staff critiqued proposed items for measurement for failing to correspond with clinical outcomes. This included mean change in depression, self-confidence and importance of weight loss:

"They may be nice for psychology degree audits, but do not correlate well to any long term clinical outcomes." (wminst62c76)

Specific additional comments were made by wminst62c76 about a) completion rates being a better measure than attendance at follow-up, b) measuring number of people on statins at baseline and follow-up, rather than cholesterol at follow-up, and c) cost-effectiveness having to be *"decided by the CCGs/commissioners."*

Aside from WMS staff, just one member of another group, an academic, made a written response, commenting on the use of the PHQ9 because:

"…items 3, 4, 5 and 8 are so heavily influenced by issues in this population (sleep apnoea, and large body mass). I think people will come out with artificially elevated scores on this measure and would be concerned about the potential for over-prescription of anti-depressants which often have a negative effect on weight. Having normative data for use of the PHQ-9 in an obese population would be extremely helpful in this matter." (wminste4557)

Furthermore, this individual suggested that guidance on how to measure eating disorder behaviours was required, a comment reiterated by wminst0764f.

Supporting Information 15. Expert group members.

The authors wish to thank all expert group members. For the stage 1 (outcome selection) Delphi process, 36 of the 38 experts who completed round 1 consented to being named and are listed below. For the stage 2 (instrument/definition selection) Delphi process, all 33 experts who completed round 1 consented to being named and are listed below.

Stage 1 Delphi Experts

Professor Peymane Adab, Dr Amy Ahern, Ms Anita Attala, Dr Esme Banting, Mr Kenneth Barr, Ms Anna Bell-Higgs, Dr David Blane, Mr Mickey Brannigan, Mr Angus Deas, Ms Julie Edgar, Dr Emma Frew, Ms Ilene Gorman, Ms Zoe Griffiths, Dr Ewan Hamnett, Dr Carly Hughes, Ms Michaela James, Mr David Jones, Dr Lowri Kew, Dr Rhodri King, Dr Dimitrios Koutoukidis, Dr Jacquie Lavin, Dr Susan Legge, Ms Gemma Mann, Dr Helen Moffat, Ms Cath Morrison, Mrs Lauren Ness, Ms Catherine Parker, Dr Helen Parretti, Ms Sarah Preston, Mr Michael Roberts, Ms Laura Roche, Ms Deb Smith, Ms Jo Smith, Dr Abd Tahrani, Dr Deborah White, Ms Julie Whitham

Stage 2 Delphi Experts

Professor Peymane Adab, Dr Amy Ahern, Ms Kate Anderson, Ms Anita Attala, Professor Alison Avenell, Professor Paul Aveyard, Dr Emma Baldry, Ms Jacqualin Barron, Ms Anna Bell-Higgs, Ms Dale Carter, Dr Emma Frew, Ms Ann Grant, Professor Colin Greaves, Ms Zoe Griffiths, Dr Carly Hughes, Dr David Hughes, Ms Michaela James, Dr Rhodri King, Dr Dimitrios Koutoukidis, Ms Jeanette Lamb, Dr Jacquie Lavin, Ms Gemma Mann, Ms Louise McCombie, Dr Helen Moffat, Ms Cath Morrison, Ms Carolyn Pallister, Ms Hilary Pierce, Ms Sarah Preston, Ms Jo Smith , Dr Rachel Strachan, Dr Abd Tahrani, Ms Joanna Teece, Professor John Wilding

Table S1 - COS-STAR Statement completed checklist (from Kirkham et al.[1]).

SECTION/TOPIC	ITEM No.	CHECKLIST ITEM	OUR PAPER
TITLE/ABSTRACT			
Title	1a	Identify in the title that the paper reports the development of a COS	see title
Abstract	1b	Provide a structured summary	see abstract
INTRODUCTION			
Background and objectives	2a	Describe the background and explain the rationale for developing the COS	paragraphs 1-3 provide background and paragraph 4 explains rationale
	2b	Describe the specific objectives with reference to developing a COS	paragraph 4
Scope	3a	Describe the health condition(s) and population(s) covered by the COS	paragraph 1
	3b	Describe the intervention(s) covered by the COS	paragraph 1
	3c	Describe the setting(s) in which the COS is to be applied	paragraph 4
METHODS			
Protocol/Registry entry	4	Indicate where the COS development protocol can be accessed, if available and/or the study registration details	paragraph 2
Participants	5	Describe the rationale for stakeholder groups involved in the COS development process, eligibility criteria for participants from each group and a description of how the individuals involved were identified	'Participants' section
Information sources	6a	Describe the information sources used to identify an initial list of outcomes	'Identification of outcomes' section

	6b	Describe how outcomes were dropped/combined, with reasons (if applicable)	'Delphi survey' and 'Statistical analysis' sections
Consensus process	7	Describe how the consensus process was undertaken	'Delphi survey' section and Figure 1
Outcome scoring	8	Describe how outcomes were scored and scores summarised	'Delphi survey' and 'Statistical analysis' sections
Consensus definition	9a	Describe the consensus definition	'Delphi survey' section and Figure 1
	9b	Describe the procedure for determining how outcomes were included or excluded from consideration during the consensus process	'Statistical analysis' section and Figure 1
Ethics and consent	10	Provide a statement regarding the ethics and consent issues for the study	First paragraph of 'Ethics' section
RESULTS			
Protocol deviations	11	Describe any changes from the protocol (if applicable), with reasons, and a describe what impact these changes have on the results	Deviations from the protocol are outlined in the 'Delphi survey – stage 1/outcome selection' , 'Instrument selection' and 'Delphi survey – stage 2/instrument selection' sections

Participants	12	Present data on the number and relevant characteristics of the people involved at all stages of COS development	Figure 1 and throughout 'Results'
Outcomes	13a	List all outcomes considered at the start of the consensus process	Outcomes listed in Appendix 3 and Appendix 4 in Supplementary information.<br><br>Instruments listed in Appendix 11 and Appendix 12 in Supplementary Information.
	13b	Describe any new outcomes introduced and any outcomes dropped, with reasons, during the consensus process	This is described throughout 'Results' section and in Appendices 4, 5, 6, 11 and 14, and in Tables 1A, 1B, 1C, 2 and 4.
Core outcome set	14	List the outcomes in the final core outcome set	Tables 1A and 1B, and Table 4
DISCUSSION			
Limitations	15	Discuss any limitations in the COS development process	paragraphs 4 and 5
Conclusions	16	Provide an interpretation of the final COS in the context of other evidence, and implications for future research	paragraphs 5 and 6
OTHER INFORMATION			
Funding	17	Describe sources of funding, role of funders	'Funding statement' section

Conflicts of interest	18	Describe any conflicts of interest within the study team and how these were managed	'Conflicts of interest' section

[1]Kirkham JJ, Gorst S, Altman DG et al. Core Outcome Set–STAndards for Reporting: The COS-STAR Statement. *PLoS Med* 2016;13(10): e1002148.

Table S2. Stage 1 (outcome selection) expert group composition. The stage 1 expert group comprised of 10 members of the public with experience of National Health Service (NHS), local authority or commercial weight management programmes in the United Kingdom (UK), 10 academics/policy makers/commissioners working in weight management, 10 weight management staff involved in delivering a lifestyle weight management programme for adults (without significant policy involvement), and 10 primary care staff with experience of referring patients to weight management programmes. 6 of 10 (60%) members of the public had experience of commercial behavioural weight management interventions (BWMIs), 6 of 10 were of working age (60%) and 4 of 10 were male (40%). The 10 members of the public represented 9 different UK counties (6 Scottish counties and 3 English counties). 9 of 10 academics/policy makers/commissioners were from England (90%), 4 of 10 were academics (40%), 3 of 10 were policy makers (30%) and 3 of the 10 were commissioners (30%). 7 of 10 primary care staff (70%) and 8 of 10 weight management staff (80%) selected were from England. *From England as opposed to other parts of the UK.

Expert Group Subgroup	Total Number	Experience of Commercial BWMIs	Working Age	Male	From England*	Academic	Policy Maker	Commissioner
Members of Public	10	6	6	4	3	⁄	⁄	⁄
Academics/Policy Makers/Commissioners	10	⁄	⁄	⁄	9	4	3	3
Primary Care Staff	10	⁄	⁄	⁄	8	⁄	⁄	⁄
Weight Management Staff	10	⁄	⁄	⁄	7	⁄	⁄	⁄

Table S3. Stage 2 (outcome measurement instrument/outcome definition selection) expert group composition. The stage 2 expert group comprised of 20 academics/policy makers/commissioners working in weight management and 20 weight management staff involved in delivering a lifestyle weight management programme for adults (without significant policy involvement). 16 of 20 academics/policy makers/commissioners were from England (80%), 11 of 20 were academics (55%), 4 of 20 were policy makers (20%) and 5 of 20 were commissioners (25%). 14 of 20 weight management staff were from England (70%). *From England as opposed to other parts of the UK.

Expert Group Subgroup	Total Number	From England*	Academic	Policy Maker	Commissioner
Academics/Policy Makers/Commissioners	20	16	11	4	5
Weight Management Staff	20	14	/	/	/

Table S4. Stage 1 (outcome selection), rounds 1 and 2 Delphi results. During round 1, 46 of 148 outcomes were rated as being either unimportant or unsure (median rating ≤ 6.5) and were not carried forward to round 2. The remaining 102 outcomes were rated as important (median rating ≥ 7) and were carried forward. With the exception of the 1 month follow-up time point (disagreement index > 1.0) expert group members were in agreement with regard to outcome ratings during round 1. During round 2, 87 of 109 outcomes were rated as important (median rating ≥ 7). The remaining 22 outcomes were rated as unsure (median rating ≤ 6.5 and ≥ 5). There was no disagreement between expert panel members during round 2 (disagreement index < 1.0). IPR, inter-percentile range: IPRAS, inter-percentile range adjusted for symmetry; BMI, body mass index; QoL, quality of life; HbA1c, haemoglobin A1c; NAFLD, non-alcoholic fatty liver disease; FODMAP, fermentable oligo-, di-, mono-saccharides and polyols.

Delphi Round	Timepoint	Domain	Outcome	Importance	Mean Panel Rating	Median Panel Rating	Disagreement Index (IPR:IPRAS)
2	At Follow-up	Length of Follow-up	12 months	Important	8	9	0.13
2	At Follow-up	Programme Specific Outcomes	Attendance	Important	8.3	9	0.13
2	At Baseline	Physical Measurements	BMI	Important	8.3	9	0.13
2	At Follow-up	Physical Measurements	BMI	Important	8.2	9	0.13
2	At Follow-up	Programme Specific Outcomes	Completion	Important	8.5	9	0.13
2	At Baseline	Physical Measurements	Weight	Important	8.7	9	0.00
2	At Follow-up	Physical Measurements	Weight	Important	8.6	9	0.00
2	At Follow-up	Length of Follow-up	24 months	Important	7.5	8	0.29
2	At Follow-up	Programme Specific Outcomes	Adverse Events/Unintended Consequences	Important	7.1	8	0.16
2	At Baseline	Demographics	Age	Important	7.2	8	0.16

2	At Follow-up	Programme Specific Outcomes	Cost Effectiveness	Important	7.3	8	0.29
2	At Follow-up	Psychological Factors	Depression	Important	6.9	8	0.37
2	At Baseline	Comorbidities	Diabetes Status	Important	7.5	8	0.29
2	At Follow-up	Comorbidities	Diabetes Status	Important	7.2	8	0.16
2	At Baseline	Demographics	Gender	Important	6.8	8	0.16
2	At Follow-up	Programme Specific Outcomes	Participant Satisfaction	Important	7.5	8	0.29
2	At Baseline	Psychological Factors	QoL Score	Important	7.2	8	0.16
2	At Follow-up	Psychological Factors	QoL Score	Important	7.2	8	0.16
2	At Follow-up	Programme Specific Outcomes	Reason for Dropout	Important	7.2	8	0.16
2	At Follow-up	Programme Specific Outcomes	Referral to Specialist Services	Important	7.1	8	0.16
2	At Follow-up	Length of Follow-up	18 months	Important	6.6	7	0.37
2	At Follow-up	Length of Follow-up	3 months	Important	6.5	7	0.65
2	At Follow-up	Length of Follow-up	6 months	Important	6.8	7	0.37
2	At Baseline	Psychological Factors	Anxiety	Important	6.2	7	0.52
2	At Follow-up	Psychological Factors	Anxiety	Important	6.3	7	0.65
2	At Baseline	Comorbidities	Asthma	Important	5.9	7	0.52
2	At Baseline	Psychological Factors	Binge Eating Disorder	Important	6.8	7	0.37
2	At Follow-up	Psychological Factors	Binge Eating Disorder	Important	6.5	7	0.37
2	At Baseline	Physical Measurements	Blood Pressure	Important	6.2	7	0.65
2	At Follow-up	Physical Measurements	Blood Pressure	Important	6.5	7	0.37
2	At Baseline	Psychological Factors	Body Image	Important	6	7	0.65
2	At Follow-up	Psychological Factors	Body Image	Important	6.2	7	0.37
2	At Baseline	Comorbidities	Cardiovascular Risk	Important	6.4	7	0.37
2	At Follow-up	Comorbidities	Cardiovascular Risk	Important	6.6	7	0.37

2	At Baseline	Psychological Factors	Confidence in Ability to Lose Weight	Important	6.4	7	0.65
2	At Follow-up	Psychological Factors	Confidence in Ability to Lose Weight	Important	6.4	7	0.65
2	At Baseline	Diet	Daily Alcohol Consumption	Important	5.9	7	0.52
2	At Follow-up	Diet	Daily Alcohol Consumption	Important	5.8	7	0.52
2	At Baseline	Diet	Daily Calorie Consumption	Important	5.8	7	0.52
2	At Follow-up	Diet	Daily Calorie Consumption	Important	5.9	7	0.65
2	At Baseline	Diet	Daily Fruit & Vegetable Intake	Important	6.3	7	0.52
2	At Follow-up	Diet	Daily Fruit & Vegetable Intake	Important	6	7	0.52
2	At Baseline	Psychological Factors	Depression	Important	6.9	7	0.16
2	At Baseline	Demographics	Deprivation Category	Important	6.7	7	0.37
2	At Baseline	Psychological Factors	Disordered Eating	Important	6.5	7	0.37
2	At Follow-up	Psychological Factors	Disordered Eating	Important	6.3	7	0.65
2	At Baseline	Demographics	Ethnicity	Important	6.1	7	0.52
2	At Baseline	Demographics	Family History of Obesity	Important	6	7	0.65
2	At Baseline	Physical Measurements	Fat Mass/Body Composition	Important	5.8	7	0.52
2	At Follow-up	Physical Measurements	Fat Mass/Body Composition	Important	5.9	7	0.52
2	At Baseline	Physical Activity	Fitness	Important	5.9	7	0.52
2	At Follow-up	Physical Activity	Fitness	Important	6.3	7	0.65
2	At Baseline	Comorbidities	High Blood Pressure	Important	7	7	0.16
2	At Follow-up	Comorbidities	High Blood Pressure	Important	6.7	7	0.65
2	At Baseline	Comorbidities	High Cholesterol/ Lipids	Important	6.5	7	0.37

2	At Baseline	Comorbidities	High Future Risk of Diabetes (Impaired Fasting Glucose, Impaired Glucose Tolerance, Raised HbA1c Levels)	Important	6.8	7	0.37
2	At Follow-up	Comorbidities	High Future Risk of Diabetes (Impaired Fasting Glucose, Impaired Glucose Tolerance, Raised HbA1c Levels)	Important	6.5	7	0.37
2	At Baseline	Psychological Factors	Importance of Weight Loss	Important	6.5	7	0.65
2	At Follow-up	Psychological Factors	Importance of Weight Loss	Important	6.4	7	0.65
2	At Baseline	Demographics	Learning Disability	Important	6.2	7	0.22
2	At Baseline	Physical Activity	Leisure Time Physical Activity	Important	6.1	7	0.52
2	At Follow-up	Physical Activity	Leisure Time Physical Activity	Important	6.2	7	0.52
2	At Baseline	Comorbidities	Mobility Issues	Important	6.7	7	0.37
2	At Follow-up	Comorbidities	Mobility Issues	Important	6.3	7	0.65
2	At Baseline	Physical Activity	Non Leisure Time Physical Activity	Important	6	7	0.52
2	At Follow-up	Physical Activity	Non Leisure Time Physical Activity	Important	6.1	7	0.52
2	At Baseline	Lifestyle Behaviours	Other Addictive Behaviour	Important	5.9	7	0.52
2	At Baseline	Comorbidities	Overall Measure of Comorbidity	Important	6.8	7	0.16
2	At Follow-up	Comorbidities	Overall Measure of Comorbidity	Important	6.6	7	0.16
2	At Baseline	Demographics	Physical Disability	Important	6.3	7	0.22
2	At Follow-up	Programme Specific Outcomes	Prescription of Anti-obesity Medication	Important	6.6	7	0.37
2	At Follow-up	Programme Specific Outcomes	Reach	Important	6.5	7	0.65
2	At Follow-up	Programme Specific Outcomes	Referral to Linked Services	Important	6.8	7	0.16

2	At Follow-up	Programme Specific Outcomes	Repeat Referrals	Important	7.1	7	0.16
2	At Follow-up	Programme Specific Outcomes	Representativeness	Important	6.8	7	0.16
2	At Baseline	Physical Activity	Sedentary Time	Important	5.9	7	0.52
2	At Follow-up	Physical Activity	Sedentary Time	Important	6.4	7	0.22
2	At Baseline	Psychological Factors	Self Confidence	Important	6.4	7	0.37
2	At Follow-up	Psychological Factors	Self Confidence	Important	6.6	7	0.37
2	At Baseline	Psychological Factors	Self Esteem	Important	6.4	7	0.65
2	At Follow-up	Psychological Factors	Self Esteem	Important	6.5	7	0.37
2	At Baseline	Lifestyle Behaviours	Smoking Status	Important	6	7	0.52
2	At Follow-up	Programme Specific Outcomes	Sources of Referral	Important	6.6	7	0.37
2	At Baseline	Psychological Factors	Suicidal Thoughts	Important	6	7	0.65
2	At Follow-up	Physical Measurements	Waist Circumference	Important	6.2	7	0.65
2	At Follow-up	Physical Measurements	Waist to Hip Ratio	Important	5.6	7	0.97
2	At Baseline	Demographics	Weight Loss History	Important	5.9	7	0.97
2	At Baseline	Comorbidities	Advised To Lose Weight Prior To Routine Surgery	Unsure	6	6	0.65
2	At Baseline	Psychological Factors	Autism	Unsure	5.6	6	0.52
2	At Baseline	Comorbidities	Chronic Back Pain	Unsure	5.8	6	0.52
2	At Follow-up	Comorbidities	Chronic Back Pain	Unsure	5.6	6	0.97
2	At Baseline	Comorbidities	Chronic Kidney Disease	Unsure	5.6	6	0.52
2	At Follow-up	Diet	Daily Free Sugar Intake	Unsure	5.6	6	0.52
2	At Follow-up	Comorbidities	High Cholesterol/ Lipids	Unsure	6.1	6	0.52
2	At Baseline	Comorbidities	NAFLD	Unsure	5.9	6	0.52
2	At Baseline	Comorbidities	Obstructive Sleep Apnoea	Unsure	5.8	6	0.52

2	At Follow-up	Comorbidities	Obstructive Sleep Apnoea	Unsure	5.7	6	0.52
2	At Baseline	Comorbidities	Osteoarthritis	Unsure	5.9	6	0.52
2	At Follow-up	Lifestyle Behaviours	Other Addictive Behaviour	Unsure	5.6	6	0.52
2	At Baseline	Comorbidities	Other Health Conditions Requiring A Specialist Diet	Unsure	5.8	6	0.52
2	At Baseline	Psychological Factors	Overall Quality of Sleep	Unsure	5.8	6	0.52
2	At Follow-up	Psychological Factors	Overall Quality of Sleep	Unsure	5.9	6	0.52
2	At Baseline	Psychological Factors	Personality Disorders	Unsure	5.6	6	0.52
2	At Baseline	Comorbidities	Polycystic Ovary Syndrome (women only)	Unsure	5.6	6	0.52
2	At Follow-up	Psychological Factors	Self Reported Reduction in Clothes Size	Unsure	5.5	6	0.97
2	At Follow-up	Psychological Factors	Suicidal Thoughts	Unsure	5.7	6	0.97
2	At Baseline	Physical Measurements	Waist Circumference	Unsure	6.2	6	0.65
2	At Baseline	Physical Measurements	Neck Circumference	Unsure	4.7	5	0.32
2	At Follow-up	Physical Measurements	Neck Circumference	Unsure	4.9	5	0.85
1	At Baseline	Diet	Daily Free Sugar Intake	Unsure	6.2	6.5	0.65
1	At Follow-up	Lifestyle Behaviours	Smoking Status	Unsure	6.2	6.5	0.65
1	At Follow-up	Diet	Daily Saturated Fat Intake	Unsure	6.1	6.5	0.52
1	At Follow-up	Comorbidities	Advised To Lose Weight Prior To Routine Surgery	Unsure	6	6.5	0.52
1	At Follow-up	Comorbidities	Osteoarthritis	Unsure	6	6.5	0.22
1	At Follow-up	Comorbidities	Asthma	Unsure	5.9	6.5	0.22
1	At Baseline	Psychological Factors	Social Support	Unsure	5.8	6.5	0.52
1	At Follow-up	Psychological Factors	Social Support	Unsure	5.7	6.5	0.52
1	At Follow-up	Physical Measurements	Heart Rate	Unsure	6.4	6	0.22

1	At Baseline	Physical Measurements	Waist to Hip Ratio	Unsure	6.1	6	0.52
1	At Follow-up	Diet	Daily Carbohydrate Intake	Unsure	6	6	0.52
1	At Follow-up	Diet	Daily Fibre Intake	Unsure	6	6	0.52
1	At Follow-up	Comorbidities	NAFLD	Unsure	6	6	0.52
1	At Baseline	Diet	Daily Carbohydrate Intake	Unsure	5.9	6	0.52
1	At Baseline	Diet	Daily Fibre Intake	Unsure	5.9	6	0.52
1	At Baseline	Physical Measurements	Heart Rate	Unsure	5.9	6	0.52
1	At Baseline	Comorbidities	Infertility	Unsure	5.9	6	0.65
1	At Baseline	Comorbidities	Advised To Follow The Low FODMAP Diet	Unsure	5.8	6	0.97
1	At Follow-up	Comorbidities	Chronic Kidney Disease	Unsure	5.8	6	0.52
1	At Follow-up	Diet	Daily Protein Intake	Unsure	5.8	6	0.52
1	At Follow-up	Diet	Daily Salt Intake	Unsure	5.8	6	0.52
1	At Baseline	Diet	Daily Saturated Fat Intake	Unsure	5.8	6	0.52
1	At Baseline	Demographics	Employment	Unsure	5.8	6	0.52
1	At Baseline	Demographics	Working Pattern	Unsure	5.8	6	0.52
1	At Baseline	Comorbidities	Coeliac Disease	Unsure	5.7	6	0.97
1	At Baseline	Diet	Daily Salt Intake	Unsure	5.7	6	0.52
1	At Follow-up	Comorbidities	Polycystic Ovary Syndrome (women only)	Unsure	5.7	6	0.52
1	At Baseline	Diet	Daily Protein Intake	Unsure	5.6	6	0.52
1	At Follow-up	Comorbidities	Infertility	Unsure	5.6	6	0.52
1	At Baseline	Comorbidities	Psoriasis	Unsure	5.5	6	0.97
1	At Baseline	Psychological Factors	Relationship With Family	Unsure	5.5	6	0.52
1	At Baseline	Psychological Factors	Relationship With Friends	Unsure	5.5	6	0.52
1	At Baseline	Diet	Vegetarian	Unsure	5.5	6	0.97
1	At Follow-up	Comorbidities	Psoriasis	Unsure	5.3	6	0.97
1	At Follow-up	Psychological Factors	Relationship With Family	Unsure	5.3	6	0.52

1	At Follow-up	Psychological Factors	Relationship With Friends	Unsure	5.3	6	0.97
1	At Baseline	Diet	Vegan	Unsure	5.4	5.5	0.97
1	Follow-up Timepoint	Length of Follow-up	1 month	Unsure	5.3	5.5	1.70
1	At Follow-up	Psychological Factors	Autism	Unsure	5.3	5.5	0.32
1	At Follow-up	Psychological Factors	Personality Disorders	Unsure	5.3	5	0.97
1	At Baseline	Demographics	Education	Unsure	5	5	0.97
1	At Baseline	Demographics	Parity (women only)	Unsure	4.4	4.5	0.97
1	At Baseline	Demographics	Marital Status	Unsure	4.3	4.5	0.52
1	At Baseline	Demographics	Housing Tenure	Unimportant	4	3.5	0.52
1	At Baseline	Demographics	Religion	Unimportant	3.5	3	0.65
1	At Baseline	Demographics	Sexual Orientation	Unimportant	2.8	2.5	0.29

Table S5. Additional outcomes suggested by expert group members during stage 1 (outcome selection), round 1 Delphi. Nineteen additional outcomes were suggested by expert group members during the stage 1, round 1 Delphi. The study team decided that 4 of these would be carried forward to the stage 1, round 2 Delphi.

Suggested Outcome	Participant's Reason For Inclusion	Investigators' Decision	Investigators' Reason	Domain(s)	Explanation
Living arrangements	Having others to cook for could positively or negatively affect one's eating choices	Do not include	This is covered in housing and social support questions		
Working pattern	This could be expanded to define level of activity or inactivity in work being done	Do not include	Work related activity is included in physical activity questionnaires		
Goals for attending weight management/patients' dietary goals	No reason given	Do not include	This is not an outcome		
Neck circumference	No reason given	Include		Physical Measurements At First Visit, Physical Measurements At Follow-up	The measurement of the circumference of a participant's neck. Increased neck circumference has been shown to be a useful initial screening tool for overweight/obesity.
Presence of acanthosis	No reason given	Do not include	This is not an outcome		

Signs of lipodystrophy	No reason given	Do not include	This is not an outcome		
Thyroid stimulating hormone	No reason given	Do not include	This is not an outcome		
Edmonton obesity severity score measured overall	No reason given	Include as 'Overall Measure of Comorbidity'	Edmonton score is an instrument as opposed to an outcome so change additional outcome to 'Overall Measure of Comorbidity'	Comorbidities, Changes In Comorbidities	Using a standard scale, a measurement of how affected a participant is overall by the presence of conditions, diseases or disorders they have in addition to overweight/obesity.
Self reported reduction in clothes size	No reason given	Include		Physical Measurements At Follow-up	Whether a participant is wearing clothes in a smaller size due to weight loss
Frequency of bouts of activity in the day	No reason given	Do not include	This is covered under physical activity		
Frequency of consumption of confectionary	No reason given	Do not include	This is covered under free sugar intake		
Vitamin D deficiency	No reason given	Do not include	This is not an outcome		
Previous bariatric surgery	No reason given	Do not include	Individuals who have previously had bariatric surgery would not be attending BWMPs as they require specialist dietary advice		

Other measures of disordered eating e.g. night eating, secret eating, bulimia etc	No reason given	Include	Add 'disordered eating' outcome	Psychological Factors At Baseline, Changes In Psychological Factors	Disturbed and unhealthy eating patterns including, secret eating, night eating etc
Shortness of breath on exertion - a fitness test with a before and after measure	No reason given	Do not include	This is covered under fitness		
How far dietary goals, set at initial visit, have been met	No reason given	Do not include	This is covered by diet outcomes		
An accepted definition of diabetes remission e.g. at post intervention and 12 months post intervention, diabetes blood markers being used to describe a non diabetic state	No reason given	Do not include	This is covered by 'diabetes status' outcome		
Uptake (number and characteristics of those invited/referred vs those who actually went)	No reason given	Do not include	This is covered by 'attendance' and 'completion' outcomes		
Outcomes relating to anti-obesity medication	In order to see whether its effectiveness warrants the NHS using it	Do not include	The outcome set is specifically for tier 2 programmes so outcomes relating to medications are not relevant		

Table S6. Definitions and instruments identified for measurement and reporting of core and optional outcomes.

Table S6A. Potential definitions and instruments for measurement and reporting of core outcomes. Definitions and instruments for the core outcomes selected during the stage 1 Delphi process were identified from systematic reviews and the study team's knowledge. BMI, body mass index; HbA1c, haemoglobin A1c; T2DM, type 2 diabetes mellitus; QoL, quality of life; EQ-5D-5L, EuroQol 5-level EQ-5D version; SF12, 12-Item Short Form Health Survey; SF36, 36-Item Short Form Health Survey; IWQOL-Lite, 31-Item Impact of Weight on Quality of Life; OWLQOL, Obesity and Weight-Loss Quality of Life; PWI-ID, Personal Wellbeing Index–Intellectual Disability; OEQ, Outcomes and Experiences Questionnaire; NHS, National Health Service; FFT, Friends and Family Test.

**CORE**		
**Outcome**	**Definition/Instrument/Presentation (Baseline)**	**Definition/Instrument/Presentation (Follow-up)**
Age	• mean age of participants in years<br>• % of participants in age bands (16-24 years, 25-34 years, 35-44 years, 45-54 years, 55-64 years, 65-74 years, 75+ years)	
Weight	• mean weight of participants in kg	• mean change in participants' weight in kg<br>• mean % weight change of participants<br>• % of participants achieving ≥ 3% weight loss<br>• % of participants achieving ≥ 5% weight loss<br>• % of participants achieving ≥ 10% weight loss<br>• % of participants achieving ≥ 3kg weight loss

		• % of participants achieving ≥ 5kg weight loss<br>• % of participants achieving ≥ 10kg weight loss
BMI	• mean BMI of participants<br>• % of participants in BMI categories <25, 25-29.9, 30-34.9, 35-39.9, 40-49.9, 50-59.9, ≥60	• mean change in participants' BMI<br>• % of participants achieving BMI <25<br>• % of participants achieving BMI <30
Diabetes Status	• % of participants with type 1 diabetes mellitus (based on self -report, case record or blood test)<br>• % of participants with type 2 diabetes mellitus (based on self -report, case record or blood test)<br>• Mean HbA1c levels of those participants with T2DM<br>• % of those participants with T2DM who are on insulin<br>• mean number of diabetes medications per participant with T2DM	• mean change in HbA1c levels of those participants with T2DM<br>• mean change in % of participants with T2DM who are on insulin<br>• mean change in number of diabetes medications per participant with T2DM
QoL Score	• mean EQ-5D-5L scores of participants<br>• mean SF12 score of participants<br>• mean SF36 scores of participants<br>• mean IWQOL-Lite score of participants<br>• mean OWLQOL scores of participants	• mean EQ-5D-5L scores of participants<br>• mean SF12 score of participants<br>• mean SF36 scores of participants<br>• mean IWQOL-Lite score of participants<br>• mean OWLQOL scores of participants

Learning Disability QoL Score	• mean PWI-ID score(s) of participants<br><br>• mean score obtained using another suitable instrument	
Adverse Events/Unintended Consequences		number of participants experiencing a worsening of a pre-existing medical condition, such as,<br>• an undiagnosed eating disorder or other pre-existing medical conditions (Please make suggestions in the box below.)<br><br>• number of participants suffering severe hypoglycaemia<br><br>• number of participants sustaining an injury during a physical activity session run by the weight management service<br><br>• number of participants experiencing other side effects (Please make suggestions in the box below.)
Repeat Referrals		• % of participants previously referred to the service, not necessarily having attended any sessions)<br><br>• % of participants answering yes, having previously attended at least 1 weight management session

Attendance		• mean % of core/mandatory sessions attended by participants<br>• % of participants attending 100% of core/mandatory sessions<br>• % of participants attending ≥80% of core/mandatory sessions<br>• % of participants attending ≥70% core/mandatory sessions<br>• % of participants attending ≥50% core/mandatory sessions
Completion		• % of participants who attended 100% of possible/core/mandatory sessions<br>• % of participants who attended 80% of possible/core/mandatory sessions<br>• % of participants who attended 70% of possible/core/mandatory sessions<br>• % of participants who attended 50% of possible/core/mandatory sessions
Reason for Dropout		% of participants who dropped out due to:<br>• dissatisfaction with the intervention (unrelated to weight loss)<br>• poor weight loss<br>• illness/ hospitalisation

		• pregnancy<br>• change in personal circumstances/social reason<br>• moving from the geographical area<br>• any other reason
Participant Satisfaction		• mean OEQ score adapted to suit weight management services<br>• mean NHS FFT score
Cost Effectiveness		• The Public Health England Weight Management Economic Assessment Tool:<br>http://webarchive.nationalarchives.gov.uk/20170110165804/http://www.noo.org.uk/visualisation/economic_assessment_tool<br>• cost / kg (based on mean weight loss)<br>• cost per 'success' with success being 5% weight loss<br>• cost per 'success' with success being 5kg weight loss<br>• cost per 'success' with success being 3% weight loss<br>• cost per kg based on any participant with a change in weight data
Presentation of Results		• report outcomes for all participants attending ≥1 active weight loss sessions (does not include introductory sessions/information sessions about the service)<br>• report outcomes for all participants attending >1 active weight loss session(s) and therefore having weight change

		data (does not include introductory sessions/information sessions about the service)<br><br>• report outcomes for all participants completing the programme

Table S6B. Potential definitions and instruments for measurement and reporting of optional outcomes. Definitions and instruments for the optional outcomes selected during the stage 1 Delphi process were identified from systematic reviews and the study team's knowledge. CVD, cardiovascular disease; TIA, transient ischaemic attack; HDR, high diabetes risk; OGTT, oral glucose tolerance test; CCI, Charlson Comorbidity Index; EOSS, Edmonton Obesity Staging System; HADS, Hospital Anxiety and Depression Scale; PHQ-9, Patient Health Questionnaire-9; ICECAP-A, ICEpop CAPability measure for Adults; WEMWBS, Warwick-Edinburgh Mental Wellbeing Scale; DIET, Dieter's Inventory of Eating Temptations; TFEQ, Three Factor Eating Questionnaire; EDEQ, Eating Disorder Examination Questionnaire; BES, Binge Eating Scale; QEWP, Questionnaire on Eating and Weight Patterns.

OPTIONAL		
**Outcome**	**Definition/Instrument/Presentation (Baseline)**	**Definition/Instrument/Presentation (Follow-up)**
High Blood Pressure	• % of participants with high blood pressure based on patient report/medication/case notes<br>• % of participants with high blood pressure based on blood pressure readings<br>• mean number of blood pressure medications per participant with high blood pressure	• change in mean blood pressure (systolic/diastolic, mmHg)<br>• change in mean number of blood pressure medications per participant with high blood pressure

Blood Pressure	• mean systolic and diastolic blood pressure of participants<br>• % of participants with blood pressure >140/80 mmHg<br>• % of participants on blood pressure medication based on self-report/case records	• change in mean systolic and diastolic blood pressure of participants<br>• change in % of participants with blood pressure >140/80 mmHg<br>• change in % of participants on blood pressure medication based on self-report/case records
Cardiovascular Risk	• % of participants with previous CVD, including myocardial infarction, stroke, TIA, angina and peripheral vascular disease<br>• % of participants with high CVD risk (previous CVD or a high cardiovascular risk score - N.B. information on blood pressure and lipids would be required to calculate the risk score)<br>• % of participants with a high cardiovascular risk score (primary prevention/not those with previous cardiovascular disease)<br>• mean CVD risk score of participants<br>• % of participants on cardiovascular medication(s)<br>• mean number of cardiovascular medications per participant on cardiovascular medication(s)	• % of participants with a high cardiovascular risk score<br>• change in mean cardiovascular risk score of participants<br>• change in % of participants on cardiovascular medication(s)<br>• change in mean number of cardiovascular medications per participant on cardiovascular medication(s)
High Cholesterol/ Lipids	• % of participants with high cholesterol/lipids based on self-report /case records<br>• % of participants on statin/ lipid lowering medication based on self-report/case records	• % of participants with high cholesterol/lipids based on self-report /case records<br>• % of participants on statin/ lipid lowering medication – based on self-report/case records

	• mean total cholesterol/ high density lipoprotein/ triglycerides of participants as obtained via blood test	• mean total cholesterol/ high density lipoprotein/ triglycerides of participants as obtained via blood test
High Future Risk of Diabetes (Impaired Fasting Glucose, Impaired Glucose Tolerance, Raised HbA1c Levels, Previous Gestational Diabetes)	• % of participants with a medical record of HDR<br>• % of participants with HDR as determined by an OGTT<br>• % of participants with HDR as determined by measuring HbA1c levels	• % of participants with a medical record of HDR (HDR is followed-up annually in primary care)<br>• % of all participants with HDR as determined by OGTT<br>• % of those participants identified as having HDR at baseline who still have HDR as determined by OGTT<br>• % of all participants with HDR as determined by measuring HbA1c levels<br>• % of those participants identified as having HDR at baseline who still have HDR as determined by HbA1c levels

Overall Measure of Comorbidity	• Mean CCI score<br>• mean EOSS score<br>• mean Chronic Disease Score<br>• mean number of dispensed medications per participant	• mean CCI score<br>• mean EOSS score<br>• mean Chronic Disease Score<br>• mean number of dispensed medications per participant
Depression	• % of participants with depression based on patient report/medication/case notes<br>• % of participants on medication for depression<br>• mean HADS questionnaire score of participants<br>• mean PHQ-9 questionnaire score of participants<br>• mean Beck Depression Inventory score of participants	• % of all participants on medication for depression<br>• % of those patients identified as having depression at baseline on medication for depression<br>• mean HADS questionnaire score of participants<br>• mean PHQ-9 questionnaire score of participants<br>• mean Beck Depression Inventory score of participants

Self-confidence & Self-esteem	• mean Tennesse Self-concept Scale score<br>• mean Rosenberg Self-esteem Scale score<br>• mean General Well-being Schedule score<br>• mean ICECAP-A score<br>• mean WEMWBS score	• mean Tennesse Self-concept Scale score<br>• mean Rosenberg Self-esteem Scale score<br>• mean General Well-being Schedule score<br>• mean ICECAP-A score<br>• mean WEMWBS score
Importance of Weight Loss	• mean Dieting Readiness Scale score(s)<br>• mean DIET score(s)<br>• mean Self-Efficacy for Eating Behaviours Scale score(s)	• mean Dieting Readiness Scale score(s)<br>• mean DIET score(s)<br>• mean Self-Efficacy for Eating Behaviours Scale score(s)
Disordered Eating	• % of participants with disordered eating (defined as per service)<br>• mean TFEQ score<br>• mean EDEQ score<br>• mean BES score	• % of participants with disordered eating (defined as per service)<br>• mean TFEQ score<br>• mean EDEQ score<br>• mean BES score

	• mean QEWP score	• mean QEWP score
Reach (% eligible population who are referred to/take up weight management service)		For a specific population subgroup of concern, what % of that population has been referred to/ attended the weight management service. Local data (e.g. Quality and Outcomes Framework) can be used to obtain prevalence rates.<br>Population subgroups of interest:<br>• age <30<br>• male<br>• people with T2DM<br>• other subgroups
Representativeness (how similar the people attending the service are to the local eligible population)		• based on age of participants<br>• based on sex of participants<br>• based on BMI of participants<br>• based on deprivation category of participants<br>• based on ethnicity of participants<br>• based on diabetes status of participants<br>• based on other criteria (Please make suggestions in the box below)

Prescription of Anti-obesity Medication	• % of participants on any anti-obesity medication<br><br>• % of participants on specific anti-obesity medications	• % of participants on anti-obesity medication<br><br>• % of participants on specific anti-obesity medications
